# Temporal Statistical Relationship between Regional Cerebral Oxygen Saturation (rSO_2_) and Brain Tissue Oxygen Tension (PbtO_2_) in Moderate-to-Severe Traumatic Brain Injury: A Canadian High Resolution-TBI (CAHR-TBI) Cohort Study

**DOI:** 10.3390/bioengineering10101124

**Published:** 2023-09-25

**Authors:** Alwyn Gomez, Donald Griesdale, Logan Froese, Eleen Yang, Eric P. Thelin, Rahul Raj, Marcel Aries, Clare Gallagher, Francis Bernard, Andreas H. Kramer, Frederick A. Zeiler

**Affiliations:** 1 Department of Human Anatomy and Cell Science, Rady Faculty of Health Sciences, University of Manitoba, Winnipeg, MB R3T 2N2, Canada; 2 Section of Neurosurgery, Department of Surgery, Rady Faculty of Health Sciences, University of Manitoba, Winnipeg, MB R3T 2N2, Canada; 3 Department of Anesthesiology, Pharmacology & Therapeutics, University of British Columbia, Vancouver, BC V6T 1Z4, Canada; 4 Biomedical Engineering, Faculty of Engineering, University of Manitoba, Winnipeg, MB R3T 2N2, Canada; 5 Department of Neurology, Karolinska University Hospital, 171 76 Stockholm, Sweden; 6 Department of Clinical Neuroscience, Karolinska Institutet, 171 76 Stockholm, Sweden; 7 Department of Neurosurgery, University of Helsinki and Helsinki University Hospital, FI-00029 Helsinki, Finland; 8 Department of Intensive Care, Maastricht University Medical Center, 6229 Maastricht, The Netherlands; 9 School of Mental Health and Neurosciences, University Maastricht, 6211 Maastricht, The Netherlands; 10 Section of Neurosurgery, Department of Clinical Neurosciences, University of Calgary, Calgary, AB T2N 1N4, Canada; 11 Department of Clinical Neurosciences, University of Calgary, Calgary, AB T2N 1N4, Canada; 12 Hotchkiss Brain Institute, University of Calgary, Calgary, AB T2N 1N4, Canada; 13 Section of Critical Care, Department of Medicine, University of Montreal, Montreal, QC H3T 1J4, Canada; 14 Department of Critical Care Medicine, University of Calgary, Calgary, AB T2N 1N4, Canada; 15 Centre on Aging, University of Manitoba, Winnipeg, MB R3T 2N2, Canada; 16 Division of Anaesthesia, Department of Medicine, Addenbrooke’s Hospital, University of Cambridge, Cambridge CB2 1TN, UK

**Keywords:** traumatic brain injury, brain tissue oxygen tension, regional cerebral oxygen saturation, near-infrared spectroscopy, multimodal monitoring

## Abstract

Brain tissue oxygen tension (PbtO_2_) has emerged as a cerebral monitoring modality following traumatic brain injury (TBI). Near-infrared spectroscopy (NIRS)-based regional cerebral oxygen saturation (rSO_2_) can non-invasively examine cerebral oxygen content and has the potential for high spatial resolution. Past studies examining the relationship between PbtO_2_ and NIRS-based parameters have had conflicting results with varying degrees of correlation. Understanding this relationship will help guide multimodal monitoring practices and impact patient care. The aim of this study is to examine the relationship between PbtO_2_ and rSO_2_ in a cohort of TBI patients by leveraging contemporary statistical methods. A multi-institutional retrospective cohort study of prospectively collected data was performed. Moderate-to-severe adult TBI patients were included with concurrent rSO_2_ and PbtO_2_ monitoring during their stay in the intensive care unit (ICU). The high-resolution data were analyzed utilizing time series techniques to examine signal stationarity as well as the cross-correlation relationship between the change in PbtO_2_ and the change in rSO_2_ signals. Finally, modeling of the change in PbtO_2_ by the change in rSO_2_ was attempted utilizing linear methods that account for the autocorrelative nature of the data signals. A total of 20 subjects were included in the study. Cross-correlative analysis found that changes in PbtO_2_ were most significantly correlated with changes in rSO_2_ one minute earlier. Through mixed-effects and time series modeling of parameters, changes in rSO_2_ were found to often have a statistically significant linear relationship with changes in PbtO_2_ that occurred a minute later. However, changes in rSO_2_ were inadequate to predict changes in PbtO_2_. In this study, changes in PbtO_2_ were found to correlate most with changes in rSO_2_ approximately one minute earlier. While changes in rSO_2_ were found to contain information about future changes in PbtO_2_, they were not found to adequately model them. This strengthens the body of literature indicating that NIRS-based rSO_2_ is not an adequate substitute for PbtO_2_ in the management of TBI.

## 1. Introduction

Traumatic brain injury (TBI) is the preeminent form of neurotrauma globally and is a leading cause of death and disability worldwide [1,2]. Contemporary management of TBI largely focuses on guideline-based management aimed at global arterial blood pressure (ABP), intracranial pressure (ICP), and cerebral perfusion pressure (CPP) treatment thresholds to try to minimize the ongoing brain injury that occurs in the acute period following the initial event [3,4]. While there have been multiple iterations of this paradigm, there has been little progress in further improving outcomes following TBI [5]. Given the large global burden of this disease, different approaches to the management of TBI are being explored. Attention has shifted towards a precision-medicine-based approach to the management of TBI that incorporates multi-modal cerebral monitoring [6,7,8,9].

The most widely adopted of these a”xili’ry modalities is brain tissue oxygen tension (PbtO_2_), with recent guidelines incorporating its use into treatment algorithms [4]. Additionally, at least three large phase 3 randomized control trials are underway to evaluate the ability of PbtO_2_ monitoring to improve outcomes following TBI [10,11,12]. PbtO_2_ measures diffusible oxygen content in the extracellular space through a Clarke-type electrode. As a result, it is speculated that it has a slower response to physiologic variations than other monitoring modalities. This also necessitates its invasive placement into viable brain tissue and results in its ability to only sample a small volume of brain tissue [13].

Near-infrared spectroscopy (NIRS) as a cerebral monitoring modality, in the setting of TBI, has grown for the past three decades [14]. The non-invasive nature, ease of application, and potential for high spatial resolution of NIRS monitoring are significant advantages of this modality. While there is a robust body of evidence supporting its quantitative relationship with cerebral blood flow, its precise relationship with PbtO_2_ is much less clear, with some studies concluding a strong linear correlation between the modalities in retrospective observational studies [15,16,17,18,19]. Other retrospective observational studies failed to identify any statistical relationship [20,21,22].

One possible etiology of this discrepancy is a failure to leverage time series analysis techniques that allow for the utilization of high-resolution data streams while accounting for the autocorrelative and hierarchical nature of this type of physiologic data. Without accounting for hierarchical and autocorrelation structures in the data, the assumptions of the regression methods utilized in these studies, mainly the independence of samples, is not entirely valid. It can be hypothesized that this may have resulted in an erroneously strong correlation between these parameters. Presented here is a study utilizing the Canadian High-Resolution Traumatic Brain Injury (CAHR-TBI) Research Collaborative database, which is a multi-institutional database of moderate-to-severe TBI patients. A highly unique feature of this dataset is its concurrently measured high-resolution PbtO_2_ and rSO_2_ data, the likes of which have not previously been reported in the literature. The primary objective of this study is to utilize contemporary time series analysis techniques to better characterize the relationship between PbtO_2_ and NIRS-based regional cerebral oxygen saturation (rSO_2_). The secondary objective of this study is to determine if PbtO_2_ can be adequately modeled by rSO_2_ in the setting of moderate-to-severe TBI.

## 2. Materials and Methods

### 2.1. Study Design

Using data from the Canadian High Resolution TBI (CAHR-TBI) Research Collaborative, a retrospective multicenter cohort study utilizing a prospectively collected database of critically ill TBI patients was performed, similar to recently published work from this group. Retrospective analysis was determined to be appropriate based on availability of data. Additionally, given the exploratory and observational nature of the study, an entirely prospective study would not be particularly beneficial at this time. Patients included in this study were admitted to one of the following university-affiliated hospitals: Vancouver General Hospital (University of British Columbia), Foothills Medical Centre (University of Calgary), and Health Sciences Centre Winnipeg (University of Manitoba). Institutions collaborating in this research have committed to prospective collection of high-resolution physiologic data in TBI patients. Research ethics approval at the University of Manitoba has been obtained for this database (H2017:181 and H2017:188). Additionally, ethics approval was obtained for retrospective access to the database, as well as for anonymous data transfer between centers (H2020:118, H20-03759 and REB20-0482).

### 2.2. Patient Population

The CAHR-TBI database is composed of moderate-to-severe TBI patients (defined as having a Glasgow Coma Scale ranging from 3 to 12) treated at an adult intensive care unit (ICU). All patients in this database had invasive ICP and ABP monitoring and were cared for using management strategies based on Brain Trauma Foundation (BTF) guidelines [3]. PbtO_2_ was managed based on local practice norms which varied from aggressive management to purely observational. PbtO_2_ monitors were placed into viable brain tissues based on CT scans or under direct inspection in the operating room. NIRS-based rSO_2_ was not actively used to guide management at any of the institutions. While granular patient-specific data are not available, similar vasopressor, sedative, and hyperosmolar/hypertonic agents were utilized at all participating institutions. Patient data were entered into the database from 2011 to 2022.

Included in this study were all patients in the CAHR-TBI database that had concurrent invasive PbtO_2_ monitoring and NIRS-based rSO_2_ monitoring. Those without PbtO_2_ or NIRS monitoring were excluded. Age, biological sex, admission Glasgow Coma Score (GCS), admission pupil exam, and follow-up Glasgow Outcome Score (GOS) were extracted. Sample size calculations were not possible and therefore not performed due to the exploratory nature of this study.

### 2.3. High-Resolution Physiologic Data Collection

High-resolution physiologic data-streams included ICP, ABP, and PbtO_2_, as well as left and right rSO_2_. ABP was measured utilizing radial arterial lines. ICP was monitored using intra-parenchymal strain gauge probes (Codman ICP MicroSensor; Codman & Shurtlef Inc., Raynham, MA, USA) placed in the frontal lobe or using external ventricular drains (Medtronic, Minneapolis, MN, USA). PbtO_2_ was measured using intra-parenchymal brain tissue oxygenation probes (Licox Brain Tissue Oxygen Monitoring System; Integra LifeSciences Corp., Plainsboro, NJ, USA) placed in viable frontal lobe tissue. rSO_2_ was measured using NIRS regional cerebral oximetry of both the left and right frontal lobes (Covidien INVOS 5100C or 7100) when possible.

Data streams were recorded in digital high-frequency time series (≥100 Hz for ABP and ICP, 1 Hz for PbtO_2_ and rSO_2_) using analogue-to-digital signal converters (Data Translations, DT9804 or DT9826) when required. This digitized data were linked and stored in time series using Intensive Care Monitoring (ICM+) software (Version 8.5, Cambridge Enterprise Ltd., Cambridge, UK). For the purposes of this study ICP and ABP were included for the sake of cohort characterization and were not utilized in subsequent data analysis.

### 2.4. Physiologic Data Cleaning and Processing

High-resolution physiologic data were artifact-cleared manually by a qualified clinician utilizing ICM+ software. Artifacts were determined through the examination of waveforms for ICP and ABP. Additionally, sudden drops in PbtO_2_ and rSO_2_ to zero were deemed artifactual. All data were cleaned without knowledge of patient demographics or study objectives.

All high-resolution data streams were processed into both 10-seconds-by-10-seconds and minute-by-minute data utilizing ICM+ software. Data were then exported as comma-separated value (CSV) files for data analysis. The minute-by-minute data were utilized throughout the analysis as it is generally considered the standard for cerebral multimodal monitoring signal analytics as it provides a good balance between data size/computational time and temporal resolution [23,24]. The exception was the cross-correlative analysis, where the 10-seconds-by-10-seconds data were also used to confirm the findings from the minute-by-minute data and in the impulse response function plots to evaluating the data at a temporal resolution and frequency in keeping with vasomotion [25]. Due to the side of PbtO_2_ probe placement not being available for all patients, rSO_2_ values on the right were utilized for analysis, unless not available, in which case rSO_2_ values from the left were utilized.

### 2.5. Physiologic Data Analysis and Statistical Methods

#### 2.5.1. Overview

The data analysis was performed using R statistical software (Version 4.2.2, R Foundation for Statistical Computing, Vienna, Austria) with the following packages: *astsa*, *blandr*, *forecast*, *lmtest*, *nlme*, *tidyverse*, *tseries,* and *zoo*. The Intel oneAPI Math Kernel Library (Intel Corp., Santa Clara, CA, USA) was utilized for the Basic Linear Algebra Subprograms (BLAS) and the Linear Algebra Package (LAPACK) to improve computational performance. Data streams were further filtered to exclude PbtO_2_ values less than 0 mmHg and greater than 60 mmHg, as these values were felt likely to be erroneous based on clinical expertise. rSO_2_ values less than 25% were excluded, as this is the lower limit of output for the INVOS devices used. This, along with the previously mentioned manual artifact clearing, resulted in discontinuities in the data streams. As subsequent time series analysis required continuous data streams, discontinuities were filled through basic linear interpolation through the *approx()* function. For all models, alpha was set to 0.05 without correction for multiple comparisons. Additionally, no sample size or power calculations were performed. This was due to the exploratory nature of this study, which leveraged available data sets.

In this study, the relationship between PbtO_2_ and rSO_2_ was characterized through time series analysis and inferential statistical modeling. Linear regression assumes independent sampling. In the setting of frequent resampling from individual subjects, as is the case in all high-frequency cerebral physiologic monitoring, this assumption is invalid in two regards. Resampling from the same subject in a cohort leads to a hierarchical structure, as samples from the same subject are likely to be more similar than those taken between subjects. This is because intrasubject sampling has random unaccounted-for effects held constant that are not constant in intersubject sampling. Beyond this, when samples are taken with a high frequency, there is a tendency for samples to be correlated with previously taken samples from the same subject. This is known as autocorrelation. The simplest means by which to reinstitute the validity of these assumptions of linear regression is to average the data over large epochs of time (i.e., such as daily or over the entire recording period) for each subject and then regress over these averages. In the dynamic setting of the critically ill TBI patient, this can result in a significant loss of information.

Fortunately, there are statistical methods to account for these deviations from the assumption of independent sampling. In this study, two such methodologies were utilized. First, hierarchical linear modeling, also known as linear mixed-effects modeling, can help account for the random effects experienced by each subject. Additionally, time series-based autoregressive integrative moving average (ARIMA) modeling can account for the autocorrelative structure of the modeled data stream, in this case PbtO_2_. The details of this analysis are described further in the subsequent sections. However, an in-depth review of the theoretical background of these methodologies is beyond the scope of this paper. We refer the interested reader to previous works on this subject [26,27,28,29], and literature applying such methodologies to cerebral physiologic data [30].

#### 2.5.2. Determination of Stationarity of Physiologic Data

Prior to performing time series modeling and analysis, it was necessary to determine if the response data streams of interest, PbtO_2_, were stationary. This is necessary, as there is an assumption of signal stationarity in the utilized time series modeling techniques. If signals are not stationary, they must be transformed to be made stationary prior to any modeling. Testing of signal stationarity was accomplished through examination of the autocorrelative function (ACF) plots for each patient’s PbtO_2_ data. For all ACF plots, significance levels were set to a correlation level of +/−(2/N^1/2^), where N is the number of samples. Generally, for each patient, there was no rapid drop off to zero in lag significance, indicative of non-stationarity of the series. This was confirmed with the Augmented Dicky–Fuller (ADF) and Kwiatkowski–Phillips–Schmidt–Shin (KPSS) testing for stationarity.

To make the series stationary, the first difference was taken for each data series. Following first differencing, the stationarity of the data streams was confirmed through inspection of the ACF plots which now showed rapid decay in the significance of subsequent lags. Additionally, ADF and KPSS testing both confirmed stationarity of the first differenced signals. Physiologically, the data streams can now be thought of as the change in PbtO_2_ (ΔPbtO_2_) and the change in rSO_2_ (ΔrSO_2_). While this is not the same as PbtO_2_ and rSO_2_, it was felt that the data obtained from examining how ΔPbtO_2_ relates to ΔrSO_2_ would provide insight into the relationship between these physiologic parameters.

#### 2.5.3. Cross-Correlative Relationship between ΔPbtO_2_ and ΔrSO_2_

Given that PbtO_2_ samples the extracellular fluid of brain parenchyma and NIRS-based rSO_2_ reflects changes in the brain microvasculature, it is conceivable that changes in these parameters would be asynchronous [31,32]. To identify and examine this potential asynchrony, a cross-correlative analysis was carried out between ΔPbtO_2_ and ΔrSO_2_, utilizing the minute-by-minute data over the entire cohort. The cross-correlation function (CCF) of the minute-by-minute data shows the largest correlative magnitude at a lag of 1 (ΔrSO_2_Lag1). This can be interpreted as ΔrSO_2_ being most strongly correlated with ΔPbtO_2_ a minute later. The CCF plot of the 10-seconds-by-10-seconds data reinforces this conclusion, as the most significant lag is seen at lag 6, corresponding to a one-minute delay between ΔrSO_2_ and ΔPbtO_2_. Since ΔrSO_2_Lag1 was found to contain the most information about ΔPbtO_2_, it was used in subsequent linear modeling.

#### 2.5.4. Vector Autoregressive Modeling and Impulse–Response Function Plots

To further provide insights into the relationship between ΔrSO_2_ and ΔPbtO_2_, impulse–response function (IRF) plots were created based on a multivariate vector autoregressive (VAR) model. These plots examine the modeled response of ΔrSO_2_ and ΔPbtO_2_ to a sudden impulse of ΔABP. The high-frequency 10-seconds-by-10-seconds data streams of interest (ΔABP, ΔICP, ΔPbtO_2_, and ΔrSO_2_) were utilized as the cerebral vasoactive response was being examined in this analysis and acts on a frequency scale of approximately 0.1 Hz [23,33]. Given the non-stationarity of the original data streams, the differenced data were used. These parameters were used going forward for VAR modeling and generation of the IRF plots.

To determine the appropriate autoregressive order of the VAR model, the Akaike Information Criterion (AIC) was determined for vector autoregressive models of order 1 to 15. There was diminishing marginal improvement in AIC as model order increased past 6. As such, following the principle of parsimony, a VAR model of order 6 was constructed utilizing the *VAR()* function in R. Finally, using the *irf()* function in R, this VAR model was utilized to model and plot the response in ΔPbtO_2_ as well as ΔrSO_2_ of an orthogonal impulse in ΔABP over the subsequent 10 lags.

#### 2.5.5. Hierarchical Linear Modeling of ΔPbtO_2_ from ΔrSO_2_Lag1

Given the hierarchical nature of this dataset, a linear mixed-effects model with random slope and intercept, utilizing the *lme()* function, was performed with ΔPbtO_2_ as the dependent variable and ΔrSO_2_Lag1 as the independent variable. Next, to evaluate whether there was any autocorrelation of the model’s residuals, ACF and partial autocorrelative function (PACF) plots were made of these residuals. As with the ACF plots, for all PACF plots, significance levels were set to a correlation level of +/−(2/N^1/2^), where N is the number of samples. There was an autocorrelative structure to the residuals, indicating that there was unaccounted for autocorrelation in the response variable, ΔPbtO_2_.

#### 2.5.6. Modeling of ΔPbtO_2_ from ΔrSO_2_Lag1 Accounting for Autocorrelative Structure

Population-level linear mixed-effects models that incorporated the autocorrelative structure of ΔPbtO_2_ were constructed, as has been done in previous cerebral physiology studies [34]. However, given the size of this dataset, the computational times were unacceptably long, with models failing to converge even after weeks of computation. As a result, it was determined that the next best option was to construct a linear model that accounted for the autocorrelative structure of ΔPbtO_2_ for each individual subject and make inferences about the relationship between PbtO_2_ and rSO_2_ based on the general findings of these models.

For each subject, an initial simple linear regression was performed with ΔPbtO_2_ as the dependent variable and ΔrSO_2_Lag1 as the independent variable. In order to account for the autocorrelative nature of ΔPbtO_2_, the ARIMA structure of the residuals needed to be determined. This was done through the *auto.arima()* function for the residuals of the linear model in each subject. Next, the autoregressive and moving average orders were utilized in the *arima()* and *sarima()* functions, with ΔPbtO_2_ as the response variable and ΔrSO_2_Lag1 as the external regressor, to produce a linear model that accounted for the autocorrelative structure of ΔPbtO_2_. For each subject, the significance the ΔrSO_2_Lag1 coefficient was determined. Additionally, for each model, ACF and PACF plots of the residuals were examined to determine if there was any remaining autocorrelative structure.

#### 2.5.7. Evaluating Model Correlation and Agreement

To examine the correlation between the predicted values of ΔPbtO_2_ and actual values of ΔPbtO_2_ for each subject, a Pearson correlation coefficient was obtained. Next, a Bland–Altman plot was produced to evaluate agreement between the predicted values of ΔPbtO_2_ and actual values of ΔPbtO_2_.

## 3. Results

### 3.1. Cohort Demographics

A total of 20 subjects were included in the study, with a total of 114,136 min of time with concurrent PbtO_2_ and rSO_2_ measurements without interpolation. The full demographic data of the cohort can be found in Table 1.

### 3.2. Determination of the Stationarity of the Physiologic Data

**Figure 1:**
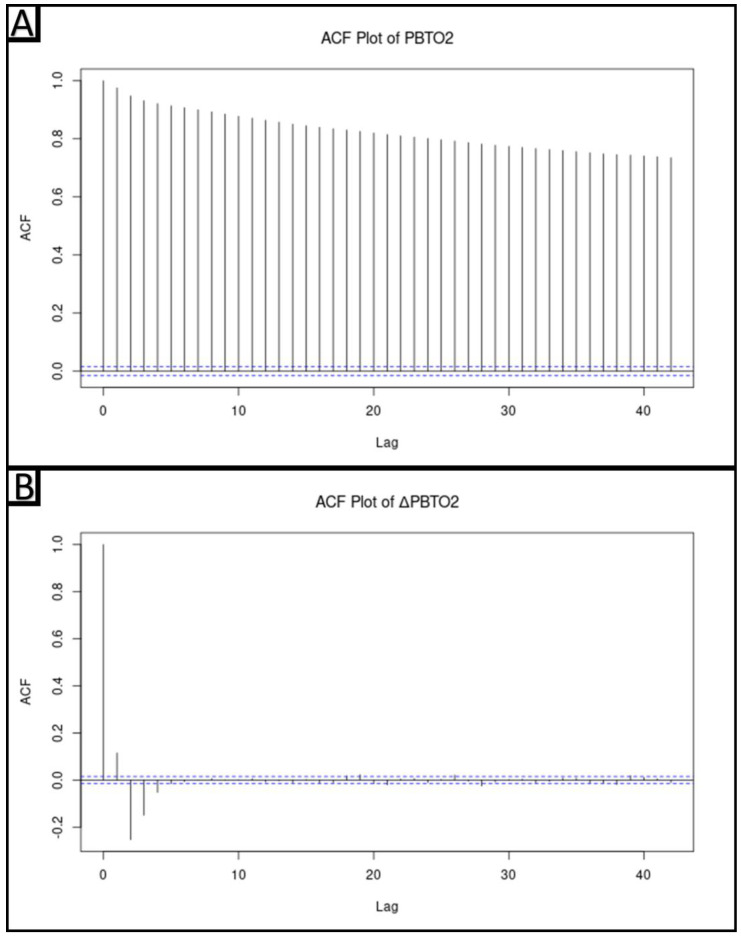
An example autocorrelative function (ACF) plot of PbtO_2_ can be seen in Panel (**A**), indicating non-stationarity, as correlations do not reach zero. Conversely, in Panel (**B**), the ACF plot of ΔPbtO_2_ for the same subject shows a rapid decline in the significant lags, indicating stationarity. The dashed blue line represents the significance levels which were set to a correlation level of +/−(2/N^1/2^), where N is the number of samples. ACF = Autocorrelative Function, ΔPbtO_2_ = Change in brain tissue oxygen tension, PbtO_2_ = Brain tissue oxygen tension.

The ACF plots, as well as the ADF and KPSS test results for each patient’s PbtO_2_ data, can be found in Supplementary File S1. ACF plots did not show a rapid drop-off of significant lags, indicating non-stationarity. An example of an ACF plot can be seen in Figure 1A. KPSS testing for each patient’s PbtO_2_ data also uniformly indicated non-stationarity. In most patients, ADF testing indicated no presence of a unit root. As such, a transformation to the data was required before the assumption of stationarity could be fulfilled and time series models of the data constructed. This pattern of ACF plots and KPSS and ADF testing results is consistent with a difference stationary series, and so the first-order difference of the data was taken to transform the data.

The ACF plots, as well as the ADF and KPSS test results for each patient’s ΔPbtO_2_ data, can be found in Supplementary File S1. Once the first difference was taken, each patient’s ACF plots showed a rapid decline in the significance of lags, indicating stationarity. An example of an ACF plot can be seen in Figure 1B. Consistent with this, KPSS testing indicated stationarity of the ΔPbtO_2_ data for each patient. This indicated that the first-order-differenced data fulfilled the assumptions of stationarity. As a result, subsequent analysis was carried out using both ΔPbtO_2_ and ΔrSO_2_ data.

### 3.3. Cross-Correlative Relationship between ΔPbtO_2_ and ΔrSO_2_

**Figure 2:**
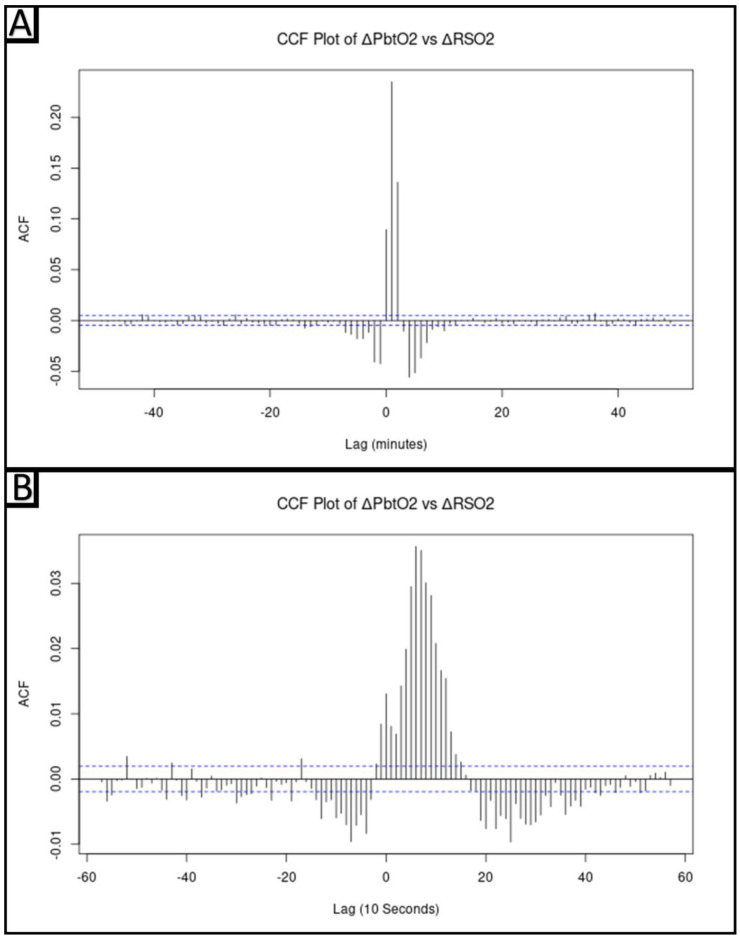
In Panel (**A**), the plot of the cross-correlative function (CCF) of ΔPbtO_2_ vs. ΔrSO_2_ for the minute-by-minute data of the cohort can be seen, with the most significant lag occurring at lag 1 (i.e., 1 min). In Panel (**B**), the plot of the cross-correlative function of ΔPbtO_2_ vs. ΔrSO_2_ for the 10-seconds-by-10-seconds data for the data of the cohort can be seen, with the most significant lag occurring at lag 6 (i.e., at 1 min). The dashed blue line represents the significance levels, which were set to a correlation level of +/−(2/N^1/2^), where N is the number of samples. CCF = Cross-correlative function, ΔPbtO_2_ = Change in brain tissue oxygen tension, ΔrSO_2_ = Change in region cerebral oxygen saturation.

Cross-correlation analysis between ΔPbtO_2_ and ΔrSO_2_, over the entire cohort, indicated the ΔPbtO_2_ was most strongly correlated with ΔrSO_2_ one minute earlier. In other words, ΔPbtO_2_ and ΔrSO_2_Lag1 shared the strongest cross-correlation. In the 10-seconds-by-10-seconds data, a similar pattern was seen with ΔPbtO_2_ correlating with ΔrSO_2_ six lags earlier, equivalent to one minute earlier. The CCF plots of the minute-by-minute data and 10-seconds-by-10-seconds data can be seen in Figure 2A and Figure 2B, respectively. As a result of this finding, ΔPbtO_2_ and ΔrSO_2_Lag1 were utilized for modeling.

### 3.4. Vector Autoregressive Modeling and Impulse–Response Function Plots

**Figure 3:**
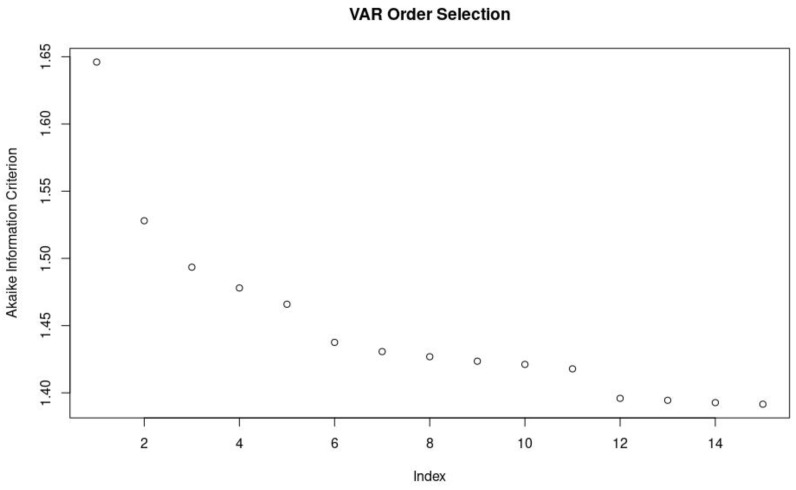
A plot of Akaike information criterion (AIC) versus autoregressive order of the multi-variate vector autoregressive (VAR) model. There is limited improvement in AIC beyond an order of 6.

**Figure 4:**
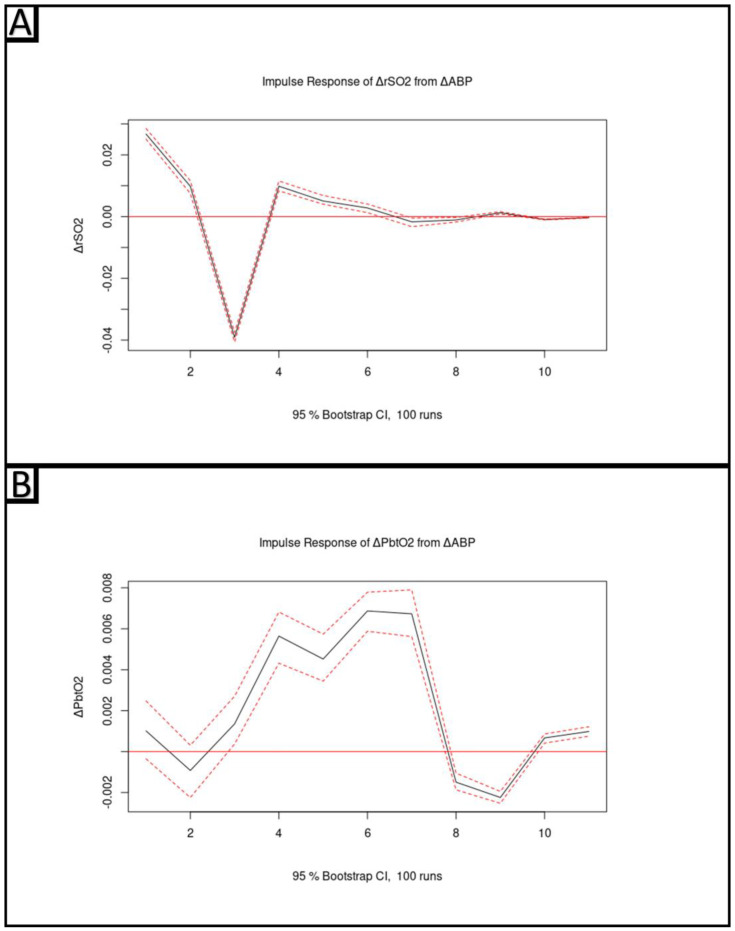
Panel (**A**) shows the modeled resulting response in change in regional cerebral oxygen saturation (ΔrSO_2_) to an orthogonal impulse in change in arterial blood pressure (ΔABP); black solid line. Panel (**B**) shows the modeled resulting response in change in regional cerebral oxygen saturation (ΔPbtO_2_) to an orthogonal impulse in change in arterial blood pressure (ΔABP); black solid line. The 95% confidence intervals are indicated by the red dashed line. Note that in both plots there is an initial rise followed by subsequent decline; however, this is prolonged in the response of ΔPbtO_2_.

The AIC for the multivariate VAR models from order 1 to 15 can be seen in Figure 3. It is clear that there is a clear drop off in marginal improvements in AIC for VAR models with an order greater than 6. As such, a VAR model incorporating ΔABP, ΔICP, ΔPbtO_2_, and ΔrSO_2_ with an order of 6 was utilized to construct the IRF plots. The IRF plots of the modeled response in ΔrSO_2_ and ΔPbtO_2_ to a sudden impulse in ΔABP can be seen in Figure 4A and Figure 4B, respectively. The modeled response of ΔrSO_2_ indicates an almost instantaneous response to an impulse in ΔABP followed by a sharp decrease and eventual return to equilibrium by approximately lag 6. This is in contrast to ΔPbtO_2_, where an impulse in ΔABP results in a delayed and prolonged response that only peaks at approximately lag 6 or 7.

### 3.5. Hierarchical Linear Modeling of ΔPbtO_2_ from ΔrSO_2_Lag1

**Figure 5:**
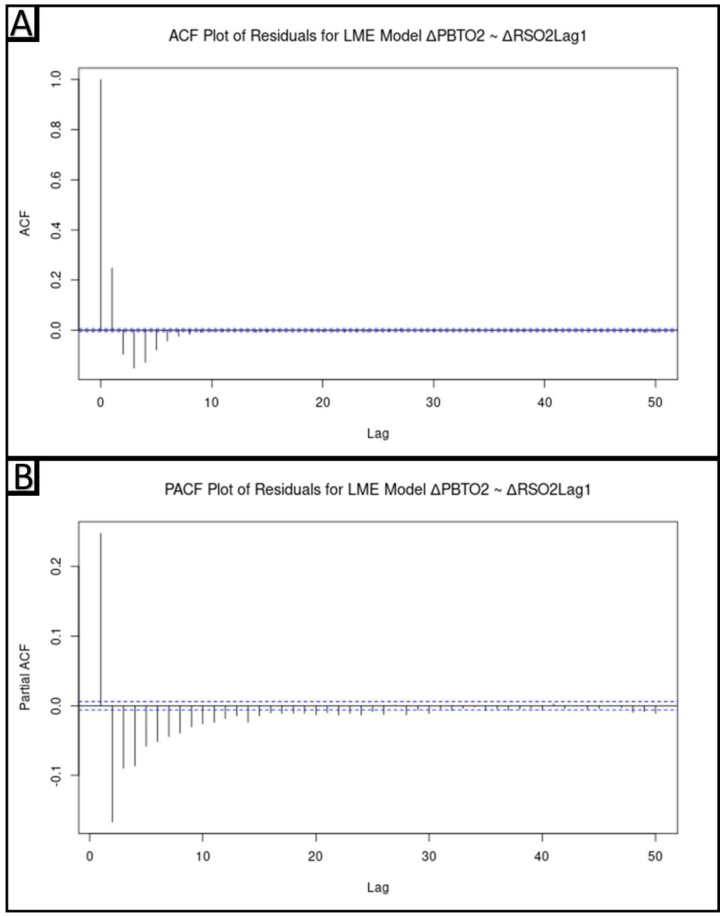
In Panel (**A**), the autocorrelative function (ACF) plot for the residuals of the linear mixed-effects (LME) model (ΔPbtO_2_~ΔrSO_2_Lag1) with random slope and intercept are seen. In Panel (**B**), the partial autocorrelative function (PACF) plot is seen. In both plots, a significant correlation is seen. This is in keeping with a model that has not accounted for the autocorrelative structure of its response variable. The dashed blue line represents the significance levels which were set to a correlation level of +/−(2/N^1/2^), where N is the number of samples. CCF = Cross-correlative function, ΔPbtO_2_ = Change in brain tissue oxygen tension, ΔrSO_2_Lag1 = The one-minute lagged change in regional cerebral oxygen saturation.

The linear mixed-effects model (population level model) with random slope and intercept did find ΔrSO_2_Lag1 to be a significant positive linear regressor of ΔPbtO_2_ (0.35, S.E. 0.10, *p* = 0.0002). However, the ACF and PACF plots can be seen in Figure 5A and Figure 5B, respectively. There is a clear demonstration of autocorrelation of the residuals, indicating an unaccounted-for autocorrelative structure of ΔPbtO_2_. This indicates that the autocorrelative structure of ΔPbtO_2_ needs to be accounted for, prior to valid inferences being made about the relationship between ΔPbtO_2_ and ΔrSO_2_Lag1.

### 3.6. Modeling of ΔPbtO_2_ from ΔrSO_2_Lag1 Accounting for Autocorrelative Structure

Attempts to create a linear mixed-effects model that adequately incorporated the autocorrelative structure of ΔPbtO_2_ at the population level were unsuccessful. This was due to computational complexity with failure to converge. Creating independent inferential linear models for each patient was more successful. The details of these models can be found in Table 2. Of note, ΔrSO_2_ was found to be a significant regressor in 16 of the 20 patients. However, in three of these patients, the coefficient was negative, which is not consistent with the expected relationship between rSO_2_ and PbtO_2_. Examination of the ACF and PACF plots, found in Supplementary File S2, showed minimal autocorrelative structure remaining in the residuals of these models. This confirms model adequacy.

### 3.7. Evaluating Model Correlation and Agreement

The results of the correlation analysis between actual and predicted values of ΔPbtO_2_ are also summarized in Table 2. Notably, correlation coefficients were generally poor for each subject-based model ranging from 0.04 to 0.57. Scatter plots of actual and predicted values of ΔPbtO_2_ for each subject can be found in Supplementary File S3.

**Figure 6:**
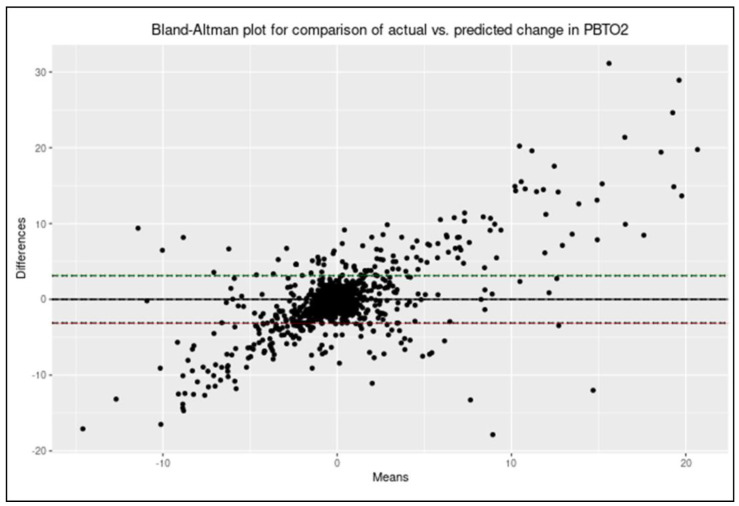
An example from a single subject of the Bland-Altman plot comparing actual and predicted values of ΔPbtO_2_ from the linear ΔPbtO_2_~ΔRSO_2_Lag1 model with autocorrelative structure accounted for. Generally poor agreement can be seen. PbtO_2_ = Brain tissue oxygen tension, ΔPbtO_2_ = Change in brain tissue oxygen tension, and ΔrSO_2_Lag1 = The one-minute lagged change in regional cerebral oxygen saturation.

An example of a Bland–Altman plot for a single model can be seen in Figure 6, with the full series available in Supplementary File S4. Uniformly, agreement was poor throughout all individual subject models of ΔPbtO_2_ from ΔrSO_2_Lag1.

## 4. Discussion

A statistically rigorous exploration of the relationship between the change in PbtO_2_ and change in NIRS-based rSO_2_ was performed in this multi-institutional cohort of 20 moderate-to-severe TBI patients. There are three key insights brought about by this study. First, changes in PbtO_2_ are correlated with changes in rSO_2_ that occur one minute earlier. Second, changes in rSO_2_, in a linear way, contain information about changes in PbtO_2_ that occur one minute later. Finally, while changes in rSO_2_ have this delayed linear relationship with changes in PbtO_2_, changes in rSO_2_ are not adequate for predicting changes in PbtO_2_.

In this study, changes in PbtO_2_ are often best correlated with changes in rSO_2_ one minute earlier. This finding is both consistent with the theoretical mechanisms of each modality and previous findings in the literature. In a study of 42 TBI patients, Budohoski and colleagues noted that NIRS reacted earlier to changes in ABP and ICP as compared to PbtO_2_ [35]. This was recapitulated here in the findings of the VAR-modeled IRF plot. From these plots it can be seen that the response in change in PbtO_2_ is both delayed and prolonged. In the case of change in rSO_2_, it is probable that the initial step rise, subsequent overcorrection, and eventual return to equilibrium may be reflective of cerebrovascular reactivity. The delayed and prolonged nature of the change in PbtO_2_ may explain why continuous cerebrovascular reactivity metrics based on PbtO_2_ have been found to be so dissimilar to those based on more responsive surrogates of cerebral blood flow or volume [36]. As for the mechanism of this delay, PbtO_2_ is a measure of the extracellular content of oxygen in brain tissue as it only measures dissolved oxygen in the interstitial fluid of the brain. NIRS-based rSO_2_ measures microvascular oxygen saturation over a volume of brain as it utilizes deoxyhemoglobin (DeOxHgB) and oxyhemoglobin (OxHgB) as chromophores to scatter and the NIR light [31,32]. Oxygen is primarily delivered to the brain in the form of OxHgB through the brain’s microvasculature. An increase in OxHgB in the brain’s microvasculature would be detected through the absorption of near-infrared light. However, prior to observing a change in oxygen content of the extracellular space of the brain, and therefore PbtO_2_, oxygen would need to disassociate from the OxHgB and diffuse into this extracellular space. A similar delay in decreases in cerebral oxygen content might also be explained by this mechanism. This mechanism is consistent with the findings of changes in PbtO_2_ being correlated with changes in rSO_2_ that occurred one minute earlier. This is a significant finding that may help guide further research into the flow of oxygen through the cerebral microenvironment.

This study found that changes in rSO_2_ may, in a linear way, contain information about a change in PbtO_2_ approximately one minute later. The statistical significance of this relationship held true even when the autocorrelative structure of PbtO_2_ was accounted for. While this was not found in every patient, there are several reasons why this may be the case, the most obvious of which is that NIRS-based rSO_2_ is prone to interference from extravascular blood collections, such as those seen in subgaleal, epidural, and subdural hematomas, as well as intraparenchymal hematomas. In the setting of TBI, these forms of interference are common.

Finally, this study indicates that changes in NIRS-based rSO_2_ are inadequate on their own to predict upcoming changes in PbtO_2_. Despite using a methodology that may tend towards overfitting of the models, measures of change in rSO_2_ were unable to reasonably predict changes in PbtO_2_. This was primarily evident when examining the degree of agreement between actual values of change in PbtO_2_ and predicted values. This adds to the body of evidence that indicates rSO_2_ is not an adequate alternative to PbtO_2_ [20,21,22]. Once again, while rSO_2_ and PbtO_2_ are in some ways measures of cerebral oxygenation, they interrogate entirely different compartments. There are likely several factors that influence changes in PbtO_2_ that were not utilized in the models in this study. Hemoglobin concentrations (HgB), cerebral metabolic rate of oxygen (CMRO_2_), partial pressure of oxygen in the arterial blood (PaO_2_), and microvascular cerebral blood flow velocity (CBFV) are all factors that may modulate how changes in rSO_2_ relate to changes in PbtO_2_. It is understandable why, despite containing information about upcoming changes in PbtO_2_, changes in rSO_2_ could not adequately predict changes in PbtO_2_ in this cohort.

The findings of this study have significant clinical implications. The first is that rSO_2_ and PbtO_2_ provide related but not equivalent information about brain physiology, and as such, rSO_2_, as a raw parameter, is not a viable non-invasive alternative to PbtO_2_ for the monitoring of TBI patients. Further work is needed to better elucidate the potential role of rSO_2_ in the multimodal monitoring of critically ill TBI patients. The related nature of rSO_2_ and PbtO_2_ may be leveraged in the future to improve care, such as in the prehospital setting where invasive monitoring is not possible. Secondly, given the identifiably delayed reaction of PbtO_2_ to changes in ABP as compared to rSO_2_, as demonstrated in the CCF analysis and IRF plots, it is likely that rSO_2_ is better suited for continuous indices of cerebrovascular reactivity. These indices require response parameters that react quickly to changes in ABP. There is a significant degree of interest evolving in the area of continuous cerebrovascular reactivity indices in the monitoring and management of moderate-to-severe TBI.

### 4.1. Limitations

There are limitations to this study that need consideration when interpreting its findings. The first is the relatively small size of the cohort in this study, with only 20 patients included. This necessitates the validation of these findings in a larger cohort before the findings can be fully incorporated into patient care as generalizability might be limited. However, concurrent NIRS-based rSO_2_ and PbtO_2_ monitoring is relatively uncommon, with this study being the first such analysis of high-resolution concurrent recordings in the literature. This cohort represents less than 7% of the full CAHR-TBI database indicating the rarity of simultaneous recordings of these parameters. This likely reflects both the relatively recent global adoption of PbtO_2_ as a means of cerebral monitoring in TBI and the paucity of evidence for the use of NIRS in monitoring moderate-to-severe TBI.

A second limitation, brought on by the type of analysis performed, was the need to interpolate data. This, obviously, injects some inherent uncertainty into the study findings. Another limitation of this study is that information about factors that may interfere with rSO_2_ measurements, such as extravascular blood collections, was not available. Additionally, the side of PbtO_2_ monitor placement needed to be assumed, as this was also not available for all patients.

Finally, the models utilized in this analysis assumed a linear relationship between changes in rSO_2_ and changes in PbtO_2_. This was done due to the lack of evidence suggesting a more appropriate alternative structure to this relationship. It is possible that a mathematically more complex model may prove more suited to describing this relationship. Complex supervised machine learning algorithms might be an obvious method to map this relationship better. While more complex mathematical models might provide an accurate prediction of PbtO_2_ values based on rSO_2_ levels, they are likely to increase computational complexity. This may limit utility at the bedside if computations are not possible in real time due to this increased complexity.

### 4.2. Future Work

The findings of this work lay the groundwork for additional research. First, these findings need to be validated in a larger multi-institutional cohort, where information about sources of NIRS interference is also available. Ideally, additional parameters, including CMRO_2_, PaO_2_, HgB concentration, and microvascular CBFV, would also be concurrently measured to fully elucidate the relationship between these two modalities. With a larger cohort and better characterized physiology, the complexity of this relationship may be better captured.

While NIRS-based rSO_2_ has not gained traction as a stand-alone parameter in the management of TBI, there is increased interest in leveraging NIRS as a means of non-invasively interrogating cerebrovascular reactivity in TBI [37,38,39,40]. This may mean larger datasets with concurrent rSO_2_ and PbtO_2_ monitoring may become available in the future. In such complex multimodal monitoring datasets, supervised and unsupervised classification and regression machine learning algorithms may provide additional insights into how these parameters interact with one another. While complex computational models may not be deployable at the bedside, they may act as inferential models to drive our understanding further. This may ultimately lead to new treatment paradigms in the management of moderate and severe TBI in the acute phase, including specific molecular targets.

## 5. Conclusions

In this multi-institutional exploratory analysis of a cohort of 20 TBI patients with concurrent rSO_2_ and PbtO_2_ monitoring, changes in PbtO_2_ were found to correlate most significantly with changes in rSO_2_ approximately one minute earlier. Through mixed-effects and time series modeling, changes in rSO_2_ were found to often have a statistically significant linear relationship with changes in PbtO_2_ that occurred one minute later. This was the case even when the hierarchical and autocorrelative structure of the data was considered. However, changes in rSO_2_ were inadequate on their own to predict changes in PbtO_2_ based on the poor agreement between modeled and actual changes in PbtO_2_. Given the uniqueness of this dataset, only a small number of subjects were available for analysis, limiting the confidence of these findings. In the future, a larger cohort, with additional parameters that influence cerebral oxygenation, is required to validate and better explain these findings.

## Figures and Tables

**Table 1:** Patient demographics for the cohort.

Demographic Parameter		Median or Number of Subjects
Age (IQR)		41 (34.8–49.3)
Gender	Male subjects (%)	15 (75)
	Female subjects (%)	4 (20)
	N/A (%)	1 (5)
Admission GCS	Eye (IQR)	1 (1–1)
	Verbal (IQR)	1 (1–1)
	Motor (IQR)	2 (1–4)
	Total (IQR)	6 (3–7)
Admission Pupils	Bilaterally Reactive (%)	13 (65)
	Unilaterally Reactive (%)	3 (15)
	Bilaterally Unreactive (%)	3 (15)
	N/A (%)	1 (5)
Marshall CT Classification	I (%)	0 (0)
	II (%)	5 (25)
	III (%)	8 (40)
	IV (%)	0 (0)
	V (%)	4 (20)
	VI (%)	0 (0)
	N/A, n (%)	3 (15)
Follow-up GOS	1 (%)	4 (20)
	2 (%)	0 (0)
	3 (%)	1 (5)
	4 (%)	8 (40)
	5 (%)	3 (15)
	N/A, n (%)	4 (20)
ABP (IQR)		87.0 mmHg (78.6–96.70)
ICP (IQR)		11.0 mmHg (6.7–15.0)
PbtO_2_ (IQR)		24.2 mmHg (17.3–32.3)
rSO_2_ (IQR)		69.6% (63.6–76.8)
PaO_2_ (IQR) *		108 mmHg (88.5–141)
PaCO_2_ (IQR) *		38 mmHg (36–41)

ABP = Arterial Blood Pressure, CT = Computerized Tomography, GCS = Glasgow Coma Scale, GOS = Glasgow Outcome Scale, ICP = Intracranial Pressure, IQR = Interquartile range, N/A = Not available, PbtO_2_ = Brain Tissues Oxygen Tension, and rSO_2_ = Regional Cerebral Oxygen Saturation. * PaO_2_ and PaCO_2_ values were only available for 10 patients.

**Table 2:** Summary of the linear ΔPbtO_2_~ΔrSO_2_Lag1 models with autocorrelative structure accounted for.

Subject ID	Side of rSO_2_	Model Autoregressive Order	Model Moving Average Order	Coefficient of ΔrSO_2_Lag1 as a Regressor (Standard Error)	*p*-Value of ΔrSO_2_Lag1 as a Regressor	Pearson Correlation Coefficient of Actual vs. Predicted ΔPbtO_2_ (95% CI)
1	Right	0	3	−0.012 (0.007)	0.089	0.11 (0.05–0.17)
2	Right	5	1	0.392 (0.017)	<0.001	0.28 (0.25–0.30)
3	Right	6	0	0.080 (0.052)	0.125	0.08 (0.02–0.13)
4	Right	2	2	0.246 (0.034)	<0.001	0.12 (0.10–0.15)
5	Right	3	3	0.242 (0.019)	<0.001	0.19 (0.16–0.21)
6	Right	1	2	1.065 (0.081)	<0.001	0.45 (0.40–0.49)
7	Right	5	1	−0.101 (0.015)	<0.001	0.04 (0.01–0.07)
8	Right	4	3	−0.059 (0.031)	0.055	0.10 (0.05–0.16)
9	Right	2	1	0.131 (0.031)	<0.001	0.10 (0.7–0.14)
10	Right	3	2	−0.059 (0.018)	0.001	0.09 (0.05–0.12)
11	Right	0	2	−0.112 (0.032)	<0.001	0.03 (−0.03–0.09)
12	Right	4	3	1.072 (0.019)	<0.001	0.57 (0.56–0.59)
13	Right	1	3	0.003 (0.002)	0.159	0.14 (0.12–0.15)
14	Left	1	4	0.555 (0.031)	<0.001	0.47 (0.43–0.50)
15	Right	2	2	0.055 (0.015)	<0.001	0.13 (0.11–0.17)
16	Right	3	2	0.168 (0.017)	<0.001	0.13 (0.12–0.16)
17	Right	2	2	0.051 (0.013)	<0.001	0.08 (0.06–0.10)
18	Right	3	1	0.134 (0.001)	<0.001	0.18 (0.16–0.20)
19	Right	1	3	0.760 (0.028)	<0.001	0.40 (0.38–0.41)
20	Right	2	2	0.353 (0.020)	<0.001	0.27 (0.25–0.29)

CI = confidence interval, ΔPbtO_2_ = change in brain tissue oxygen tension, rSO_2_ = regional cerebral oxygen saturation, and ΔrSO_2_Lag1 = the one-minute lagged change in regional cerebral oxygen saturation.

## Data Availability

The raw data supporting the conclusions of this article will be made available by the authors, without undue reservation.

## Supplementary Materials

The following supporting information can be downloaded at: https://www.mdpi.com/article/10.3390/bioengineering10101124/s1, Supplementary File S1: Evaluation of Stationarity of PbtO_2_ Signals, Supplementary File S2: Evaluation of residuals of the linear ΔPbtO_2_~ΔrSO_2_Lag1 models to ensure autocorrelative structure was accounted for, Supplementary File S3: Scatter plots of actual vs. predicted values of ΔPbtO_2_ for each subject, Supplementary File S4: Bland–Altman plots to evaluate model agreement.
