# Supplementary material for: Temporal Statistical Relationship between Regional Cerebral Oxygen Saturation (rSO2) and Brain Tissue Oxygen Tension (PbtO2) in Moderate-to-Severe Traumatic Brain Injury: A Canadian High Resolution-TBI (CAHR-TBI) Cohort Study
Source: Bioengineering (Basel). 2023 Sep 25;10(10):1124. doi: 10.3390/bioengineering10101124 (PMC10604223; doi:10.3390/bioengineering10101124)
Supplement: Supplementary file 1 [file bioengineering-10-01124-s001.zip › bioengineering-2509583-supplementary.pdf]

Supplementary Material

Temporal statistical relationship between regional cerebral oxygen saturation (rSO2) and brain tissue oxygen tension (PbtO2) in moderate to severe traumatic brain injury: A CAnadian High Resolution-TBI (CAHR-TBI) Cohort Study

[1] Department of Human Anatomy and Cell Science, Rady Faculty of Health Sciences, University of Manitoba, Winnipeg, Canada
[2] Section of Neurosurgery, Department of Surgery, Rady Faculty of Health Sciences, University of Manitoba, Winnipeg, Canada
[3] Department of Anesthesiology, Pharmacology & Therapeutics, University of British Columbia, Vancouver, Canada
[4] Biomedical Engineering, Faculty of Engineering, University of Manitoba, Winnipeg, Canada
[5] Department of Neurology, Karolinska University Hospital, Stockholm, Sweden
[6] Department of Clinical Neuroscience, Karolinska Institutet, Stockholm, Sweden
[7] Department of Neurosurgery, University of Helsinki and Helsinki University Hospital, Helsinki, Finland
[8] Department of Intensive Care, Maastricht University Medical Center, Maastricht, The Netherlands; School of Mental Health and Neurosciences, University Maastricht, Maastricht, The Netherlands
[9] Section of Neurosurgery, Department of Clinical Neurosciences, University of Calgary, Calgary, Canada
[10] Section of Critical Care, Department of Medicine, University of Montreal, Montreal, Canada
[11] Department of Critical Care Medicine, University of Calgary, Calgary, Canada
[12] Centre on Aging, University of Manitoba, Winnipeg, Canada
[13] Division of Anaesthesia, Department of Medicine, Addenbrooke's Hospital, University of Cambridge, Cambridge, UK
[14] Department of Clinical Neurosciences, University of Calgary, Calgary, Canada
[15] Hotchkiss Brain Institute, University of Calgary, Calgary, Canada

*** Correspondence:**
Alwyn Gomez BSc MD
Department of Surgery, Rady Faculty of Health Sciences
University of Manitoba
Winnipeg, MB, Canada
Email: gomeza35@myumanitoba.ca
Twitter: @alwyngomez

1 Supplementary File S1: Evaluation of Stationarity of $PbtO_2$ Signals

Supplementary File S1 Table 1: KPSS and ADF test results for each subject

Subject ID	$PbtO_2$		$\Delta PbtO_2$	
**1**	**KPSS**	**ADF**	**KPSS**	**ADF**
**2**	<0.01	0.46	0.08	<0.01
**3**	<0.01	<0.01	>0.1	<0.01
**4**	<0.01	0.19	>0.1	<0.01
**5**	<0.01	<0.01	>0.1	<0.01
**6**	<0.01	<0.01	>0.1	<0.01
**7**	<0.01	<0.01	>0.1	<0.01
**8**	<0.01	0.02	>0.1	<0.01
**9**	<0.01	<0.01	>0.1	<0.01
**10**	<0.01	<0.01	>0.1	<0.01
**11**	<0.01	<0.01	>0.1	<0.01
**12**	<0.01	0.01	>0.1	<0.01
**13**	<0.01	<0.01	>0.1	<0.01
**14**	<0.01	<0.01	>0.1	<0.01
**15**	<0.01	<0.01	>0.1	<0.01
**16**	<0.01	<0.01	>0.1	<0.01
**17**	<0.01	<0.01	>0.1	<0.01
**18**	<0.01	<0.01	>0.1	<0.01
**19**	<0.01	0.07	>0.1	<0.01
**20**	<0.01	<0.01	>0.1	<0.01

ADF = Augmented Dickie Fuller test, KPSS = Kwiatkowski-Phillips-Schmidt-Shin test, ΔPbtO2 = change in brain tissue oxygen tension, $PbtO_2$ = Brain tissue oxygen tension.

<u>Supplementary File S1 Figures 1-20:</u>

Plot Legend: *For the following plots the dashed blue line represents the significance levels which were set to a correlation level of +/-(2/$N^{1/2}$), where N is the number of samples.* *ACF = Autocorrelative function, ΔPbtO2 = change in brain tissue oxygen tension, PbtO2 = Brain tissue oxygen tension*

Subject 1

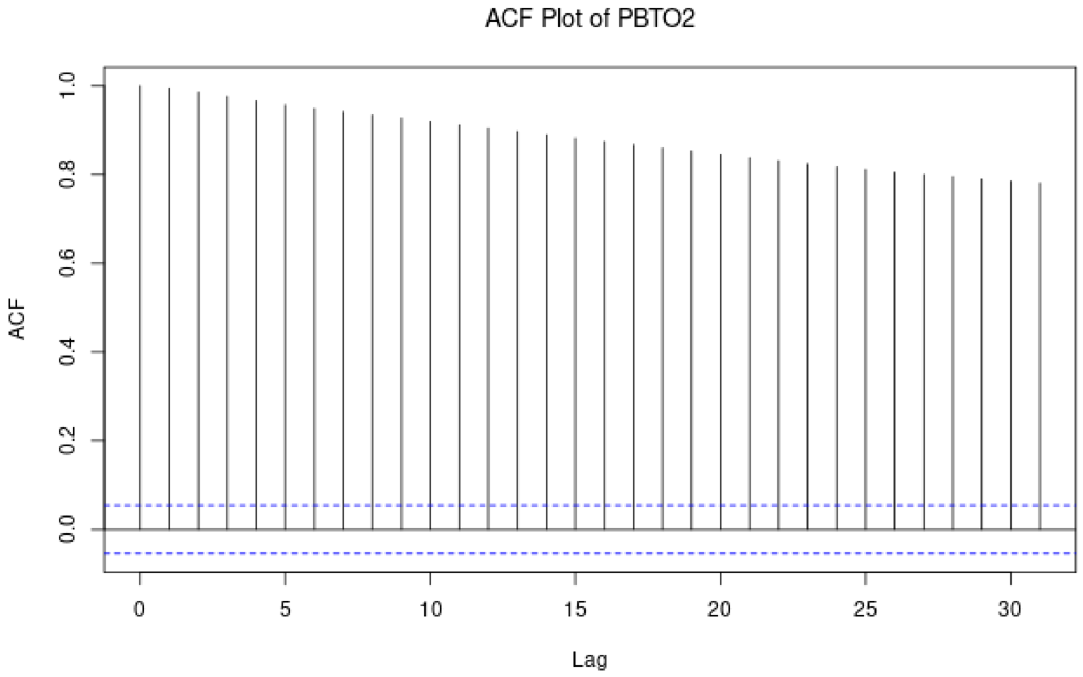

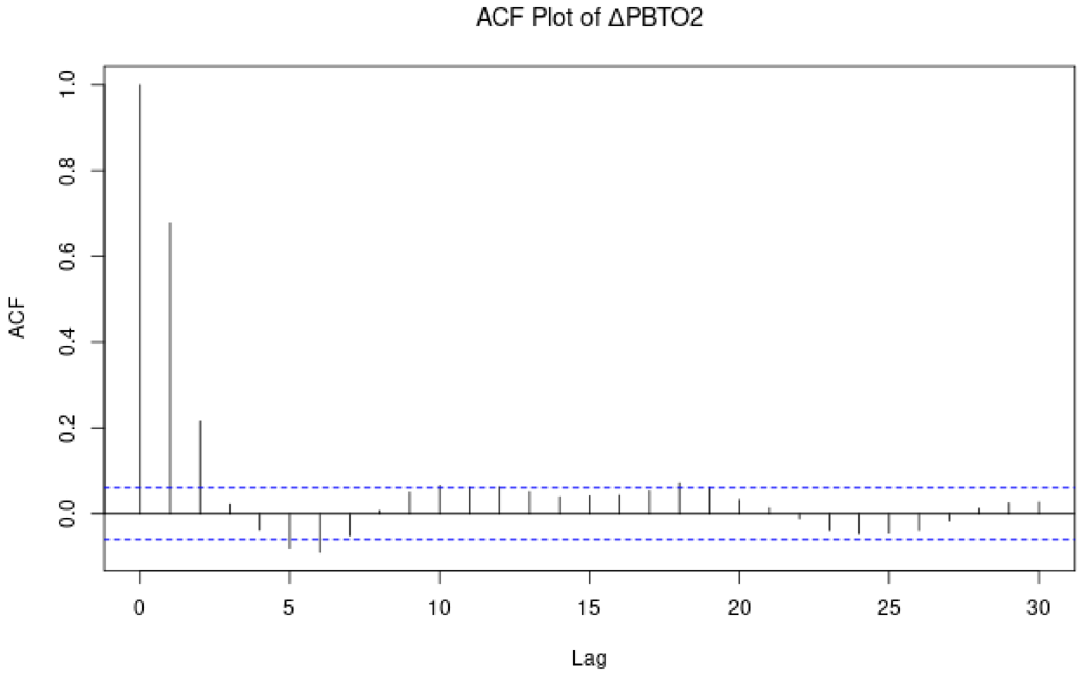

Supplementary File S1 Figure 1: ACF Plots of PbtO$_2$ (Top) and ΔPbtO$_2$ (Bottom) for Subject 1.

Subject 2

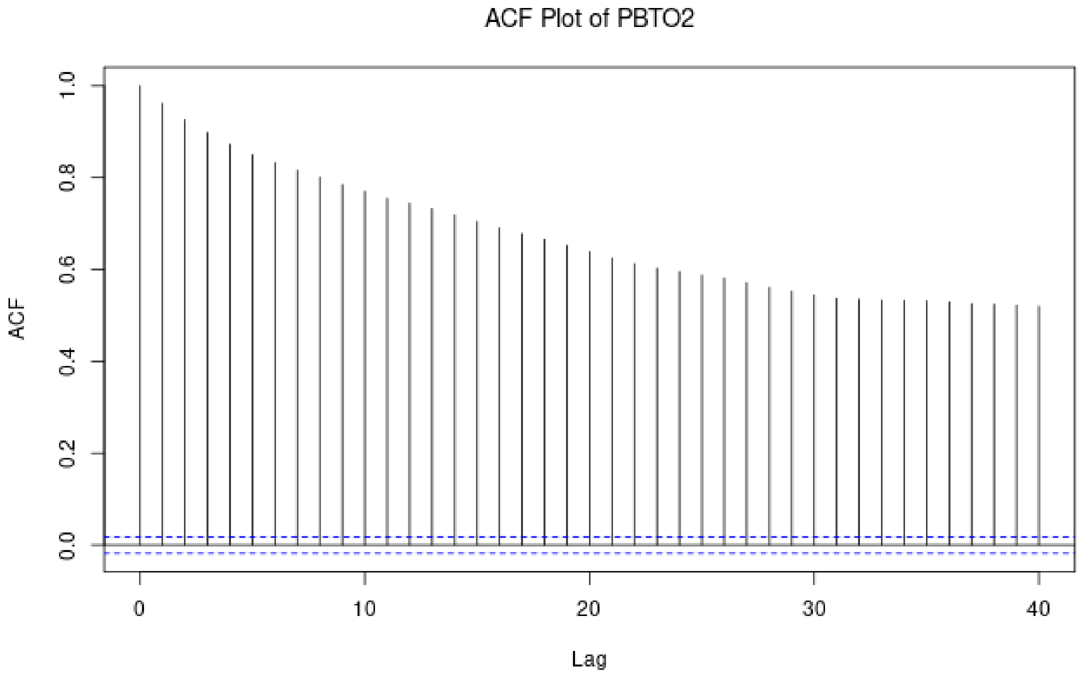

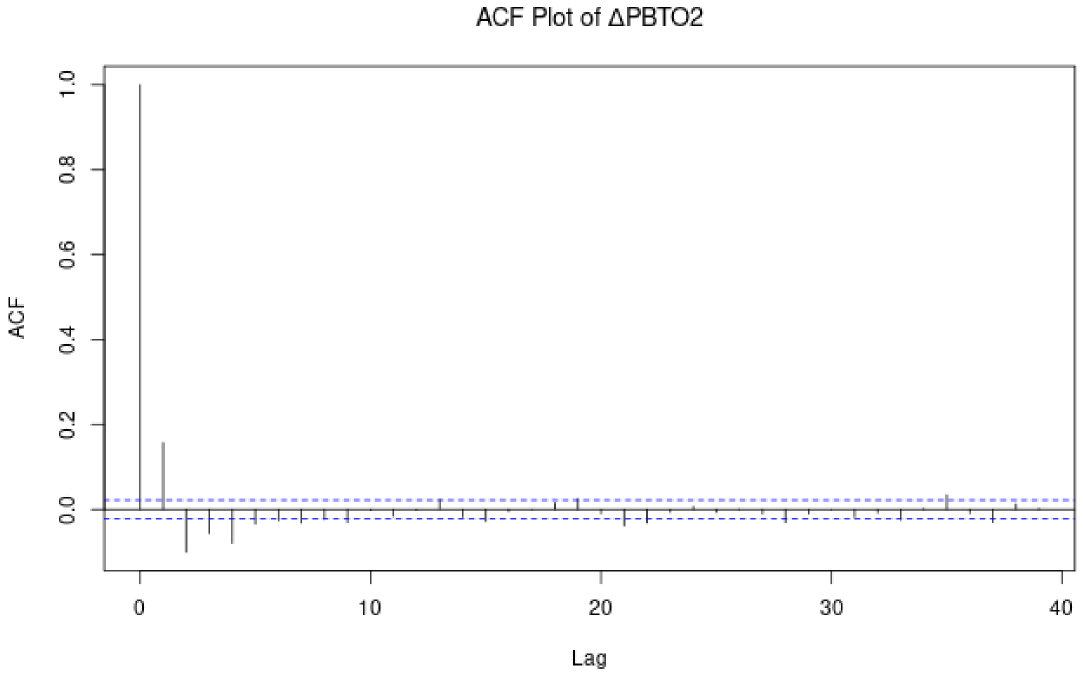

Supplementary File S1 Figure 2: ACF Plots of $PbtO_2$ (Top) and $\Delta PbtO_2$ (Bottom) for Subject 2

Subject 3

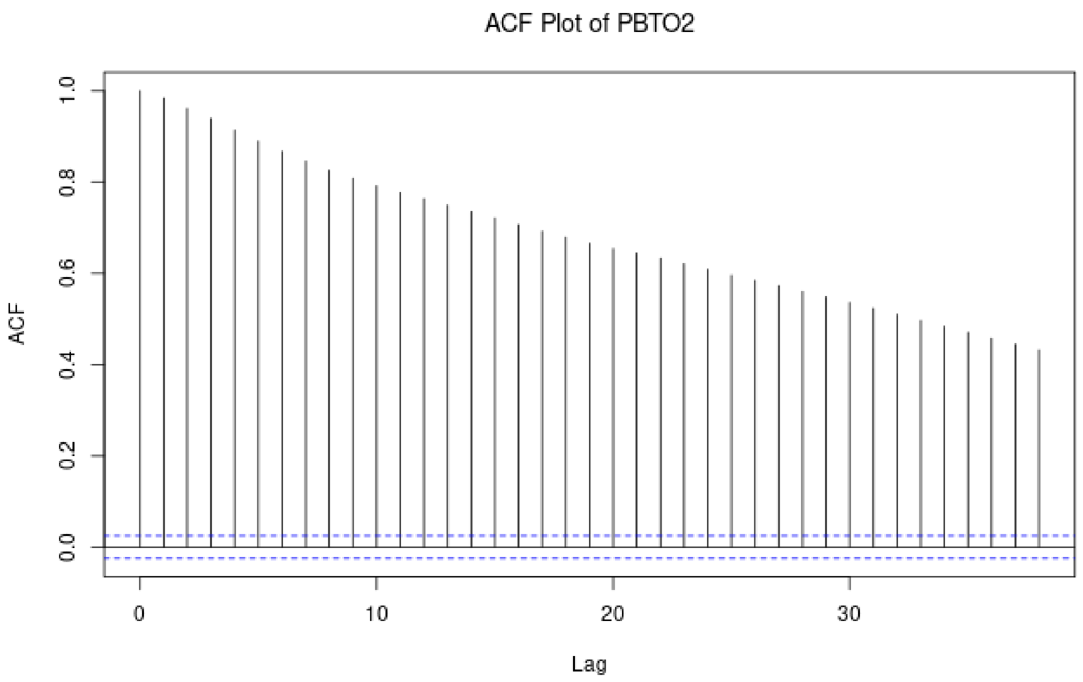

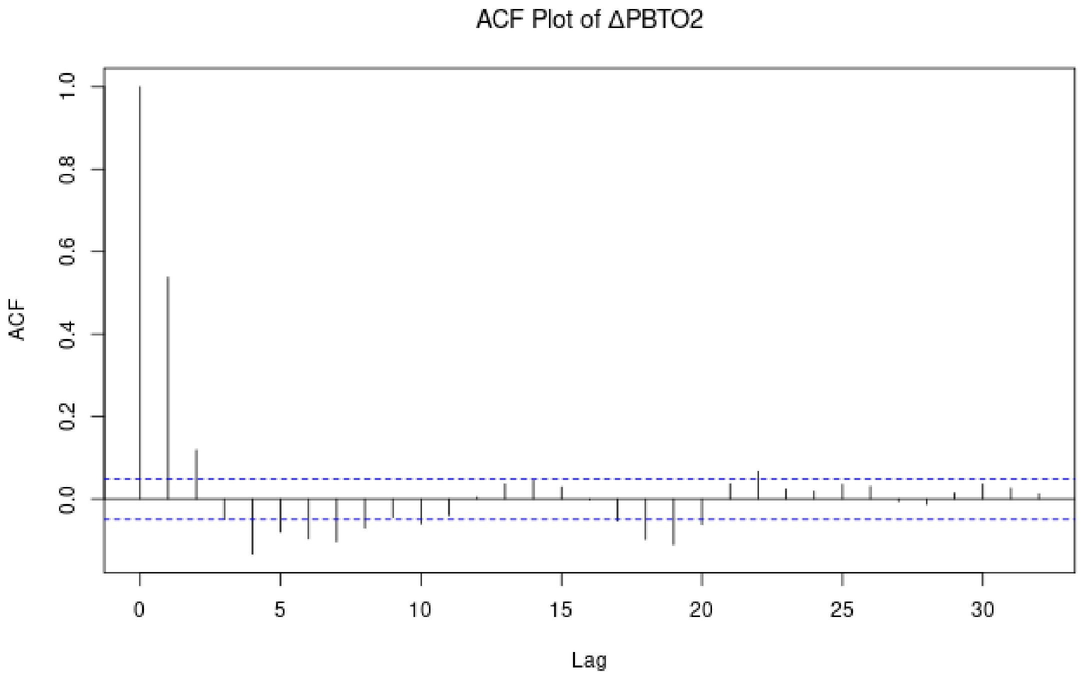

Supplementary File S1 Figure 3: ACF Plots of $PbtO_2$ (Top) and $\Delta PbtO_2$ (Bottom) for Subject 3

Subject 4

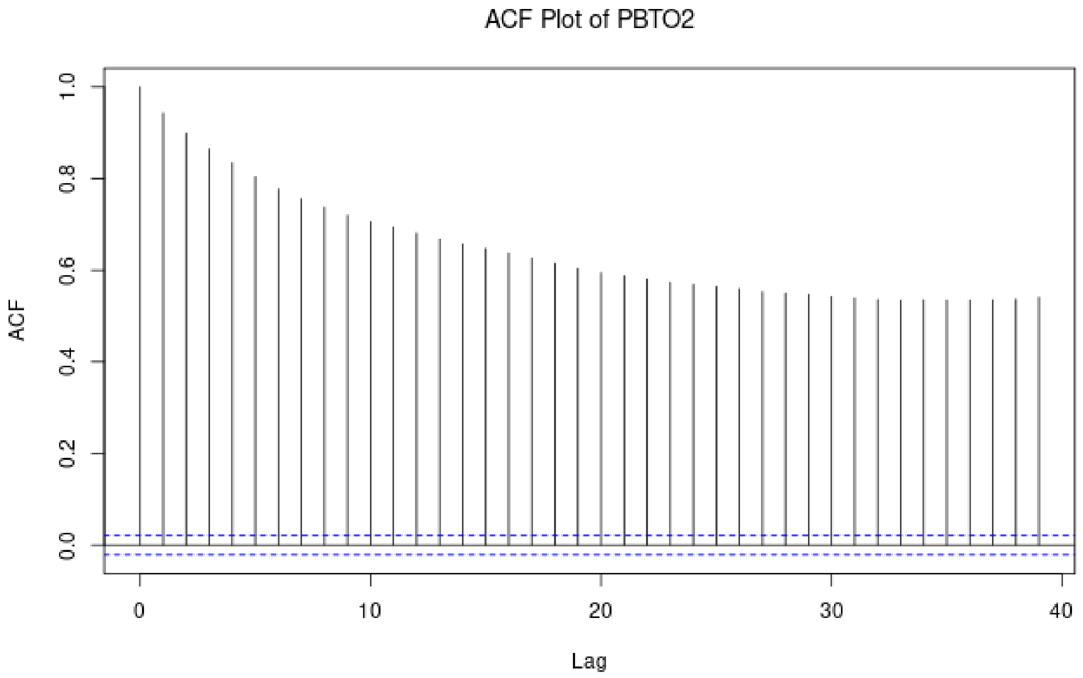

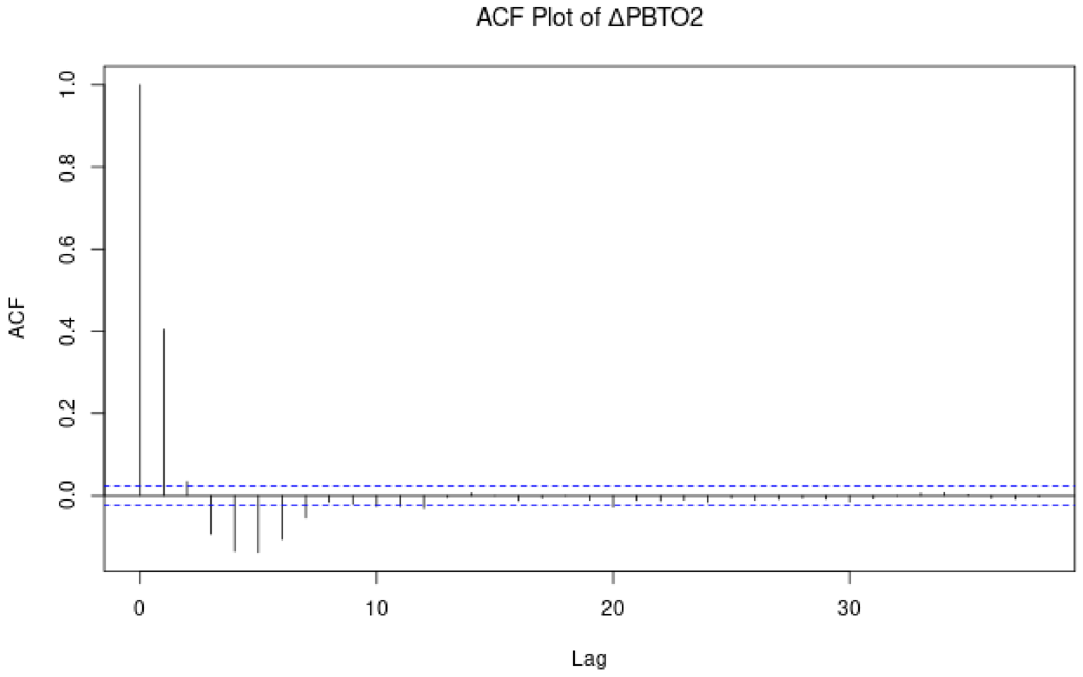

Supplementary File S1 Figure 4: ACF Plots of $PbtO_2$ (Top) and $\Delta PbtO_2$ (Bottom) for Subject 4

Subject 5

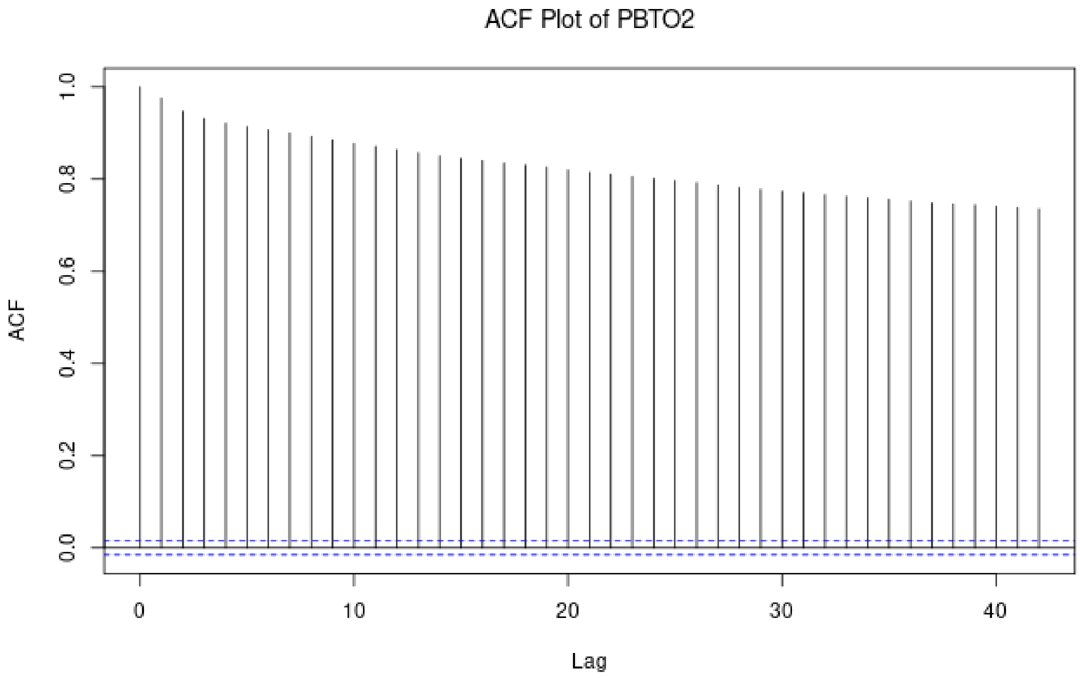

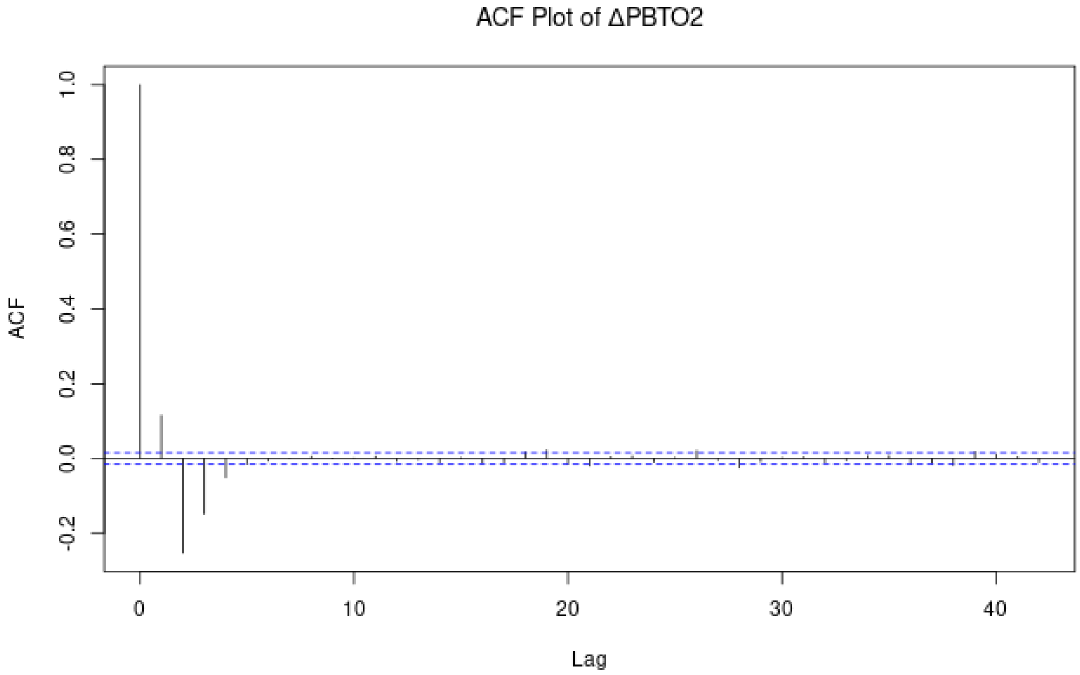

Supplementary File S1 Figure 5: ACF Plots of $PbtO_2$ (Top) and $\Delta PbtO_2$ (Bottom) for Subject 5

Subject 6

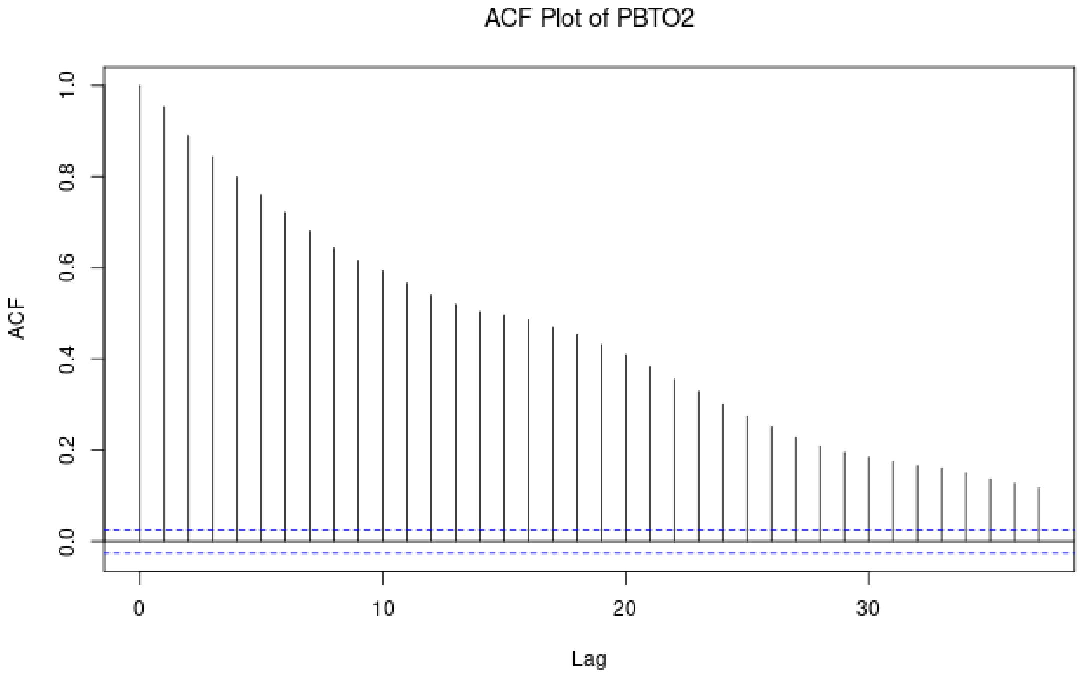

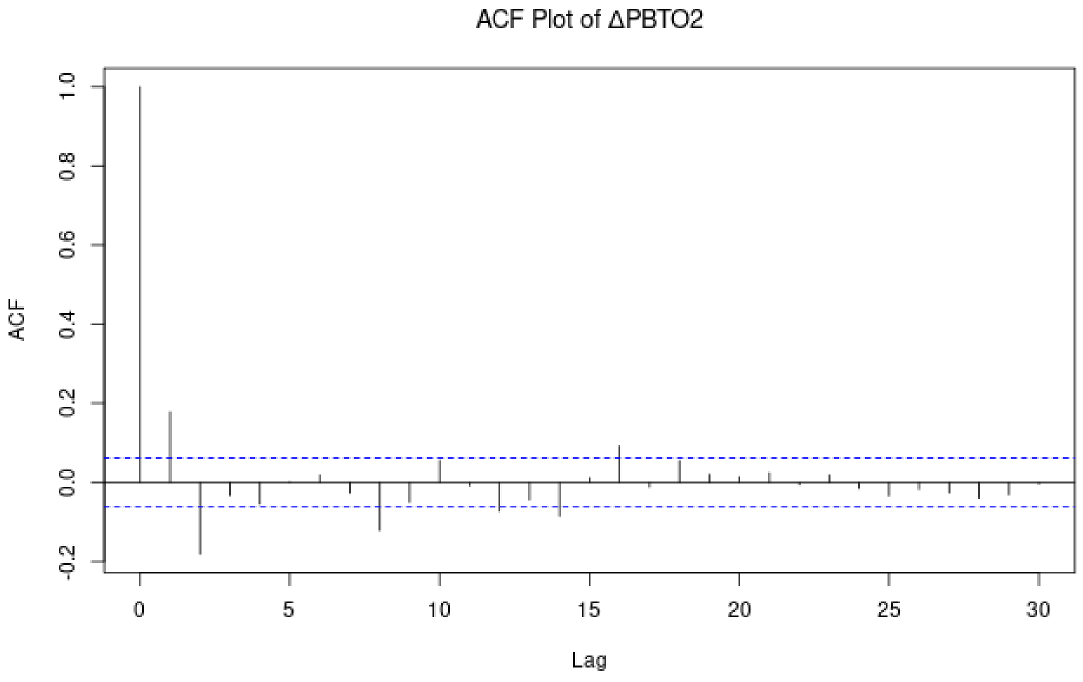

Supplementary File S1 Figure 6: ACF Plots of $PbtO_2$ (Top) and $\Delta PbtO_2$ (Bottom) for Subject 6

Subject 7

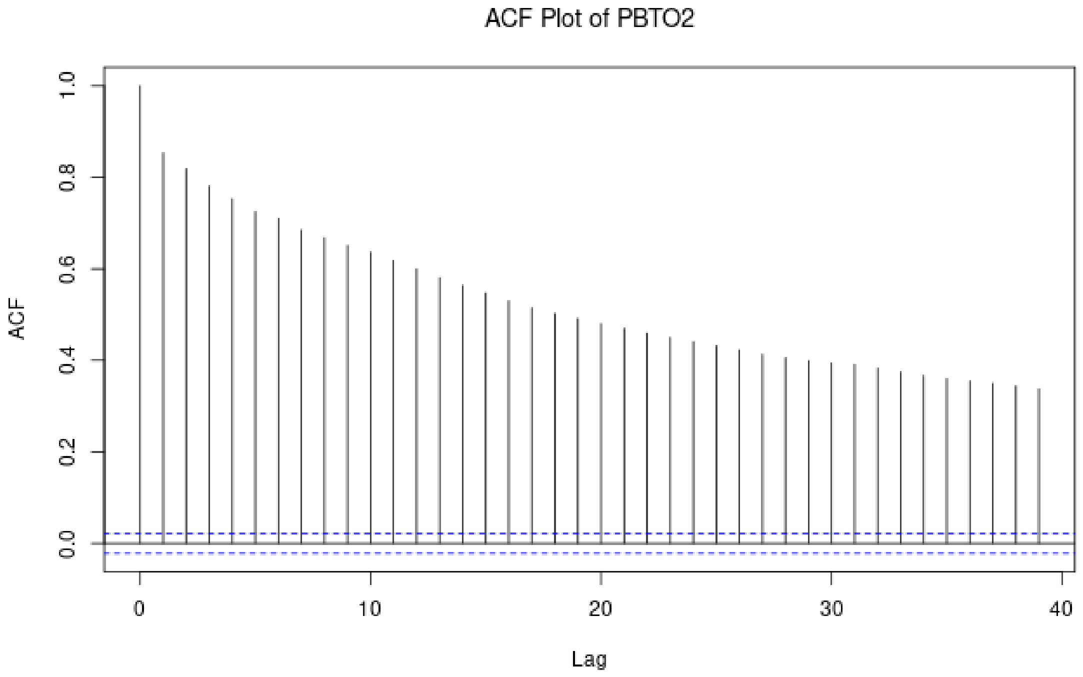

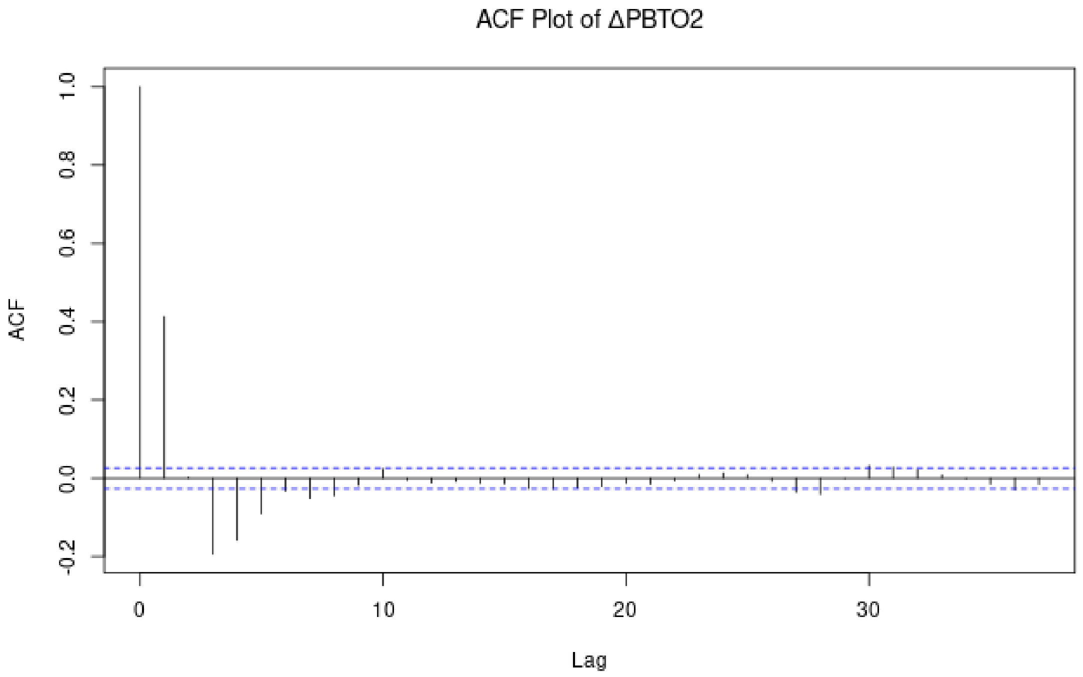

Supplementary File S1 Figure 7: ACF Plots of $PbtO_2$ (Top) and $\Delta PbtO_2$ (Bottom) for Subject 7

Subject 8

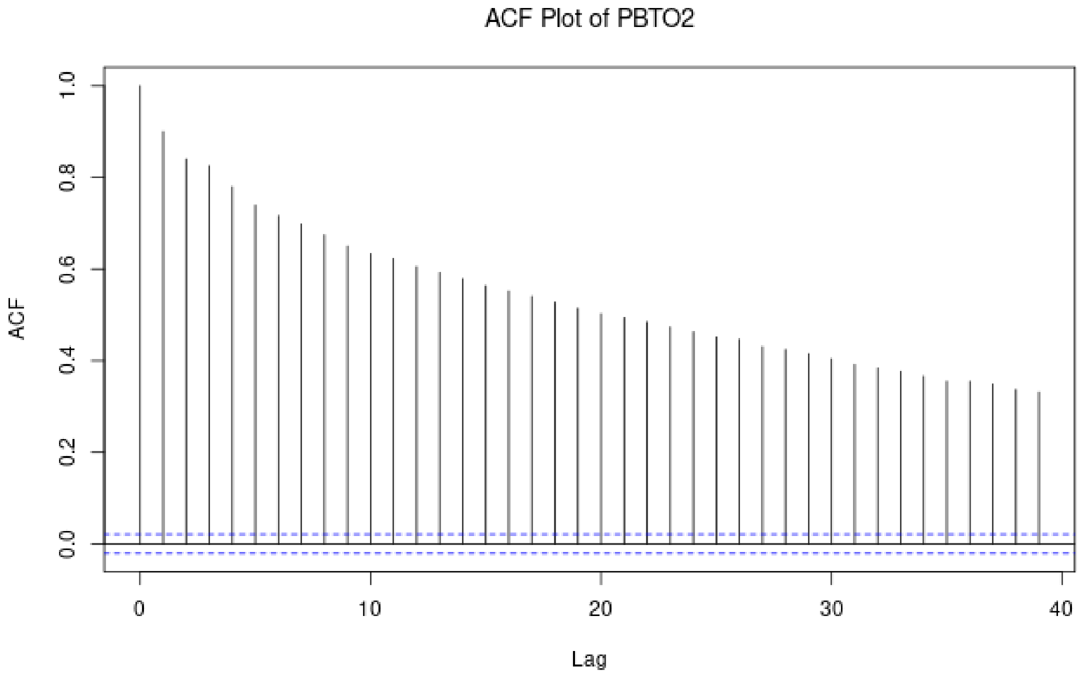

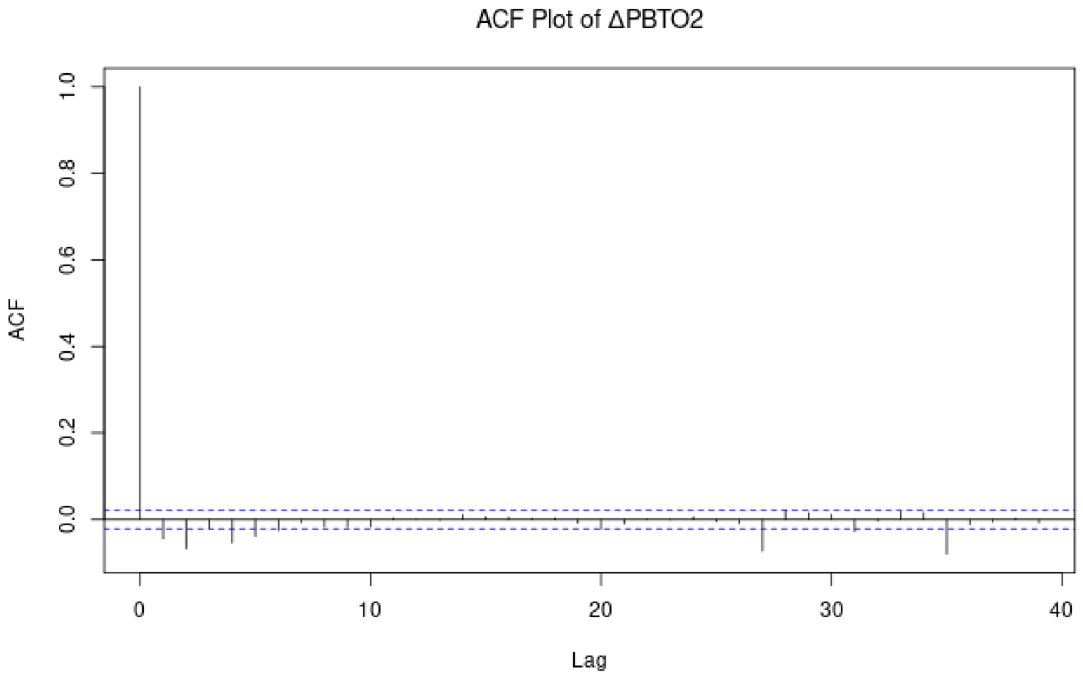

Supplementary File S1 Figure 8: ACF Plots of $PbtO_2$ (Top) and $\Delta PbtO_2$ (Bottom) for Subject 8

Subject 9

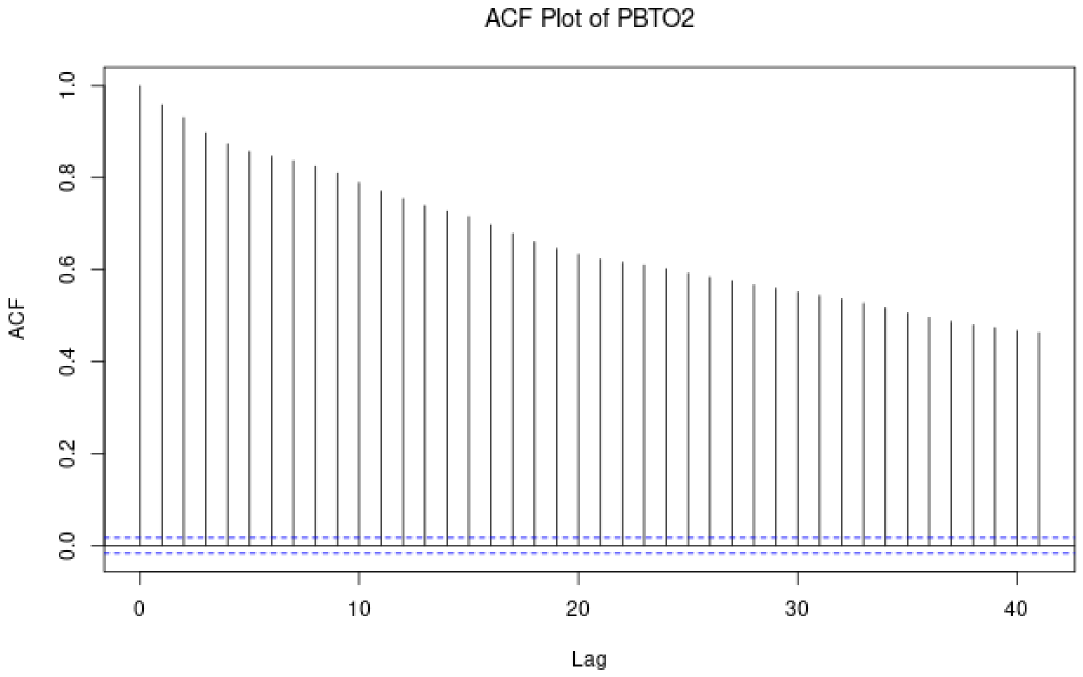

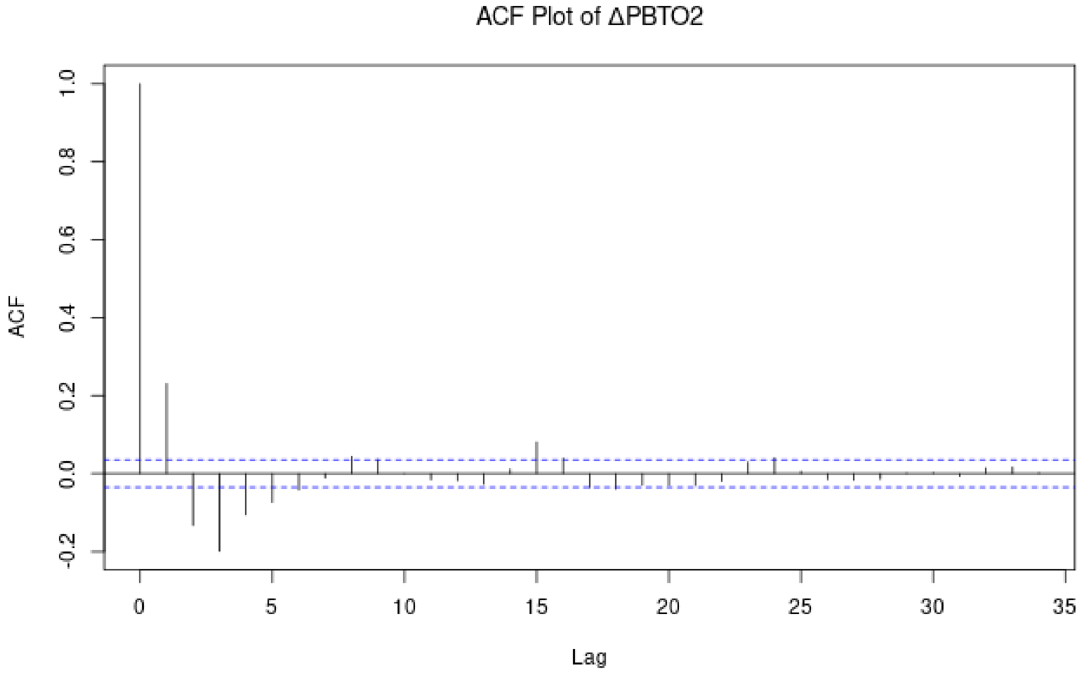

Supplementary File S1 Figure 9: ACF Plots of $PbtO_2$ (Top) and $\Delta PbtO_2$ (Bottom) for Subject 9

Subject 10

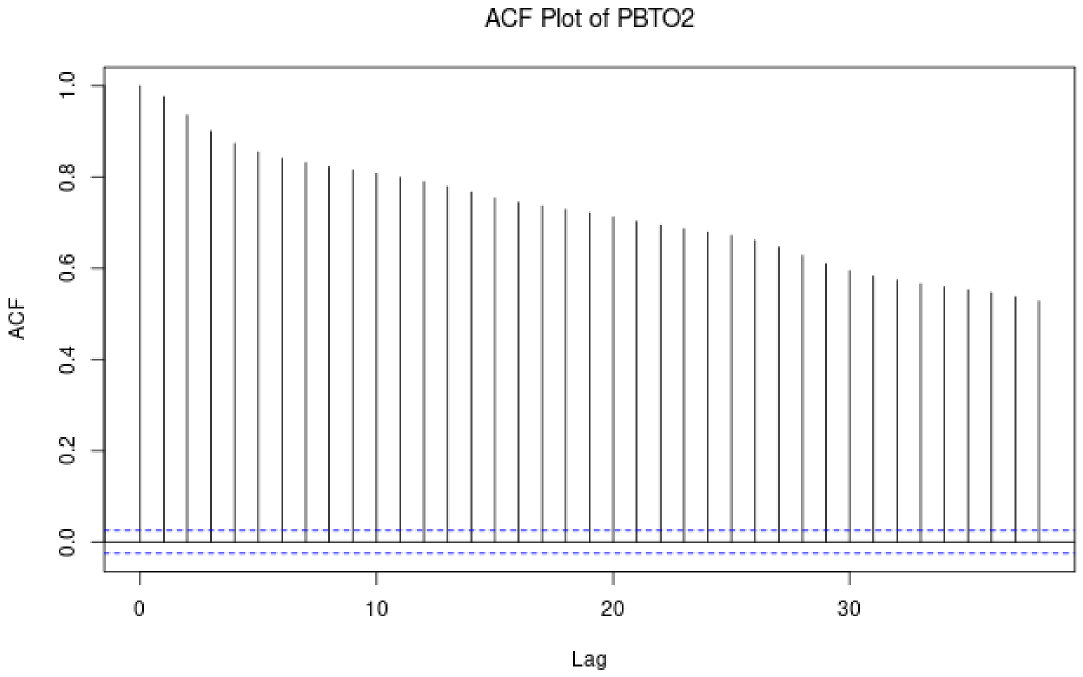

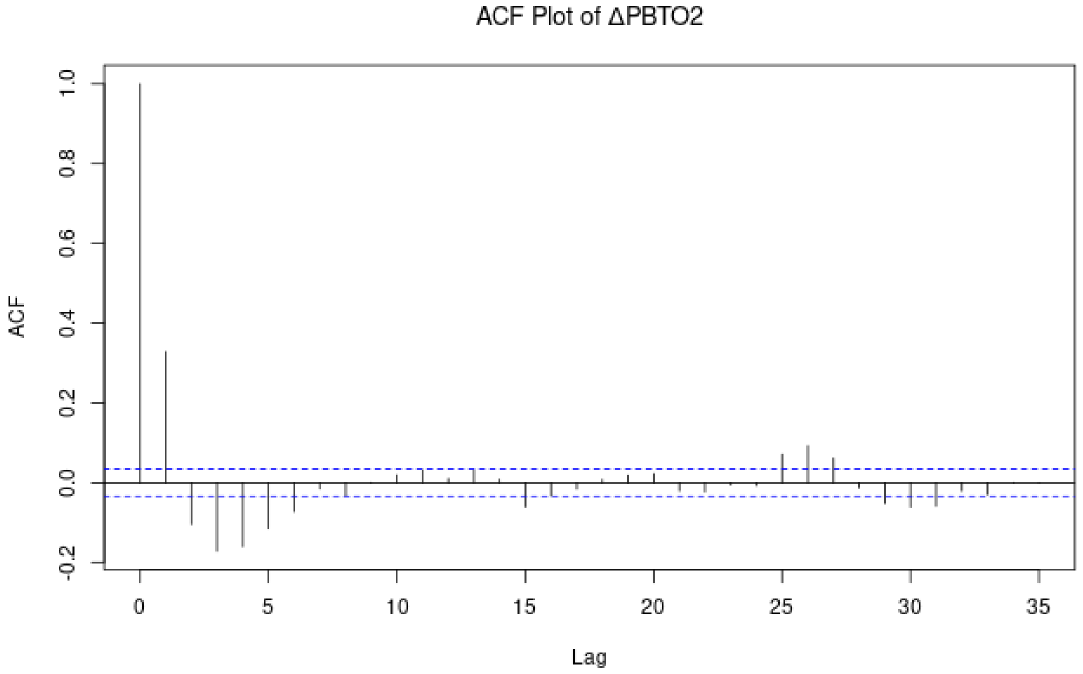

Supplementary File S1 Figure 10: ACF Plots of $PbtO_2$ (Top) and $\Delta PbtO_2$ (Bottom) for Subject 10

Subject 11

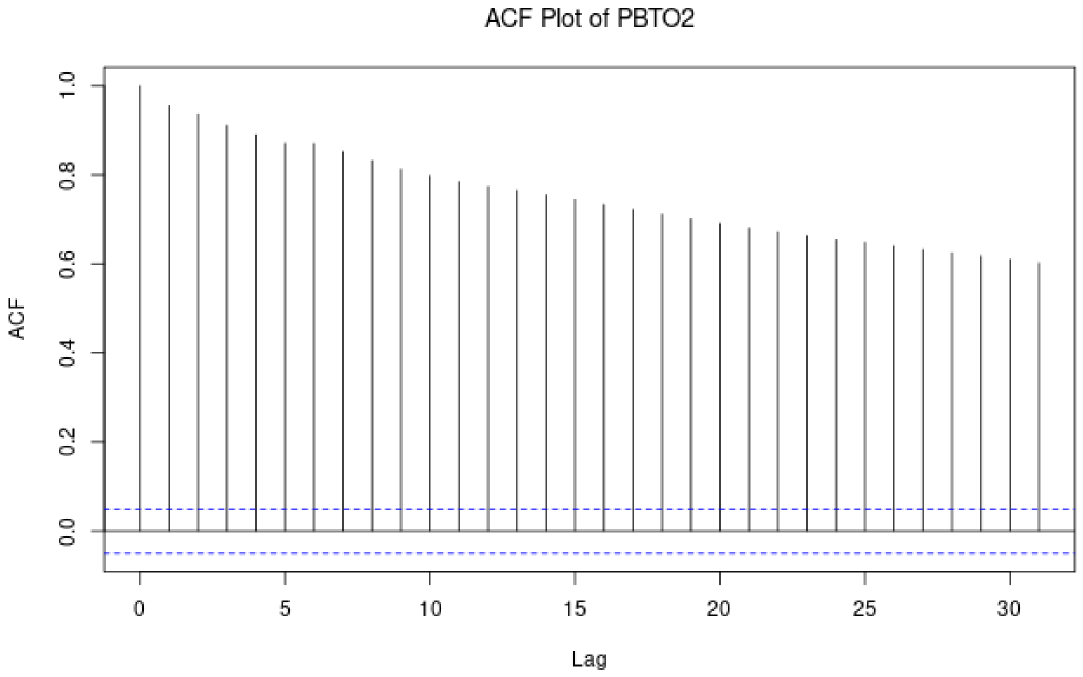

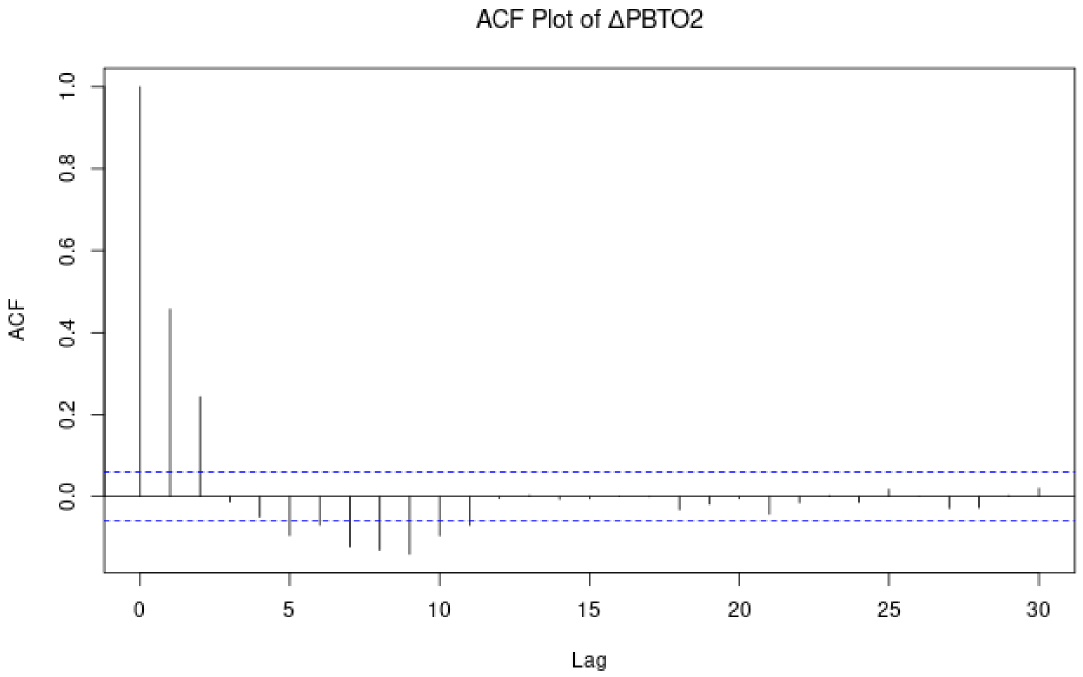

Supplementary File S1 Figure 11: ACF Plots of $PbtO_2$ (Top) and $\Delta PbtO_2$ (Bottom) for Subject 11

Subject 12

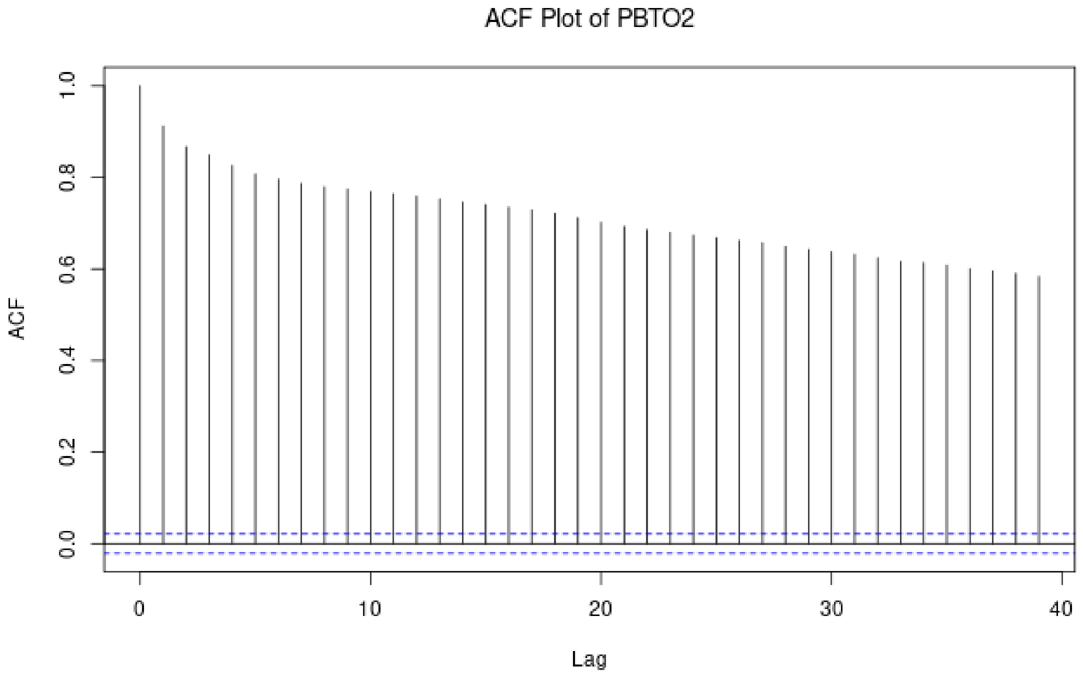

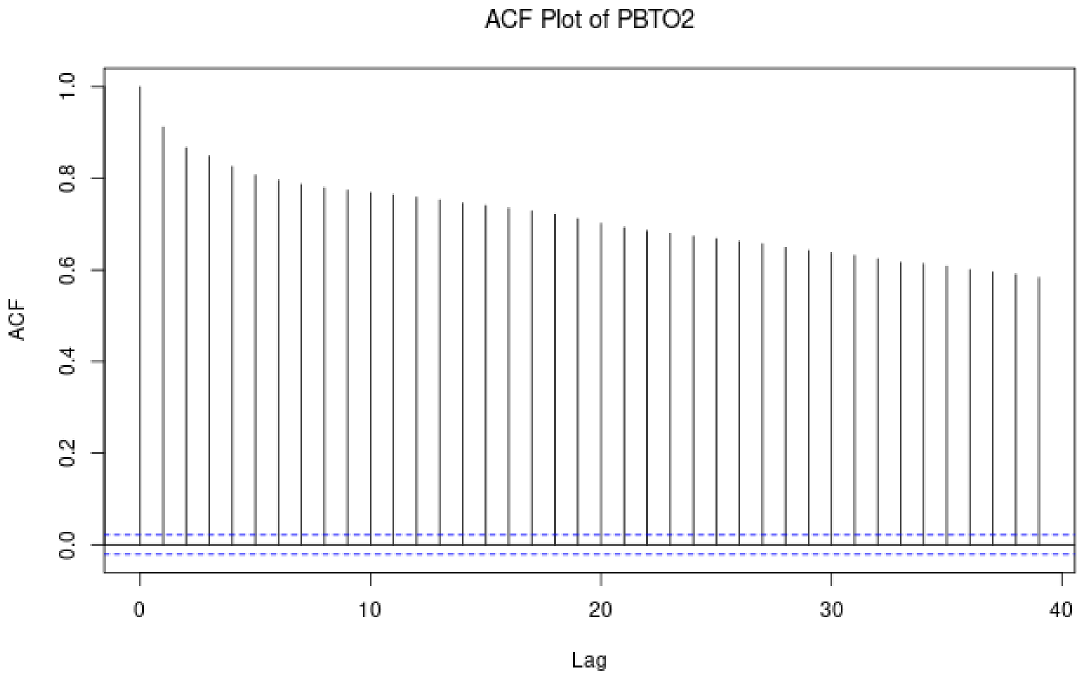

Supplementary File S1 Figure 12: ACF Plots of $PbtO_2$ (Top) and $\Delta PbtO_2$ (Bottom) for Subject 12

Subject 13

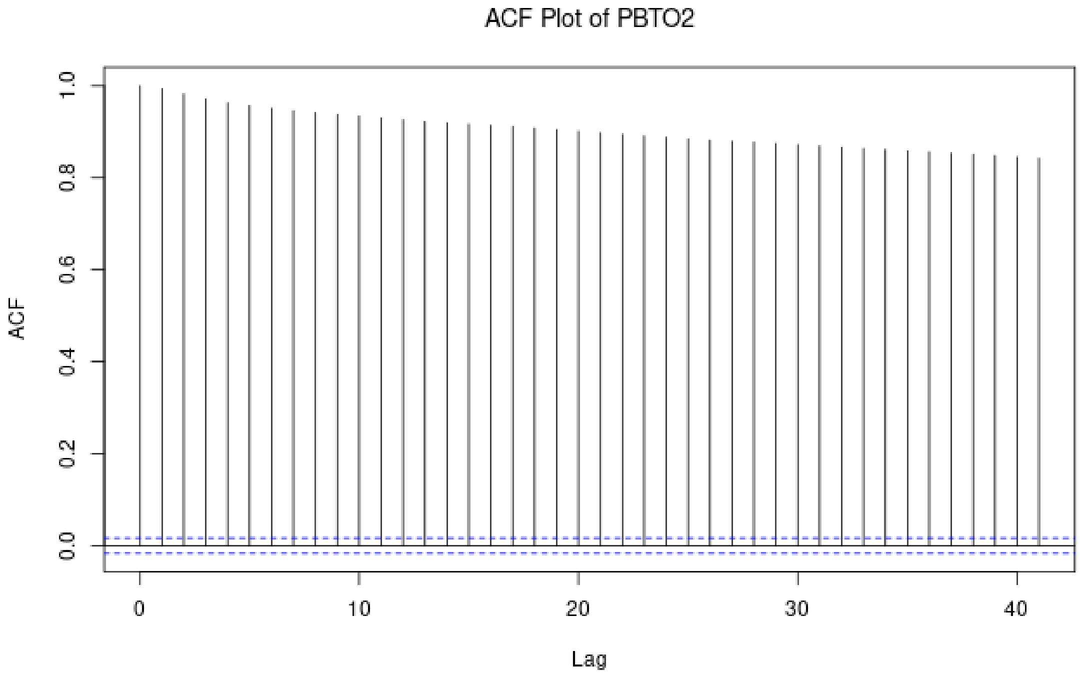

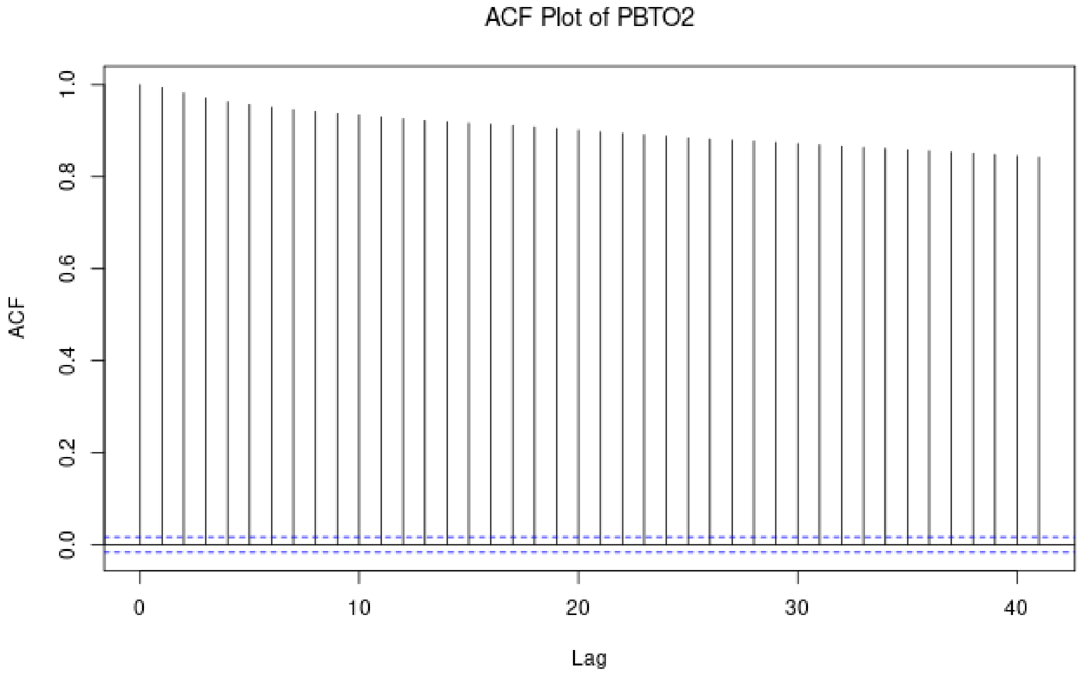

Supplementary File S1 Figure 13: ACF Plots of $PbtO_2$ (Top) and $\Delta PbtO_2$ (Bottom) for Subject 13

Subject 14

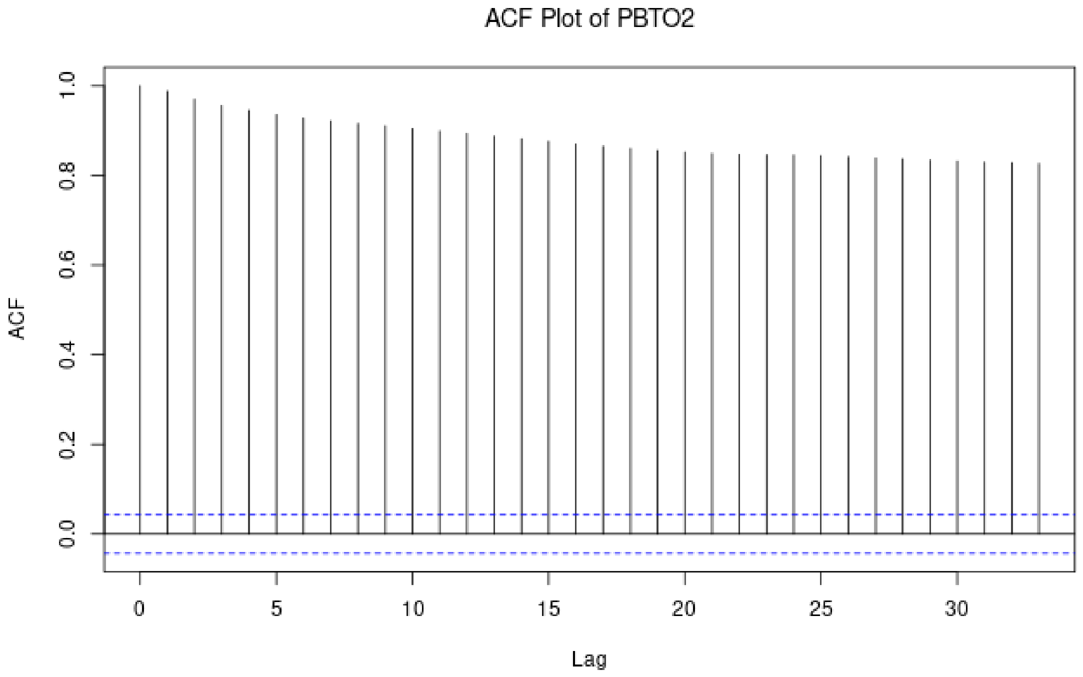

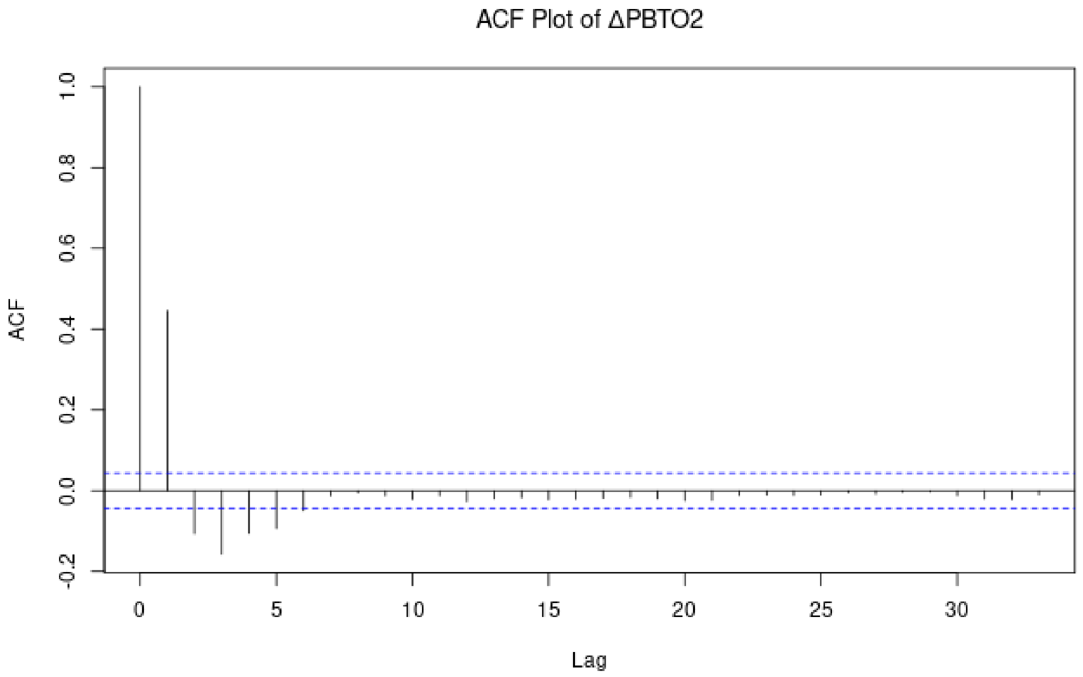

Supplementary File S1 Figure 14: ACF Plots of $PbtO_2$ (Top) and $\Delta PbtO_2$ (Bottom) for Subject 14

Subject 15

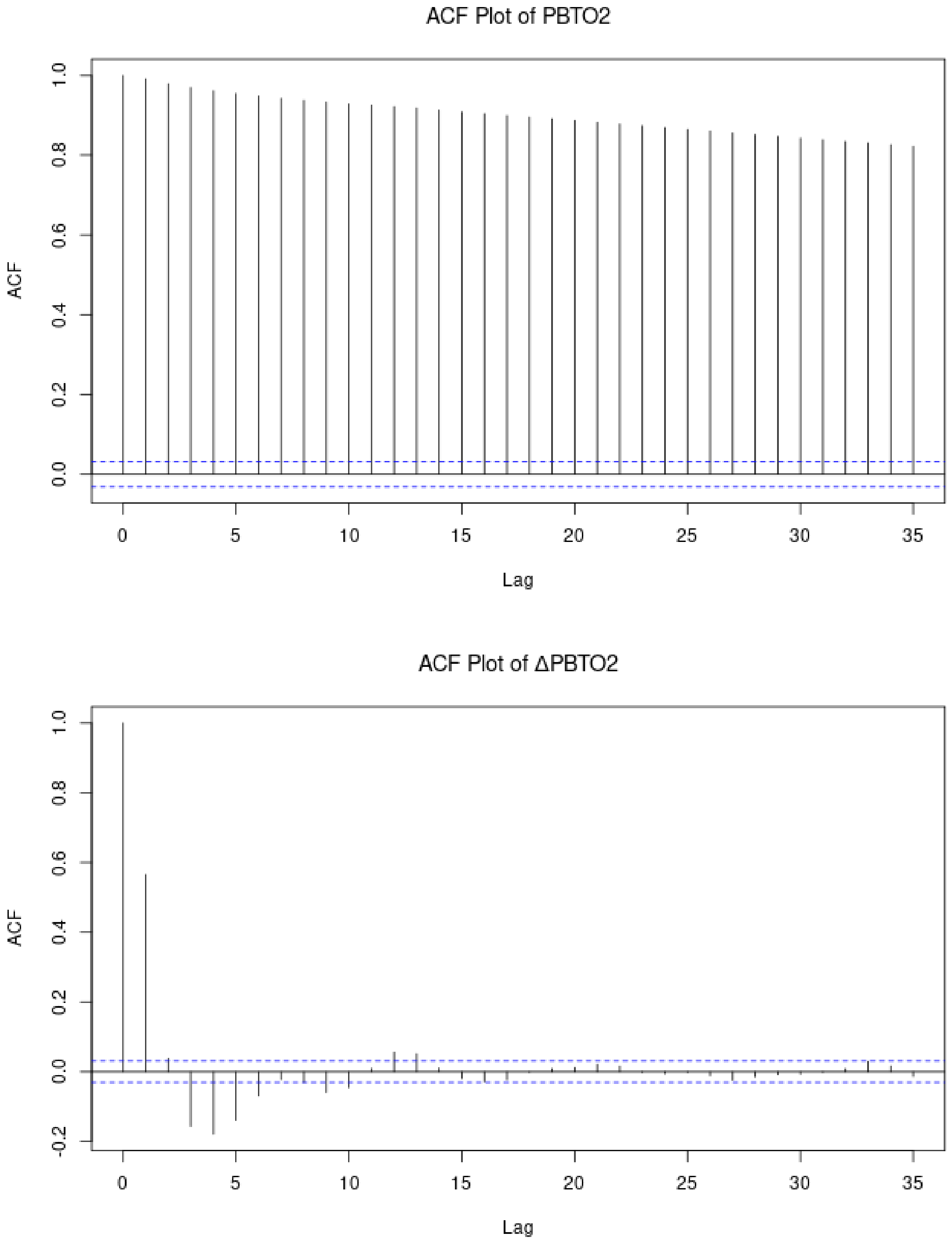

Supplementary File S1 Figure 15: ACF Plots of $PbtO_2$ (Top) and $\Delta PbtO_2$ (Bottom) for Subject 15

Subject 16

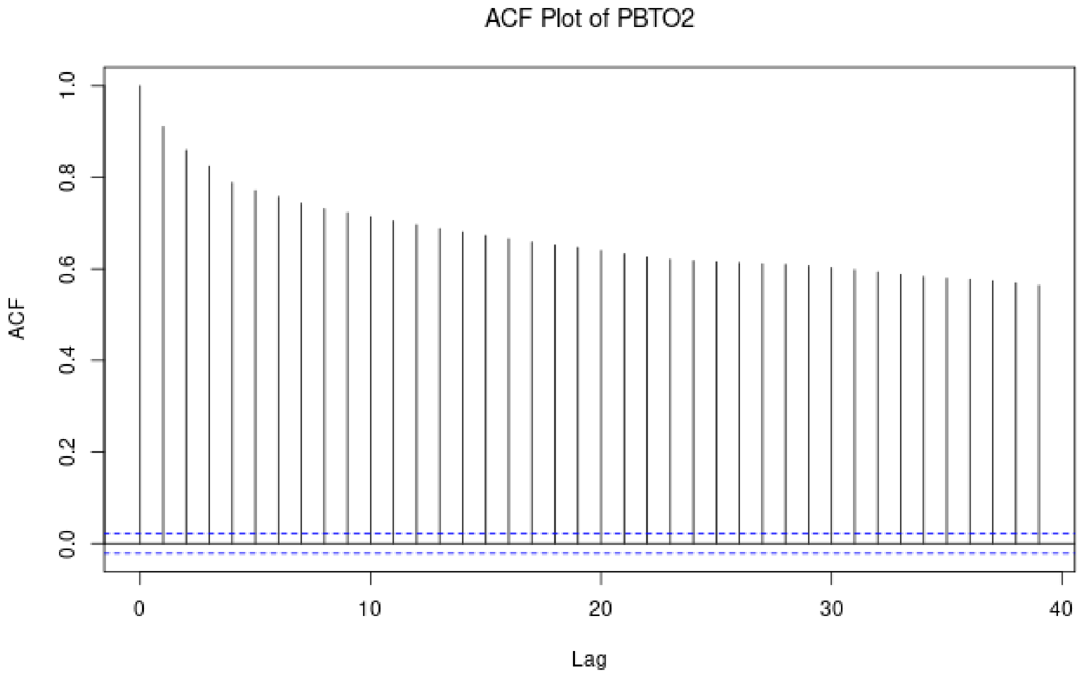

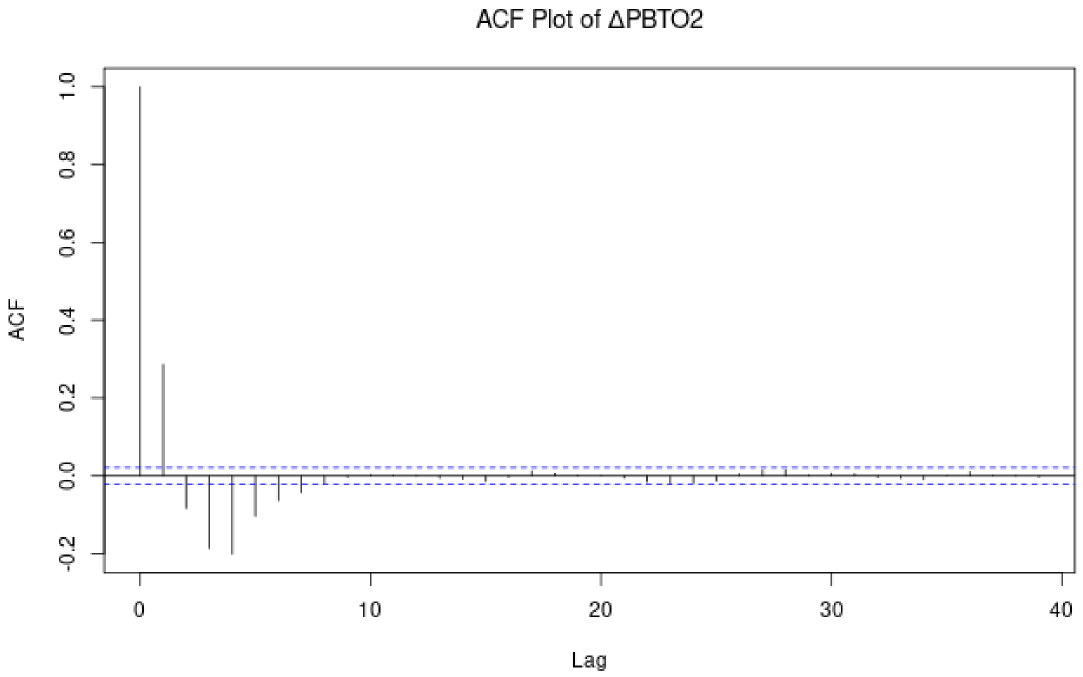

Supplementary File S1 Figure 16: ACF Plots of $PbtO_2$ (Top) and $\Delta PbtO_2$ (Bottom) for Subject 16

Subject 17

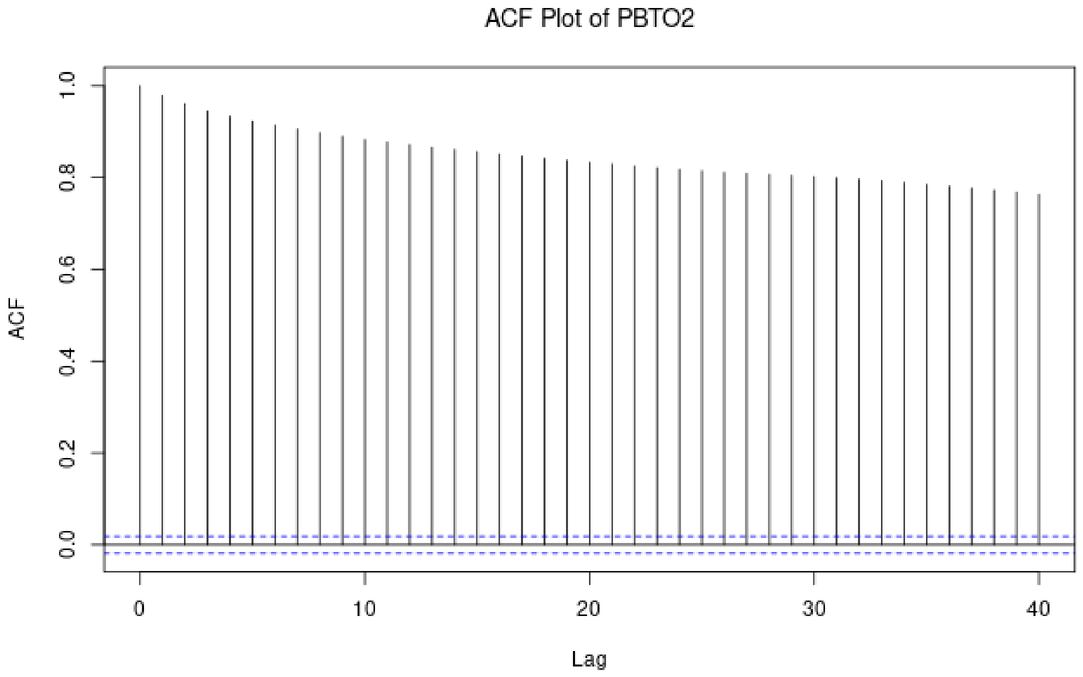

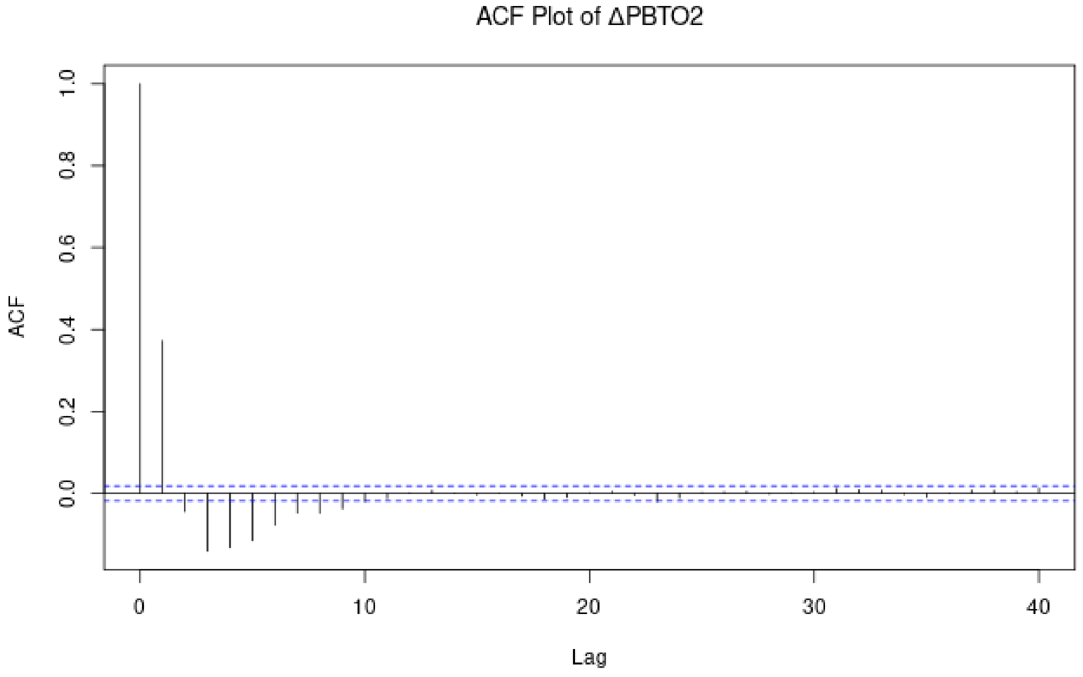

Supplementary File S1 Figure 17: ACF Plots of $PbtO_2$ (Top) and $\Delta PbtO_2$ (Bottom) for Subject 17

Subject 18

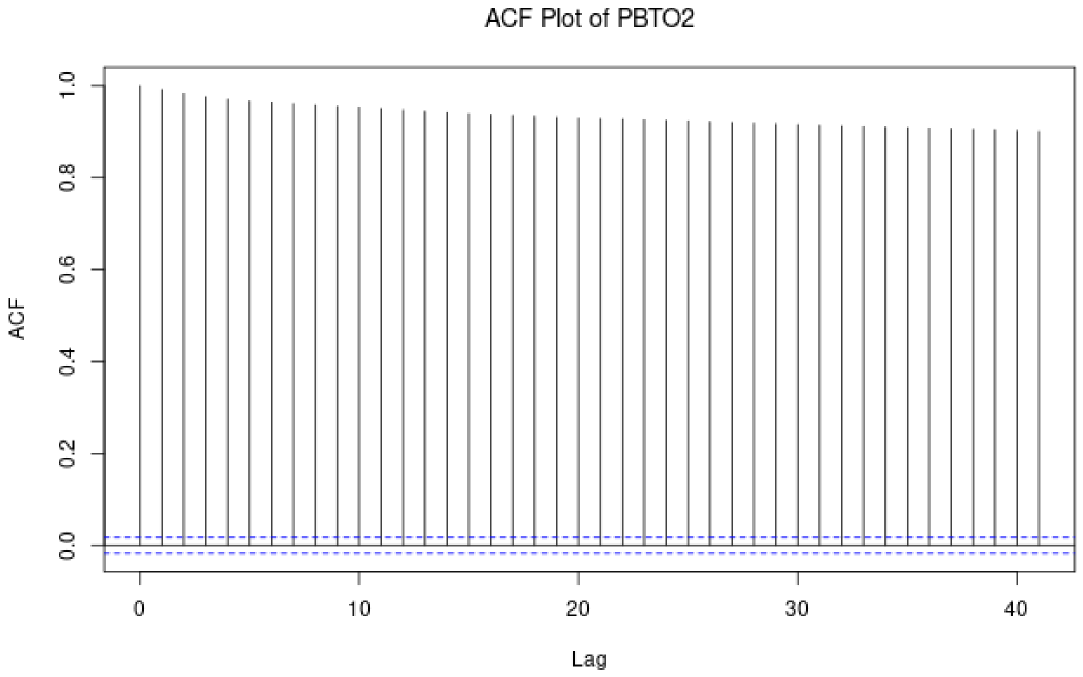

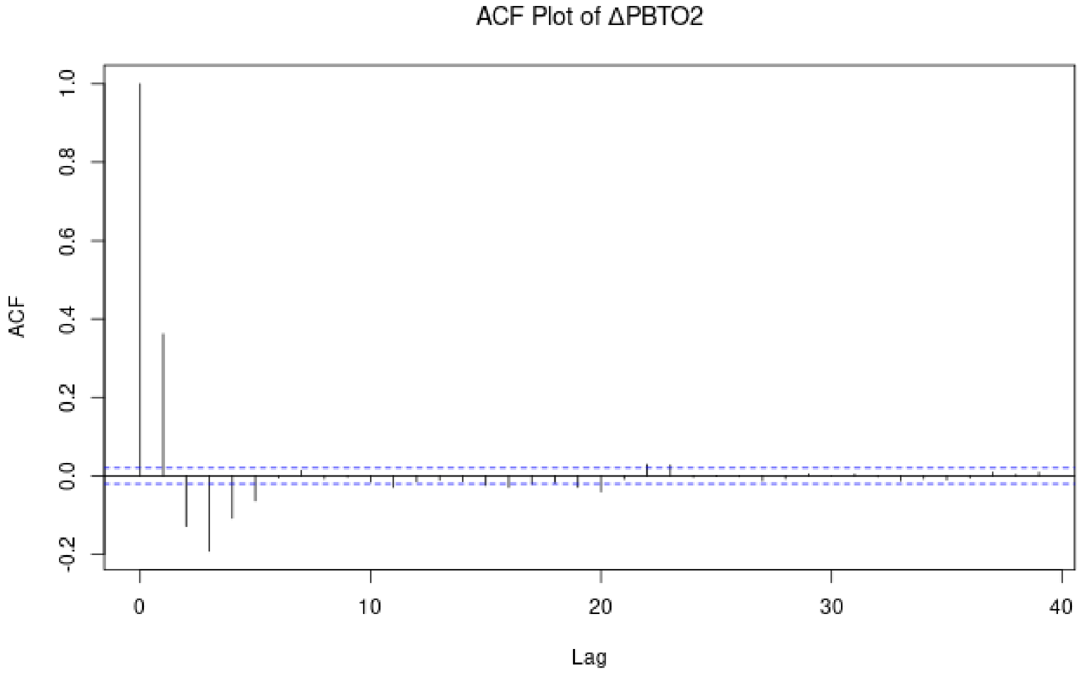

Supplementary File S1 Figure 18: ACF Plots of $PbtO_2$ (Top) and $\Delta PbtO_2$ (Bottom) for Subject 18

Subject 19

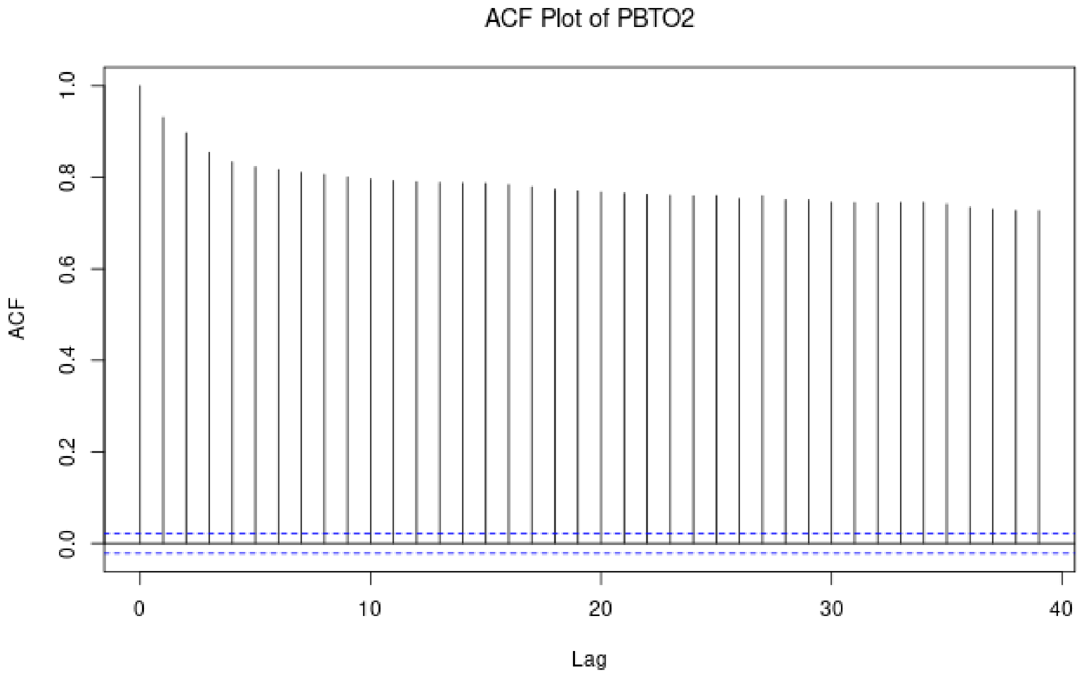

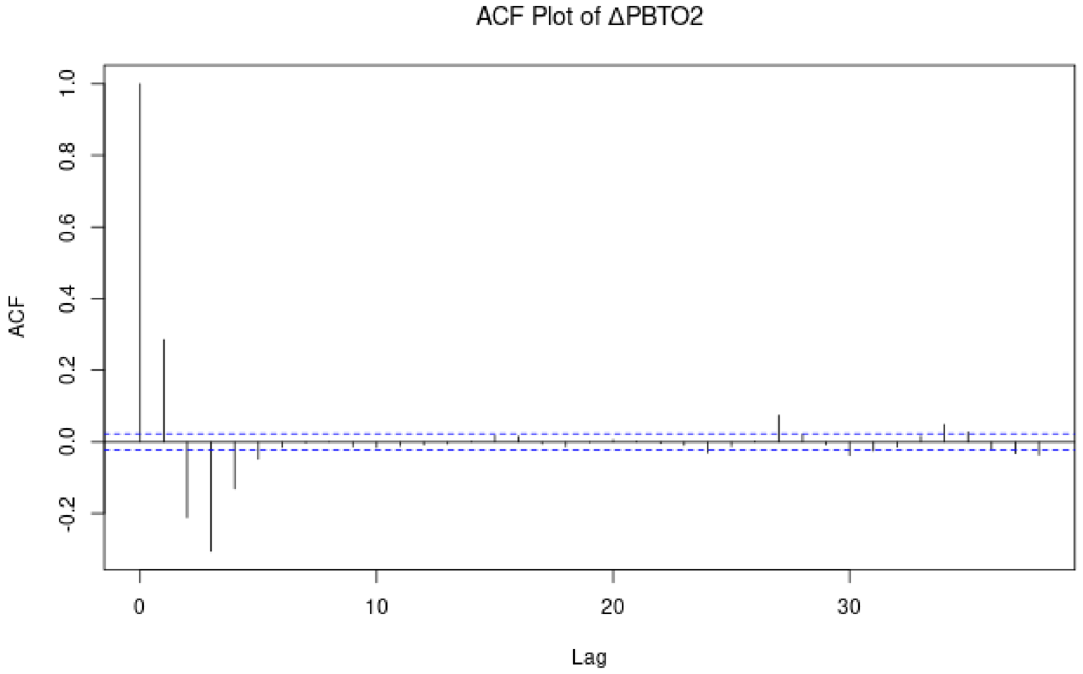

Supplementary File S1 Figure 19: ACF Plots of $PbtO_2$ (Top) and $\Delta PbtO_2$ (Bottom) for Subject 19

Subject 20

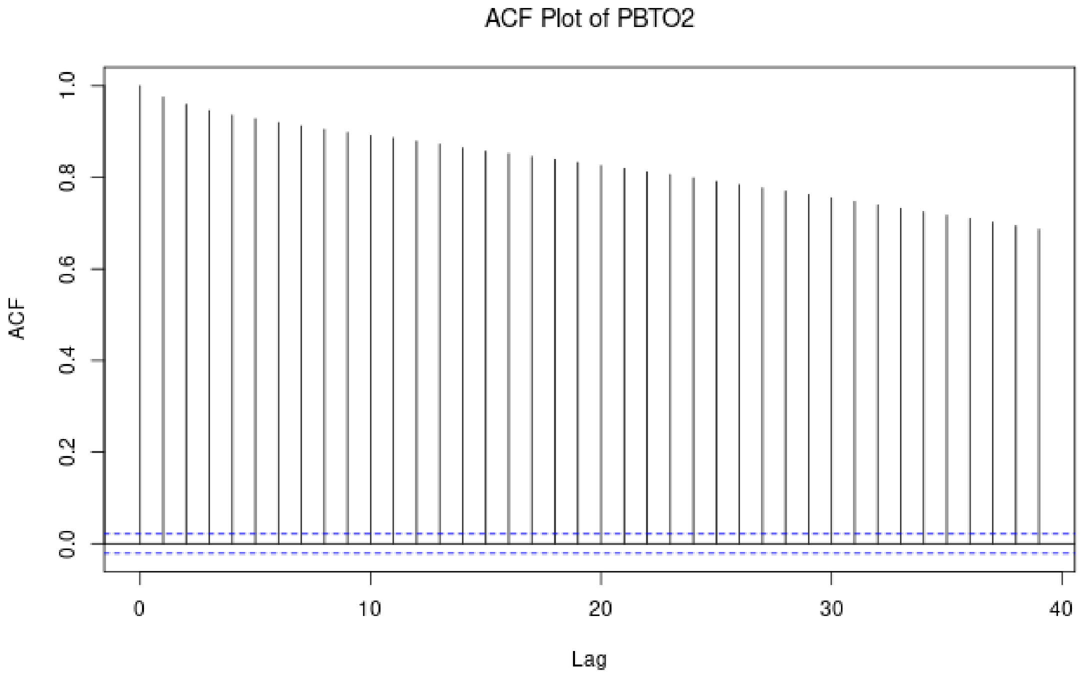

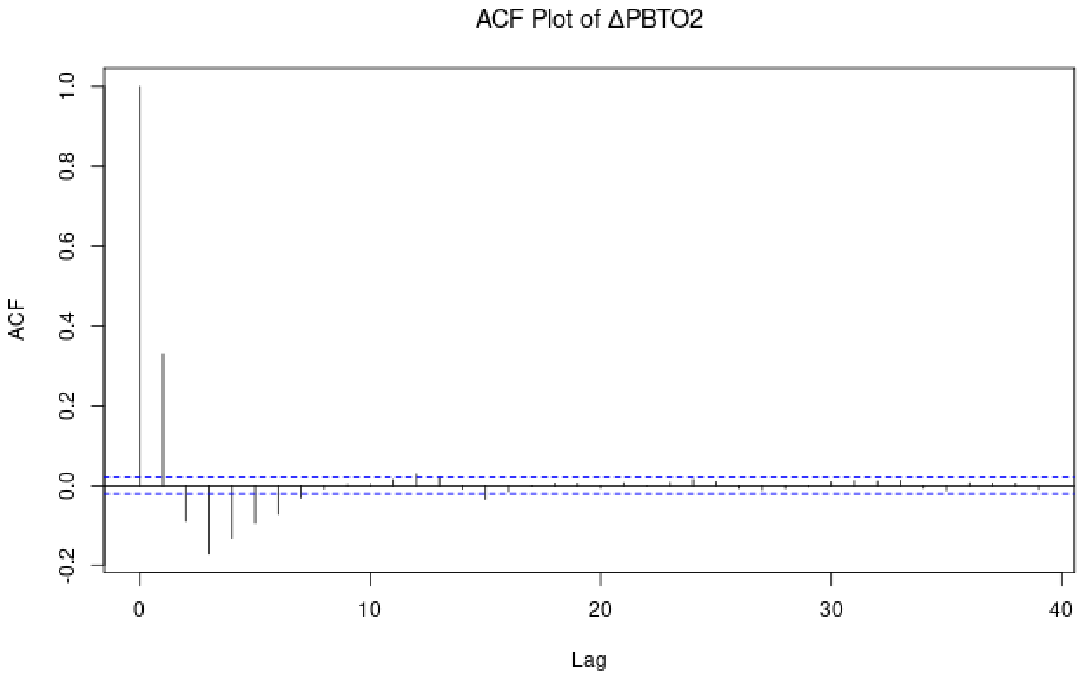

Supplementary File S1 Figure 20: ACF Plots of $PbtO_2$ (Top) and $\Delta PbtO_2$ (Bottom) for Subject 20

2 Supplementary File S2: Evaluation of residuals of the linear ΔPbtO2 ~ ΔrSO2Lag1 models to ensure autocorrelative structure was accounted for

Plot Legend: *For the following plots the dashed blue line represents the significance levels which were set to a correlation level of +/-(2/$N^{1/2}$), where N is the number of samples.* *ACF = Autocorrelative function, PACF = Partial Autocorrelative function, ΔPbtO2 = change in brain tissue oxygen tension, ΔrSO_2Lag1 = he one-minute lagged change in regional cerebral oxygen saturation.*

Subject 1

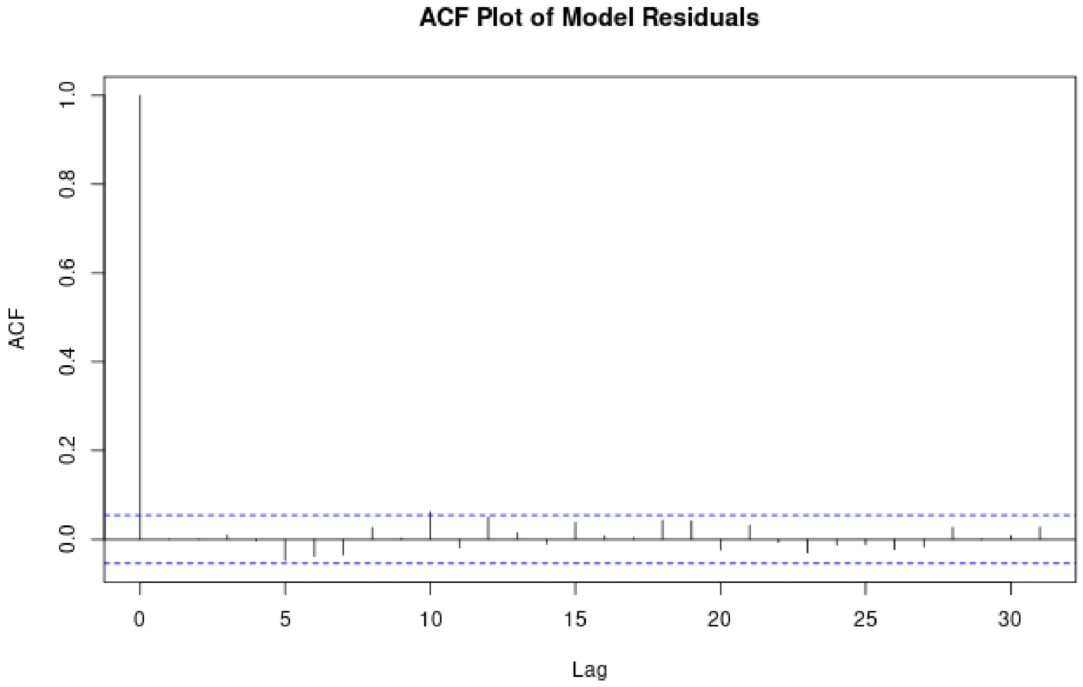

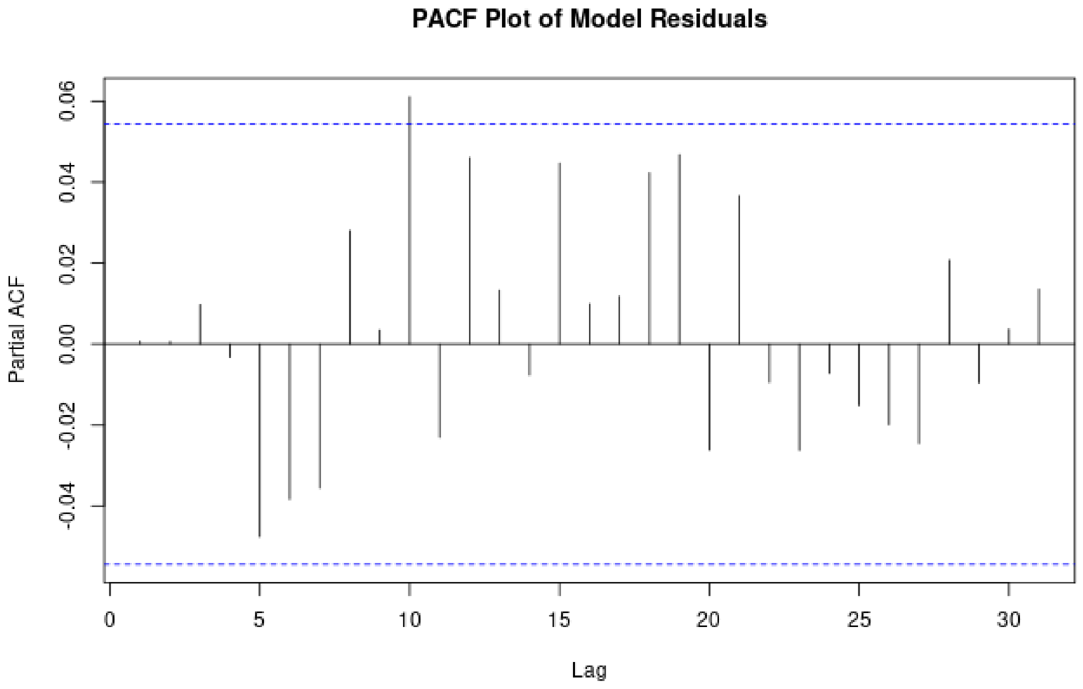

Supplementary File S2 Figure 1: Evaluation residuals of the linear ΔPbtO2 ~ ΔrSO2Lag1 models to ensure autocorrelative structure was accounted for. ACF (top) and PACF (bottom) plots of residuals of the model for Subject 1

Subject 2

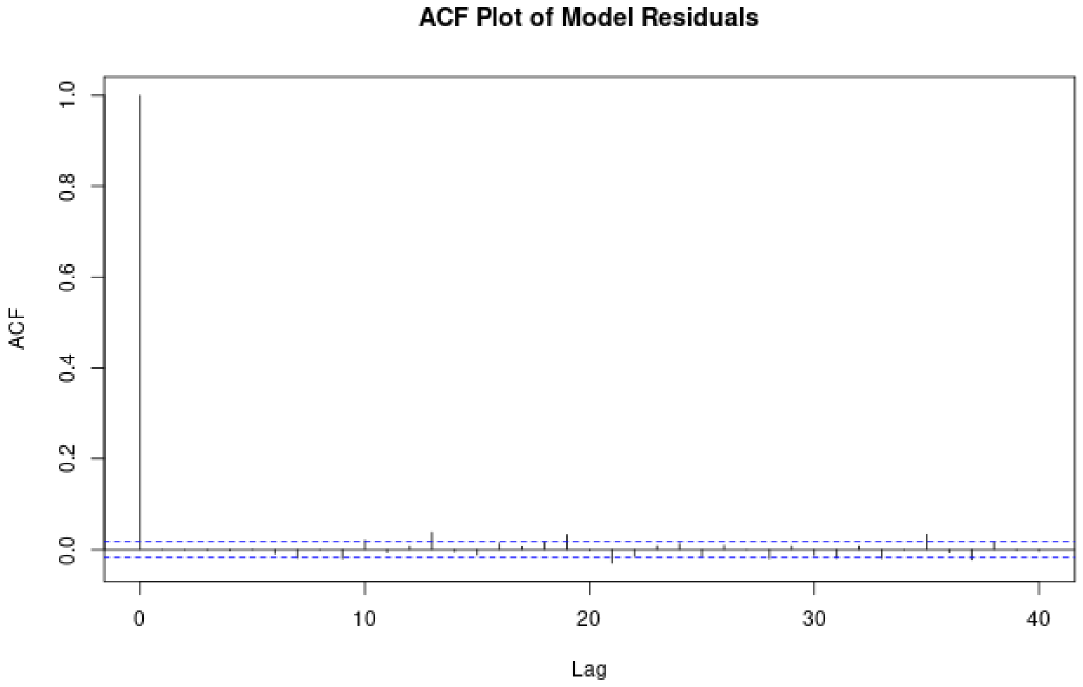

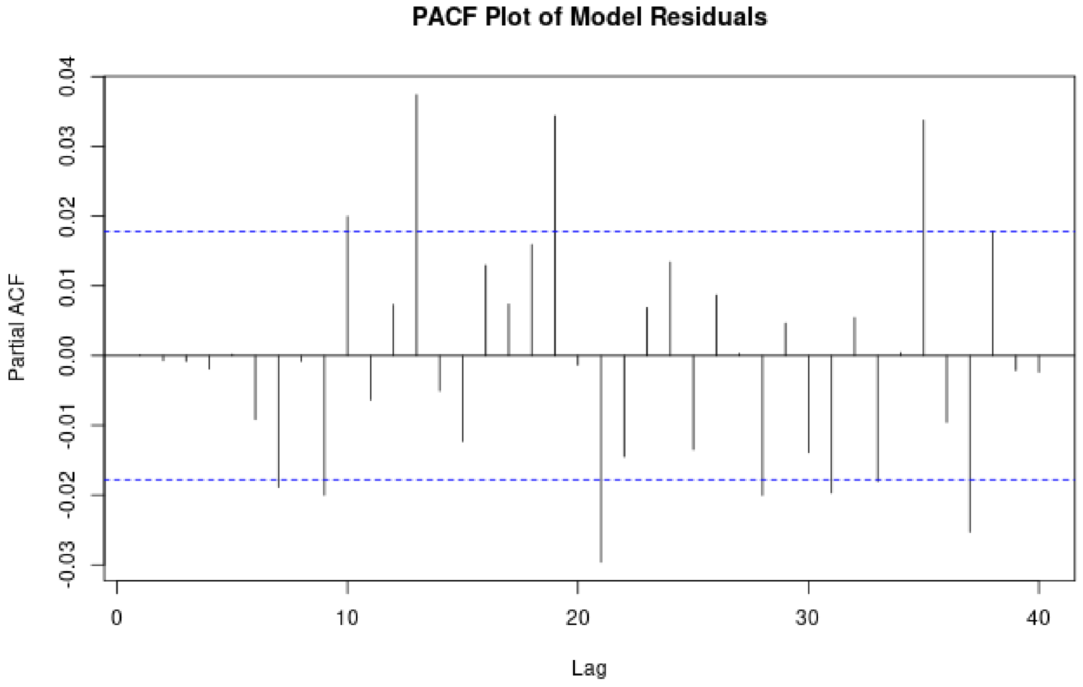

Supplementary File S2 Figure 2: Evaluation residuals of the linear ΔPbtO2 ~ ΔrSO2Lag1 models to ensure autocorrelative structure was accounted for. ACF (top) and PACF (bottom) plots of residuals of the model for Subject 2

Subject 3

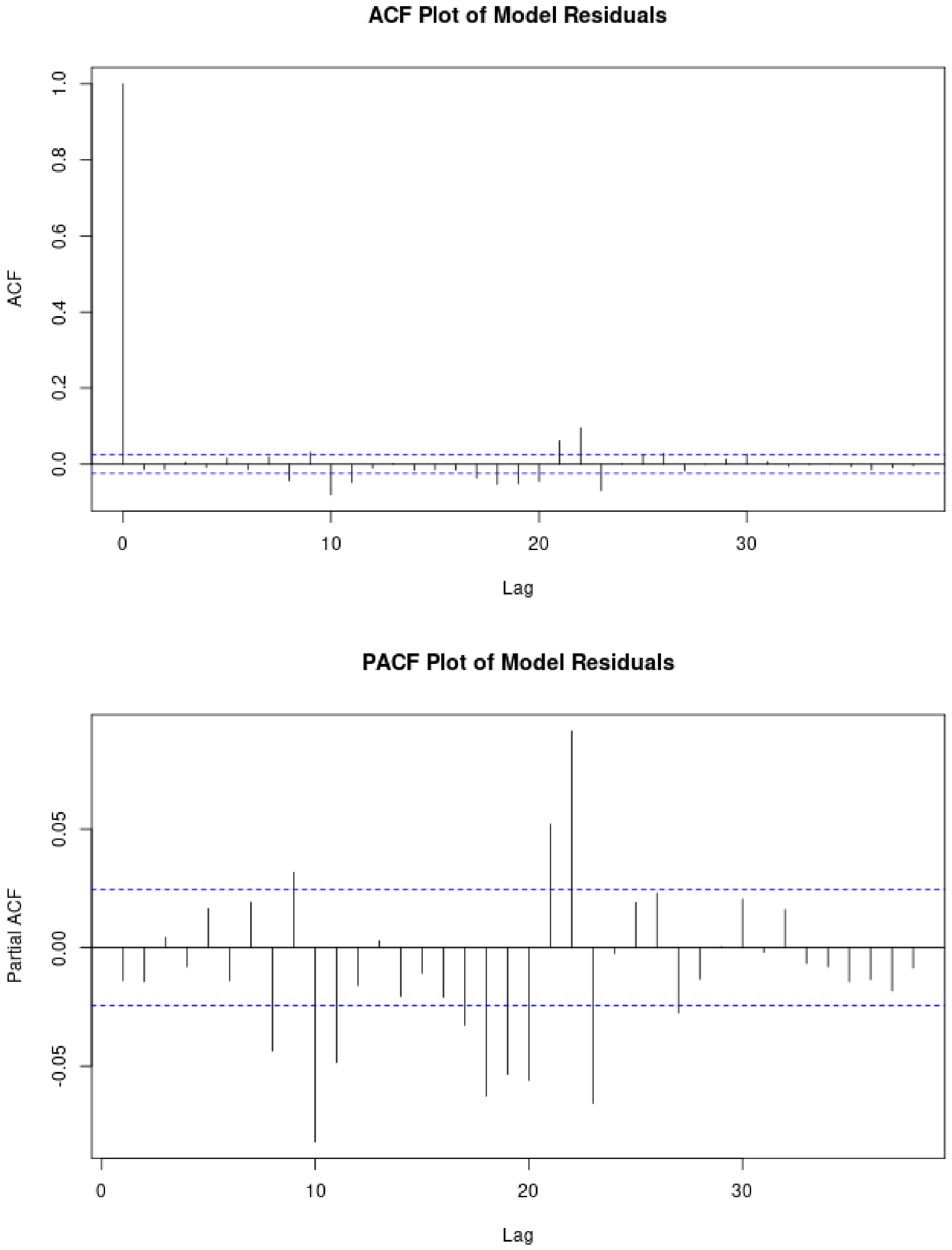

Supplementary File S2 Figure 3: Evaluation residuals of the linear ΔPbtO2 ~ ΔrSO2Lag1 models to ensure autocorrelative structure was accounted for. ACF (top) and PACF (bottom) plots of residuals of the model for Subject 3

Subject 4

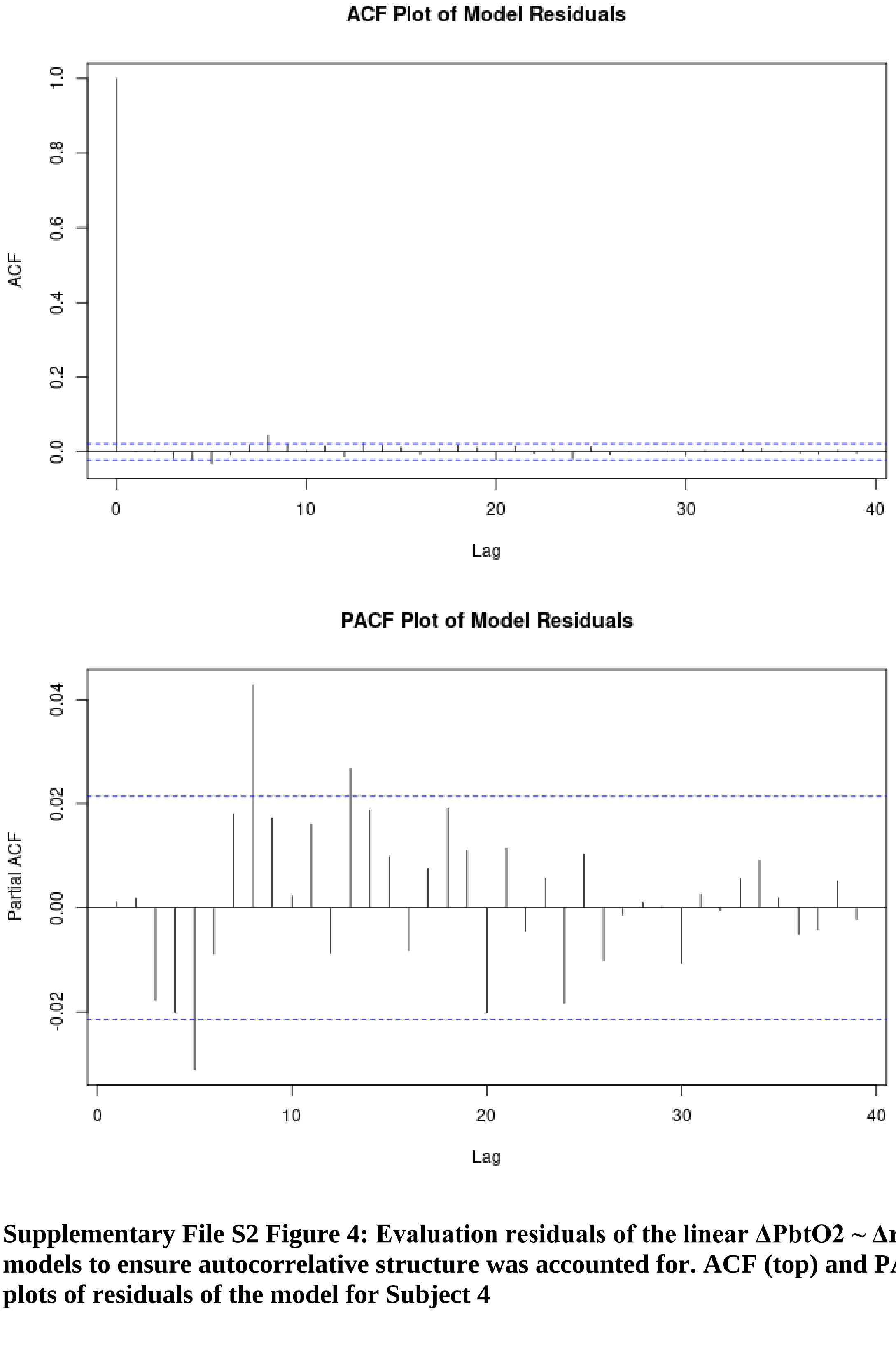

Supplementary File S2 Figure 4: Evaluation residuals of the linear ΔPbtO2 ~ ΔrSO2Lag1 models to ensure autocorrelative structure was accounted for. ACF (top) and PACF (bottom) plots of residuals of the model for Subject 4

Subject 5

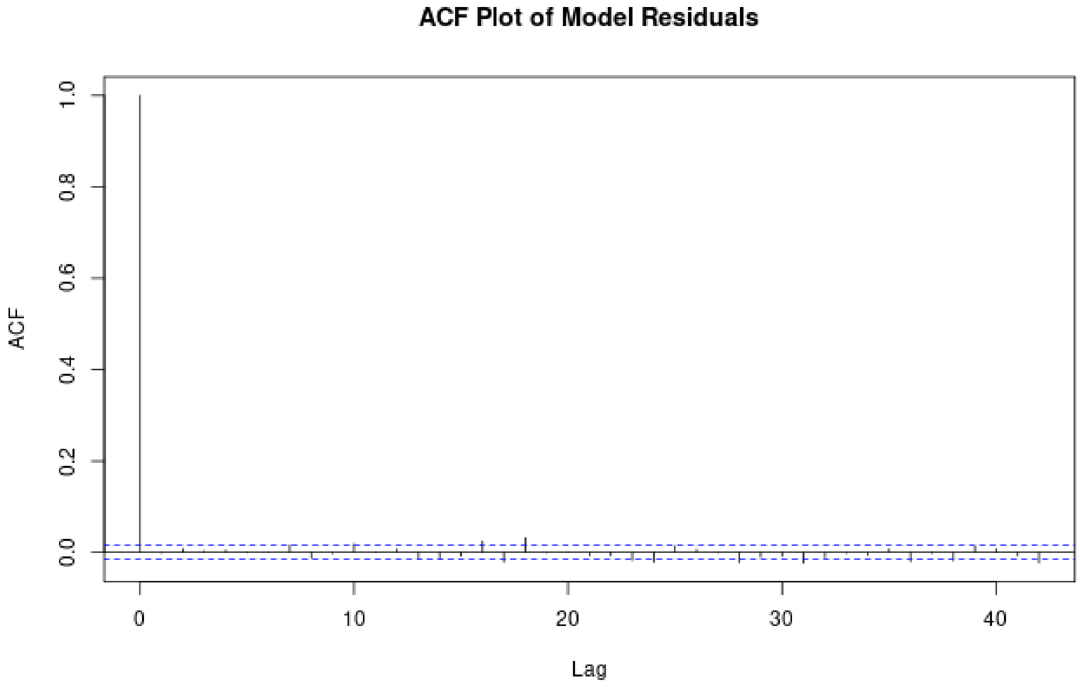

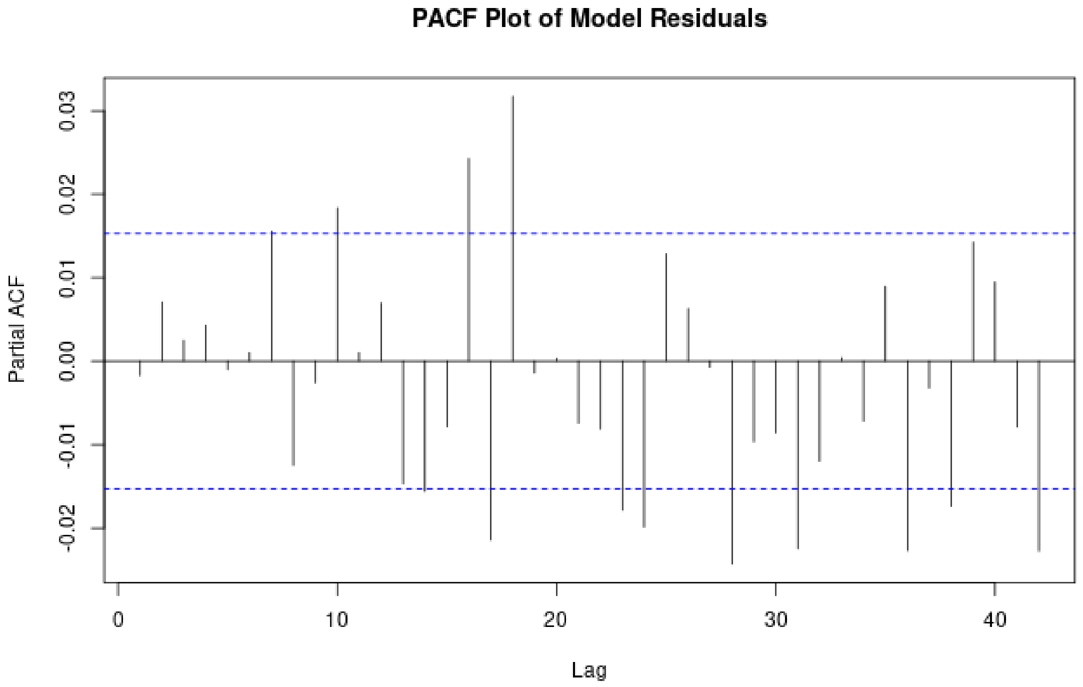

Supplementary File S2 Figure 5: Evaluation residuals of the linear ΔPbtO2 ~ ΔrSO2Lag1 models to ensure autocorrelative structure was accounted for. ACF (top) and PACF (bottom) plots of residuals of the model for Subject 5

Subject 6

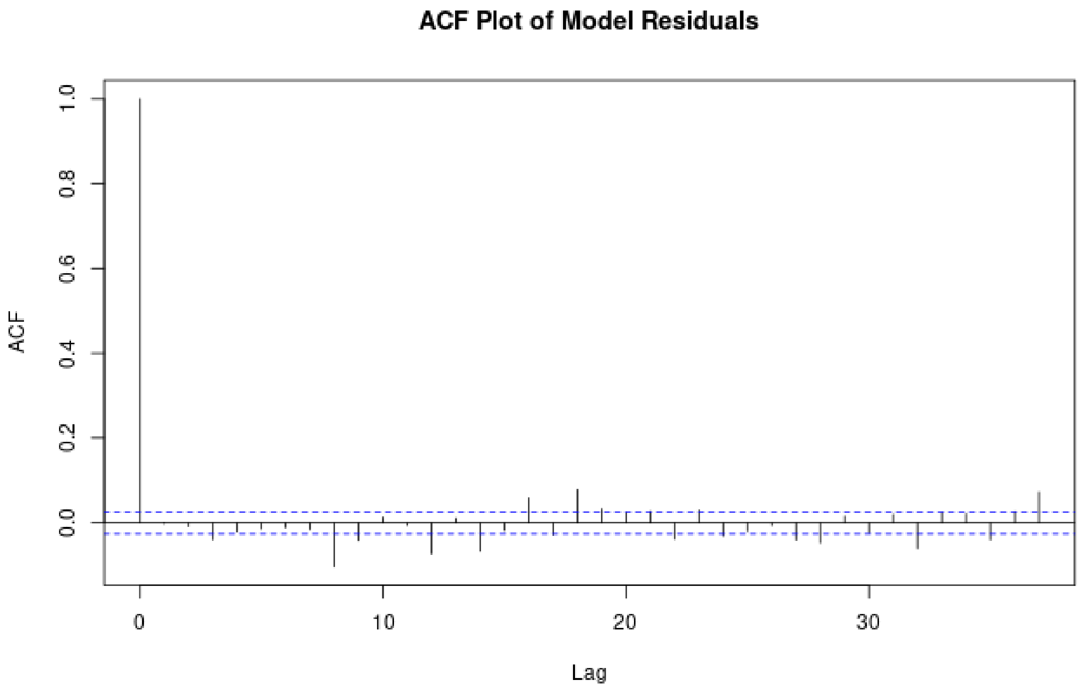

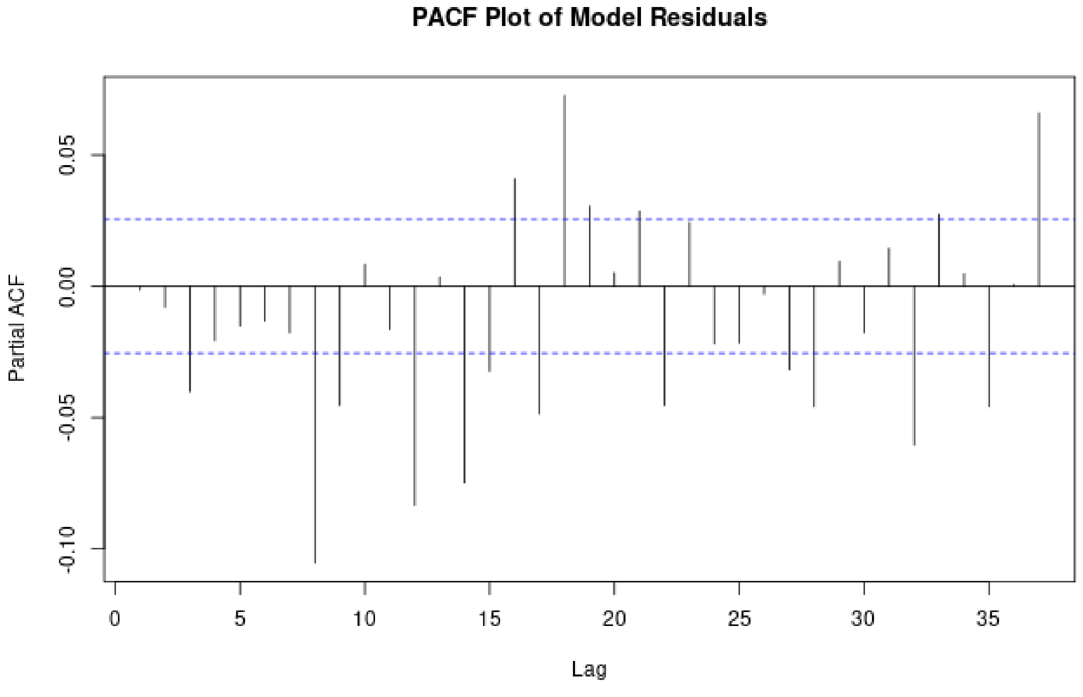

Supplementary File S2 Figure 6: Evaluation residuals of the linear ΔPbtO2 ~ ΔrSO2Lag1 models to ensure autocorrelative structure was accounted for. ACF (top) and PACF (bottom) plots of residuals of the model for Subject 6

Subject 7

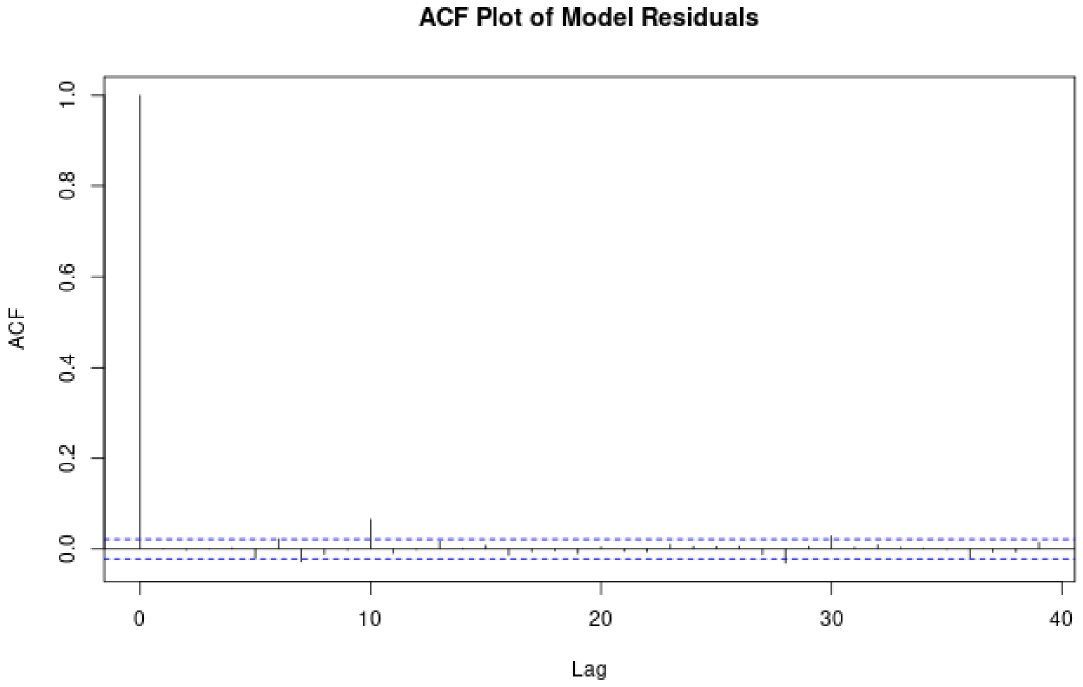

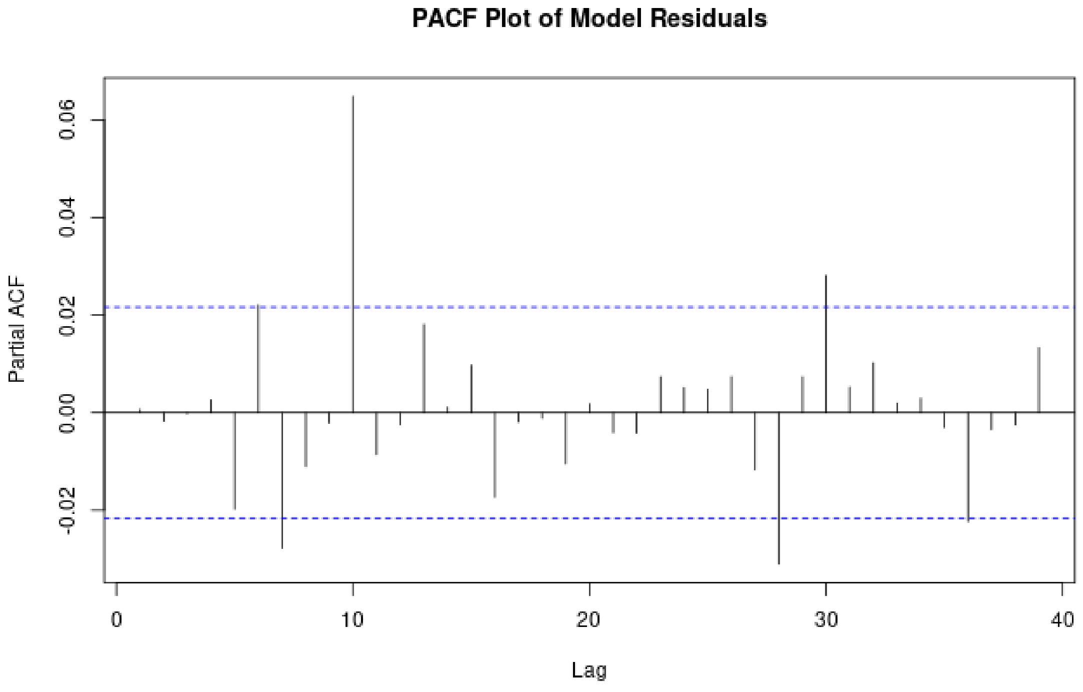

Supplementary File S2 Figure 7: Evaluation residuals of the linear ΔPbtO2 ~ ΔrSO2Lag1 models to ensure autocorrelative structure was accounted for. ACF (top) and PACF (bottom) plots of residuals of the model for Subject 7

Subject 8

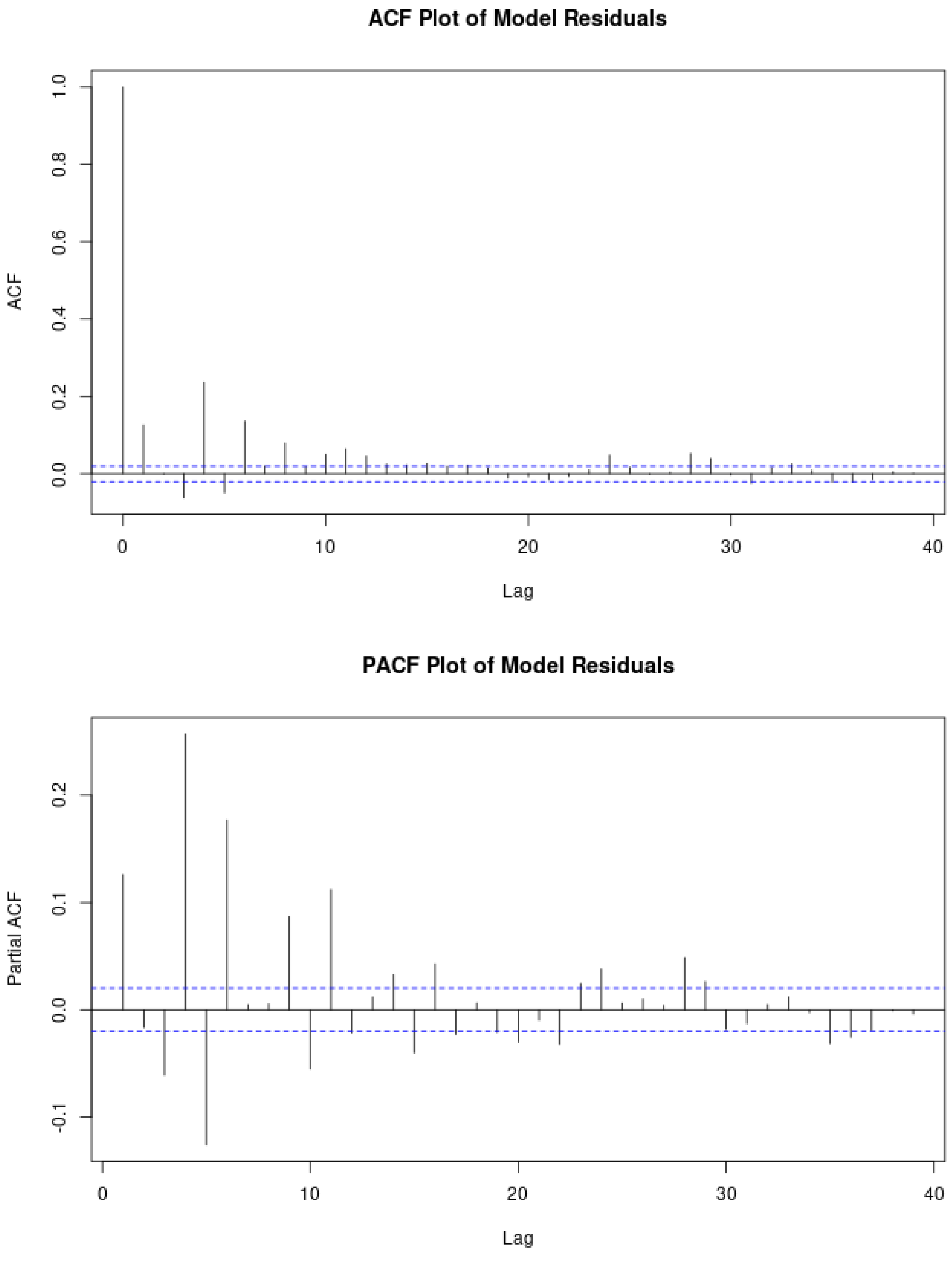

Supplementary File S2 Figure 8: Evaluation residuals of the linear ΔPbtO2 ~ ΔrSO2Lag1 models to ensure autocorrelative structure was accounted for. ACF (top) and PACF (bottom) plots of residuals of the model for Subject 8

Subject 9

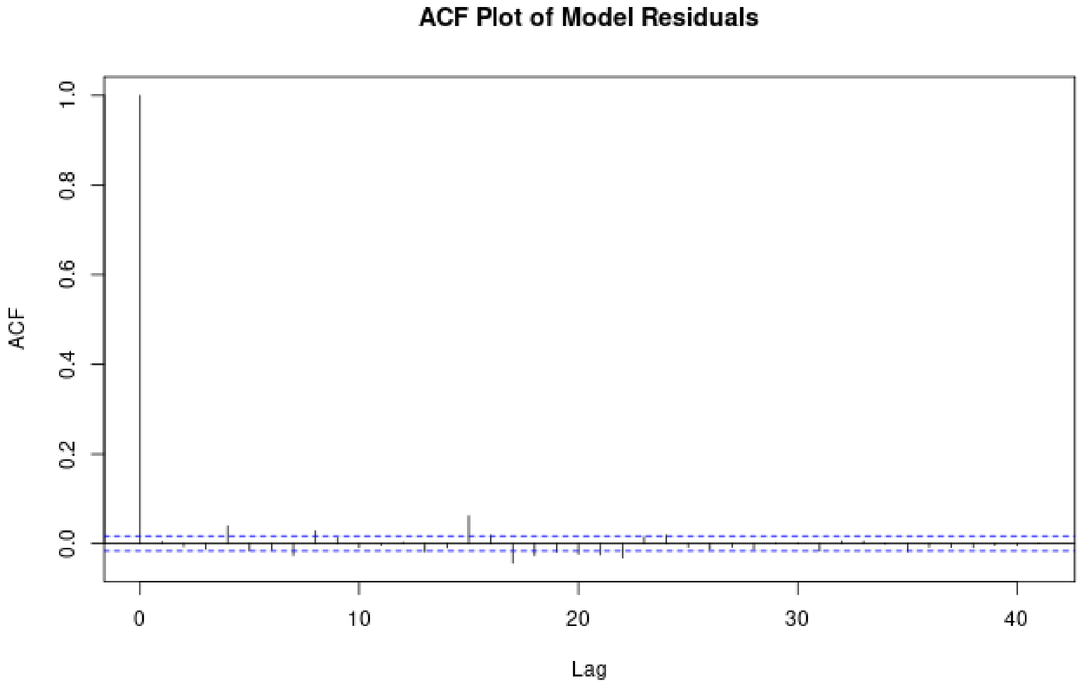

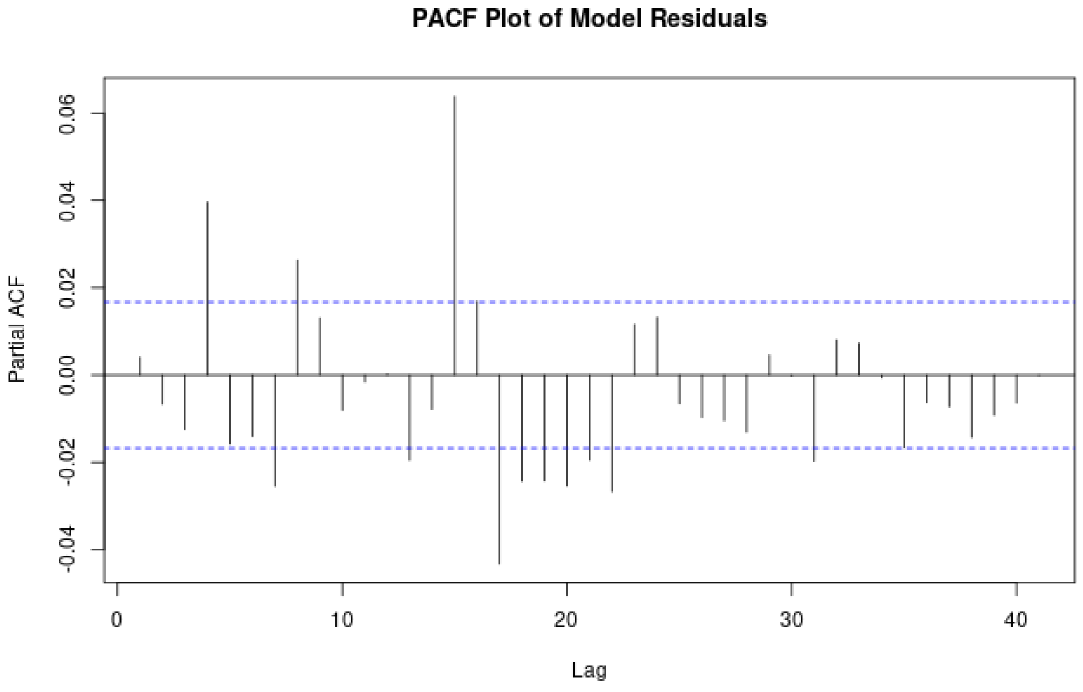

Supplementary File S2 Figure 9: Evaluation residuals of the linear ΔPbtO2 ~ ΔrSO2Lag1 models to ensure autocorrelative structure was accounted for. ACF (top) and PACF (bottom) plots of residuals of the model for Subject 9

Subject 10

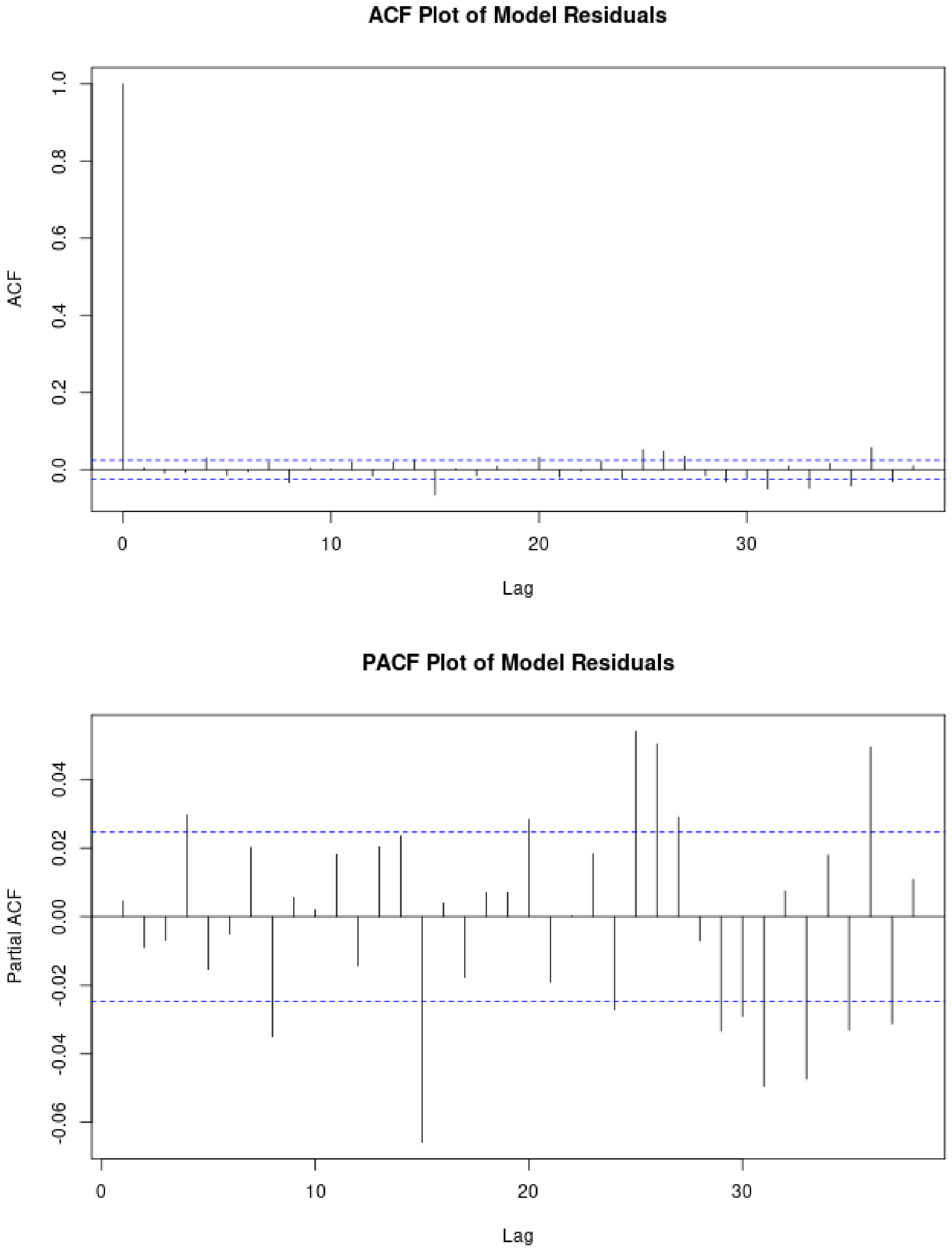

Supplementary File S2 Figure 10: Evaluation residuals of the linear ΔPbtO2 ~ ΔrSO2Lag1 models to ensure autocorrelative structure was accounted for. ACF (top) and PACF (bottom) plots of residuals of the model for Subject 10

Subject 11

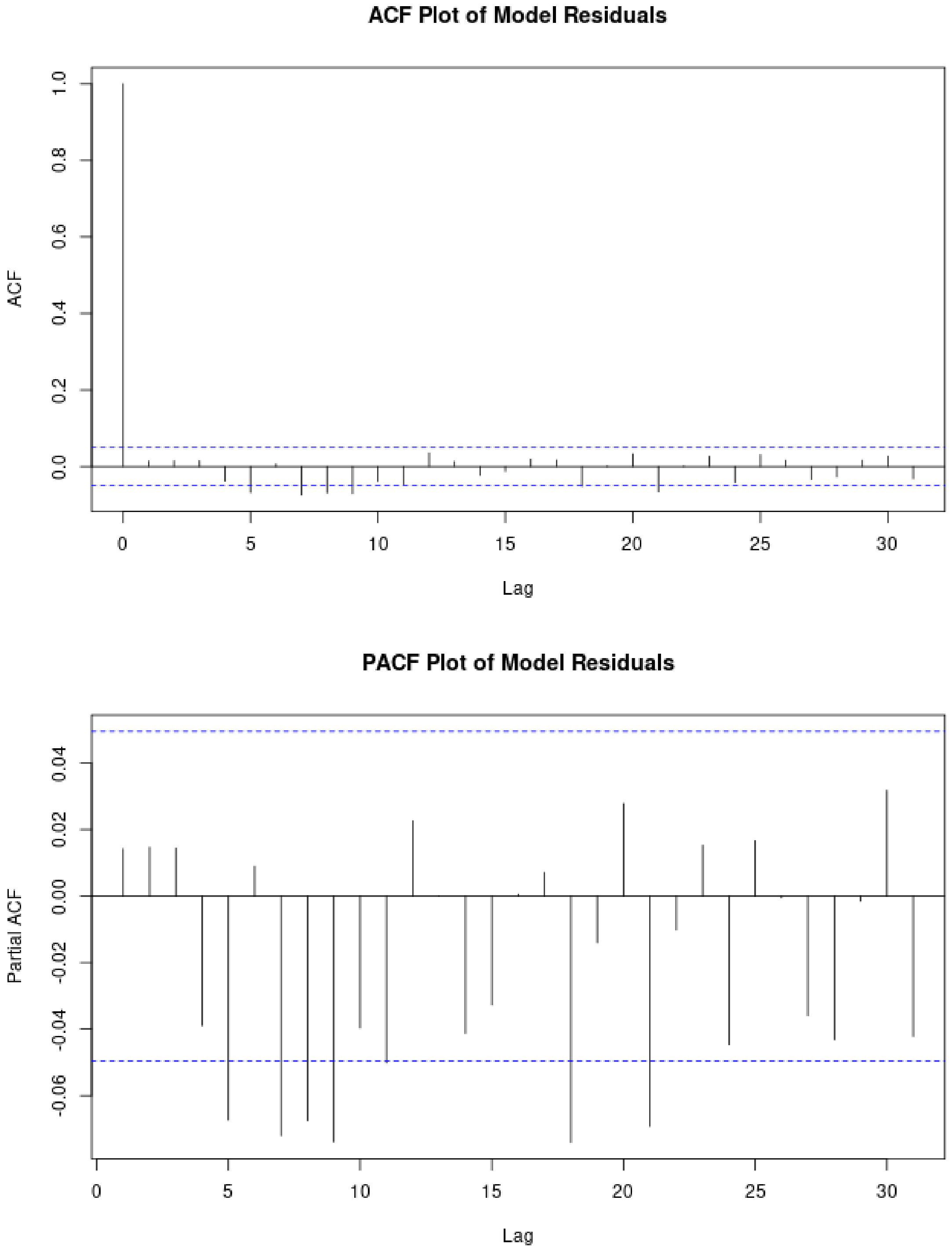

Supplementary File S2 Figure 11: Evaluation residuals of the linear ΔPbtO2 ~ ΔrSO2Lag1 models to ensure autocorrelative structure was accounted for. ACF (top) and PACF (bottom) plots of residuals of the model for Subject 11

Subject 12

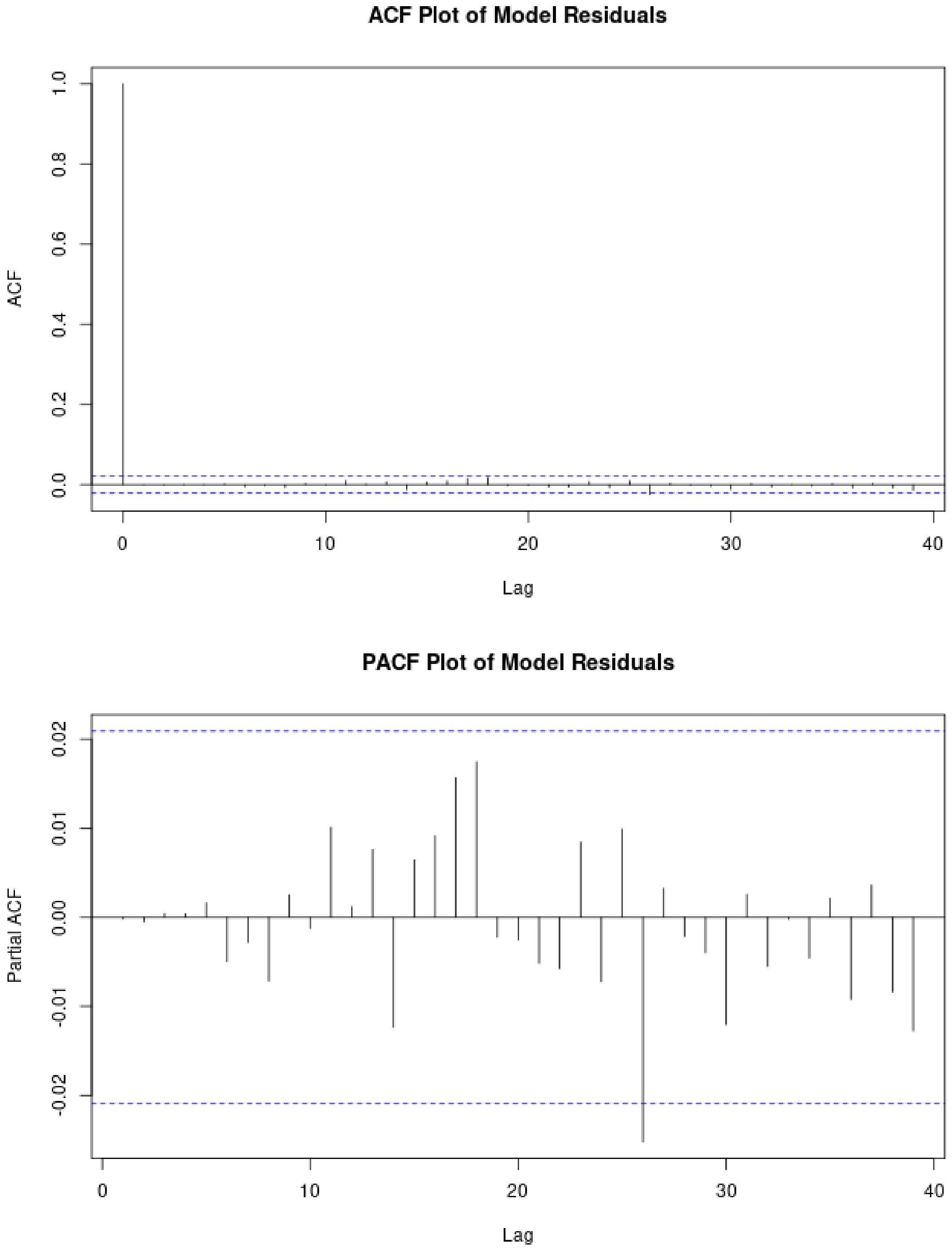

Supplementary File S2 Figure 12: Evaluation residuals of the linear ΔPbtO2 ~ ΔrSO2Lag1 models to ensure autocorrelative structure was accounted for. ACF (top) and PACF (bottom) plots of residuals of the model for Subject 12

Subject 13

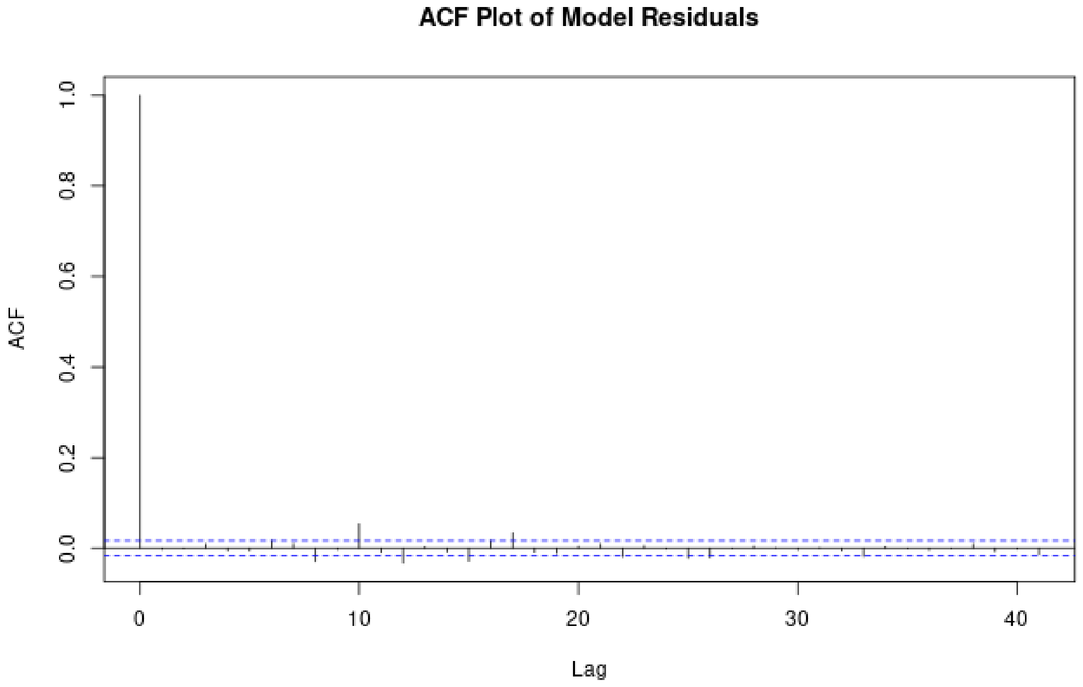

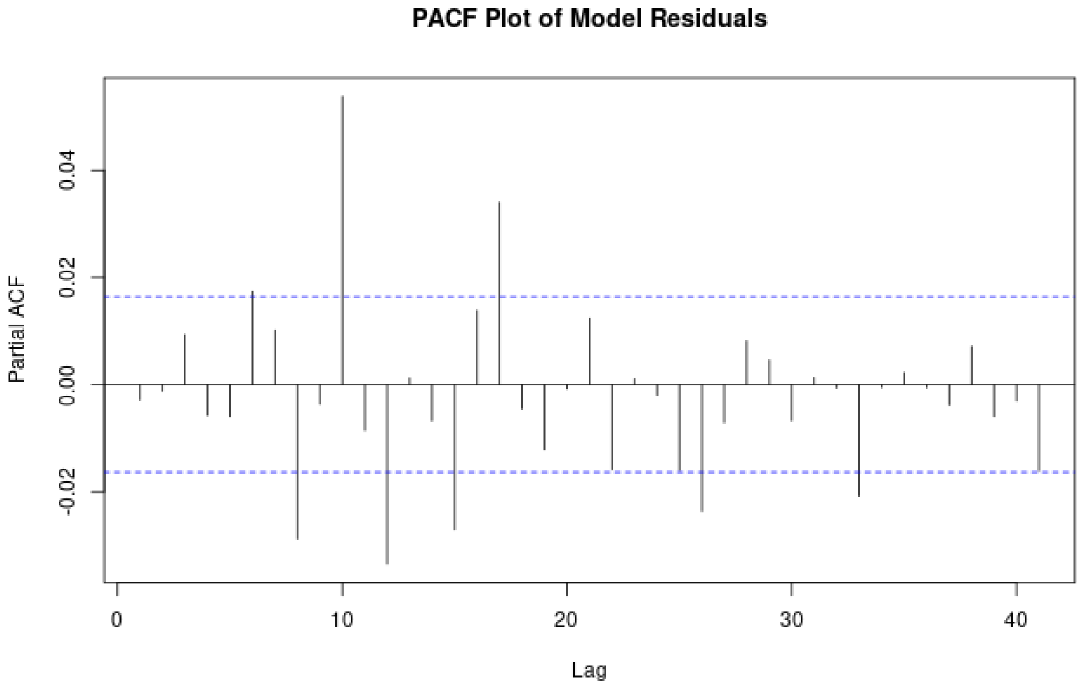

Supplementary File S2 Figure 13: Evaluation residuals of the linear ΔPbtO2 ~ ΔrSO2Lag1 models to ensure autocorrelative structure was accounted for. ACF (top) and PACF (bottom) plots of residuals of the model for Subject 13

Subject 14

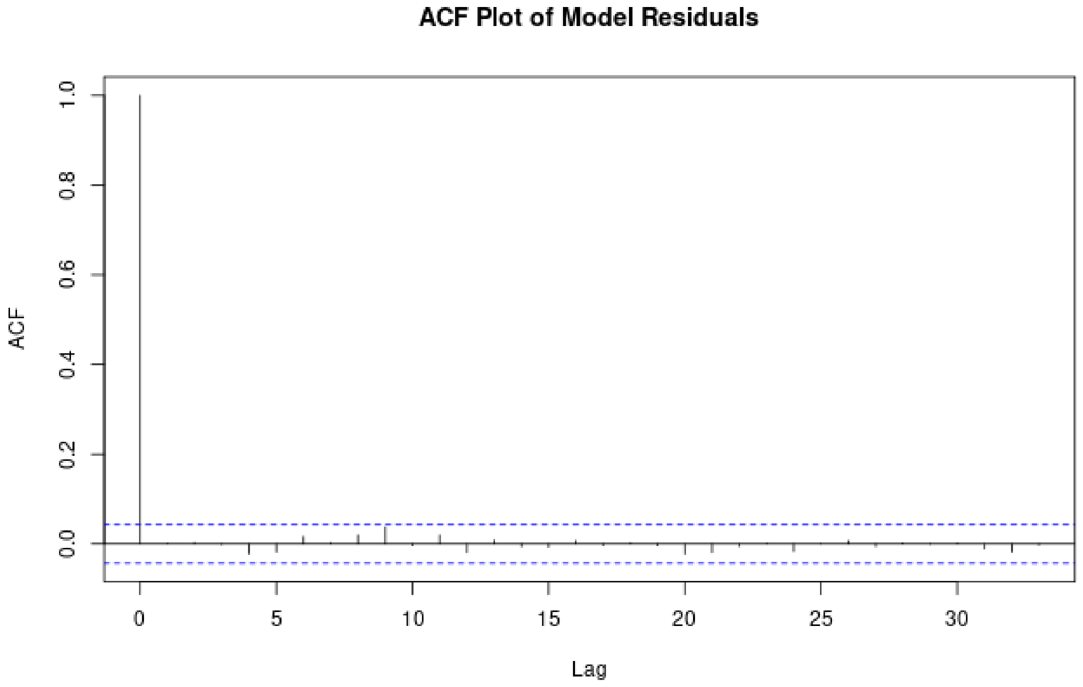

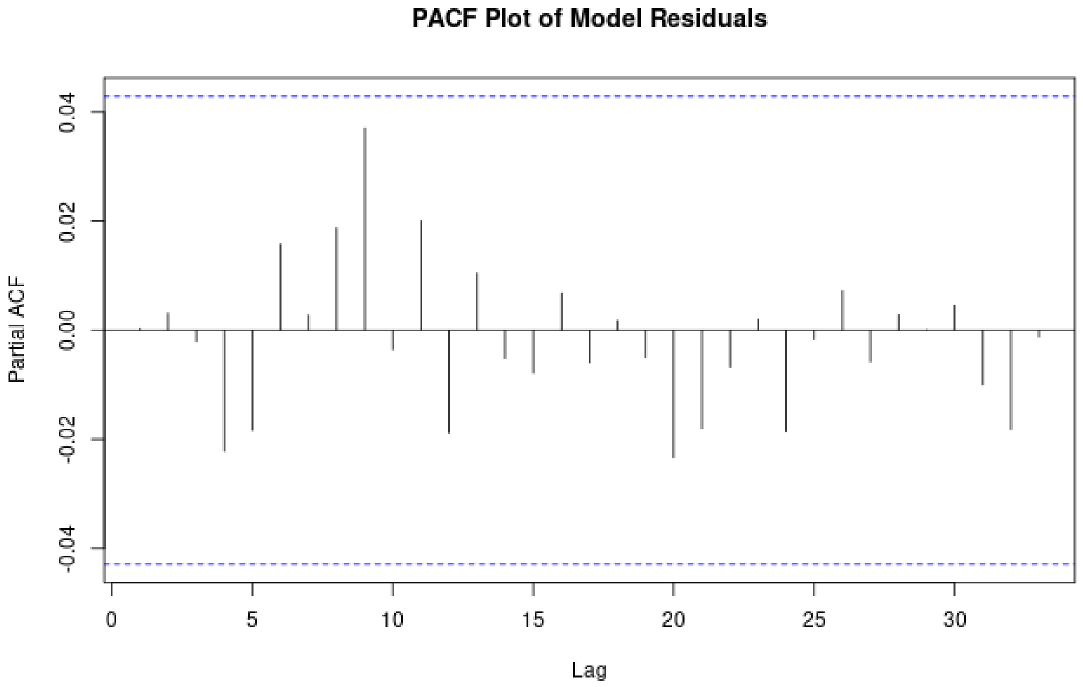

Supplementary File S2 Figure 14: Evaluation residuals of the linear ΔPbtO2 ~ ΔrSO2Lag1 models to ensure autocorrelative structure was accounted for. ACF (top) and PACF (bottom) plots of residuals of the model for Subject 14

Subject 15

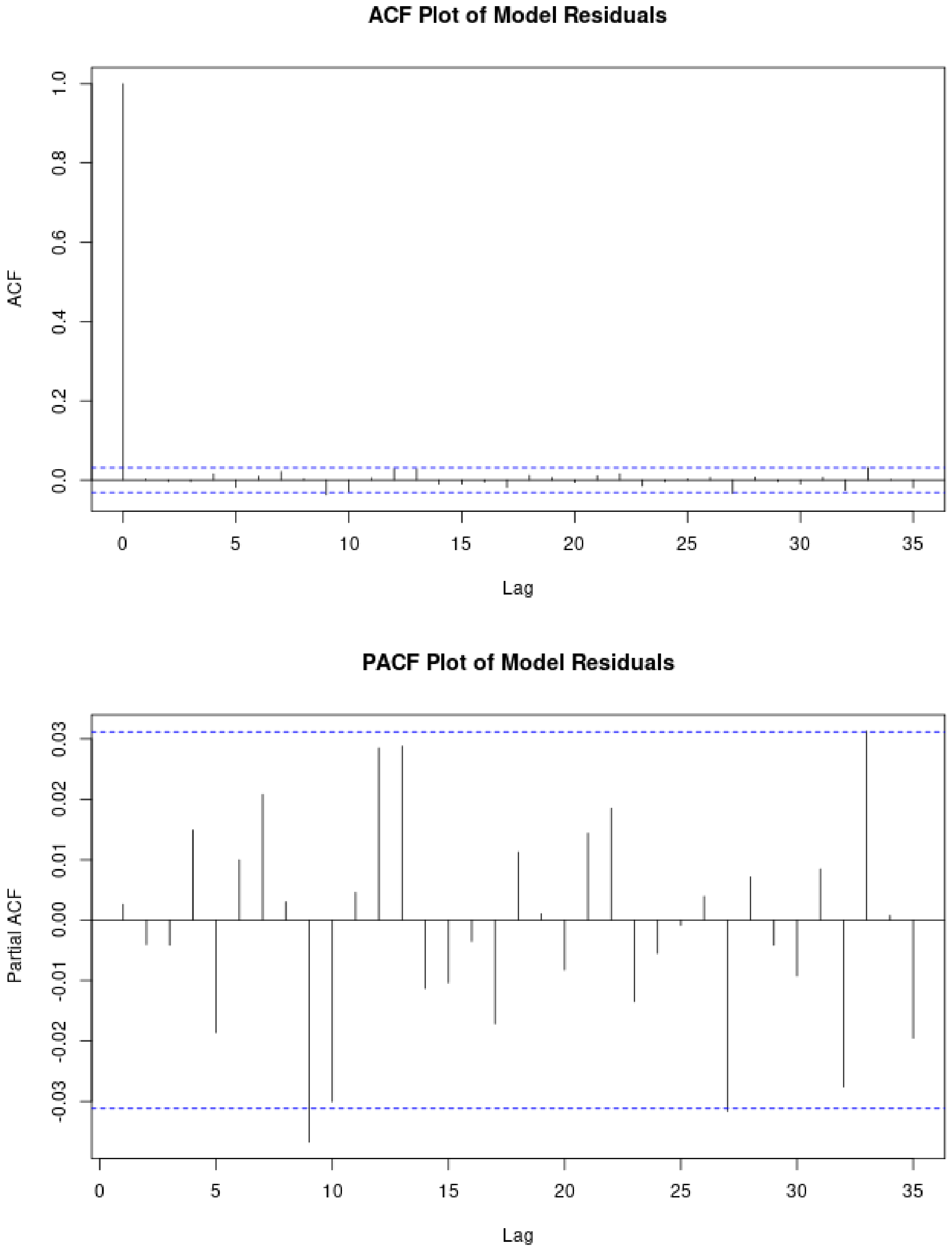

Supplementary File S2 Figure 15: Evaluation residuals of the linear ΔPbtO2 ~ ΔrSO2Lag1 models to ensure autocorrelative structure was accounted for. ACF (top) and PACF (bottom) plots of residuals of the model for Subject 15

Subject 16

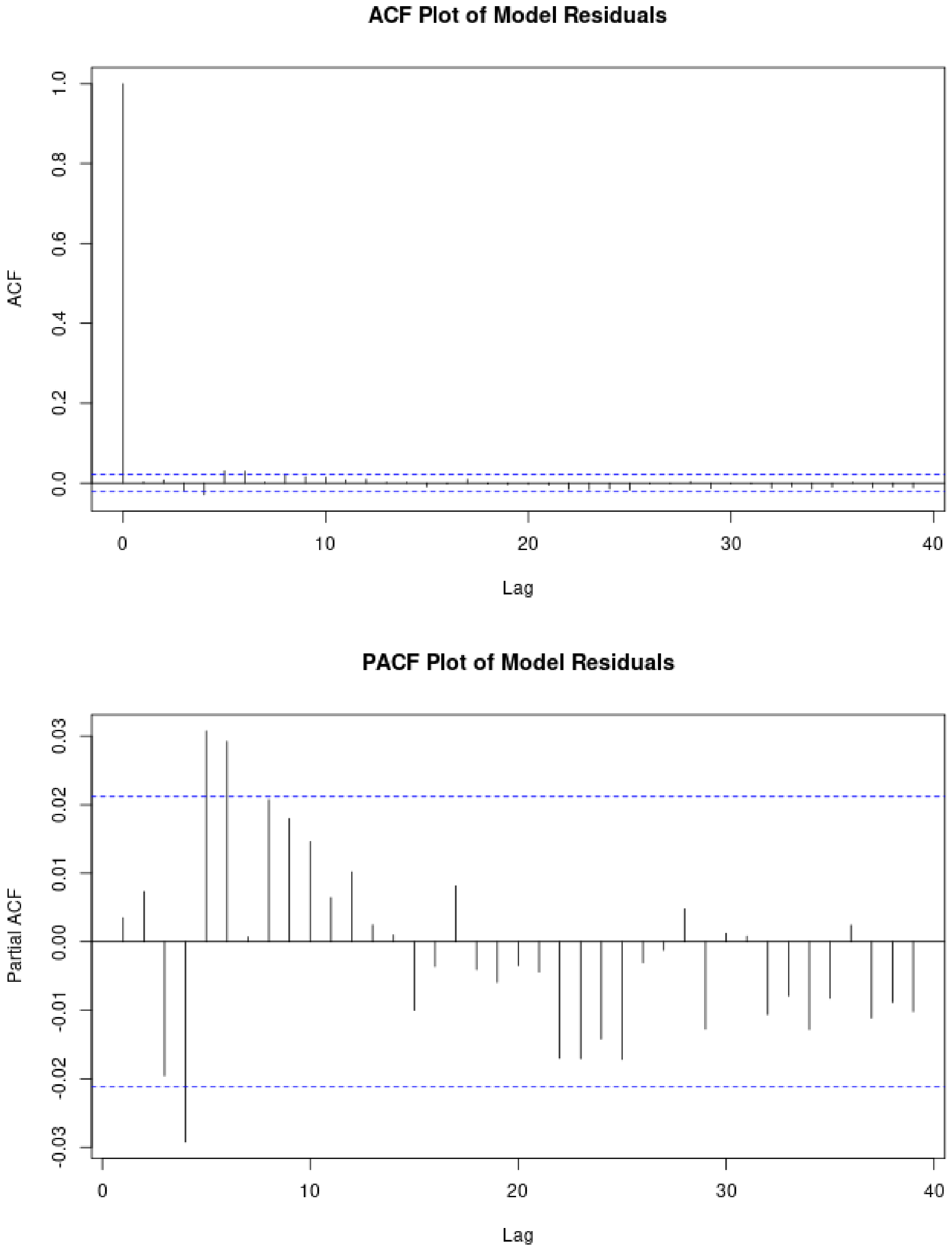

Supplementary File S2 Figure 16: Evaluation residuals of the linear ΔPbtO2 ~ ΔrSO2Lag1 models to ensure autocorrelative structure was accounted for. ACF (top) and PACF (bottom) plots of residuals of the model for Subject 16

Subject 17

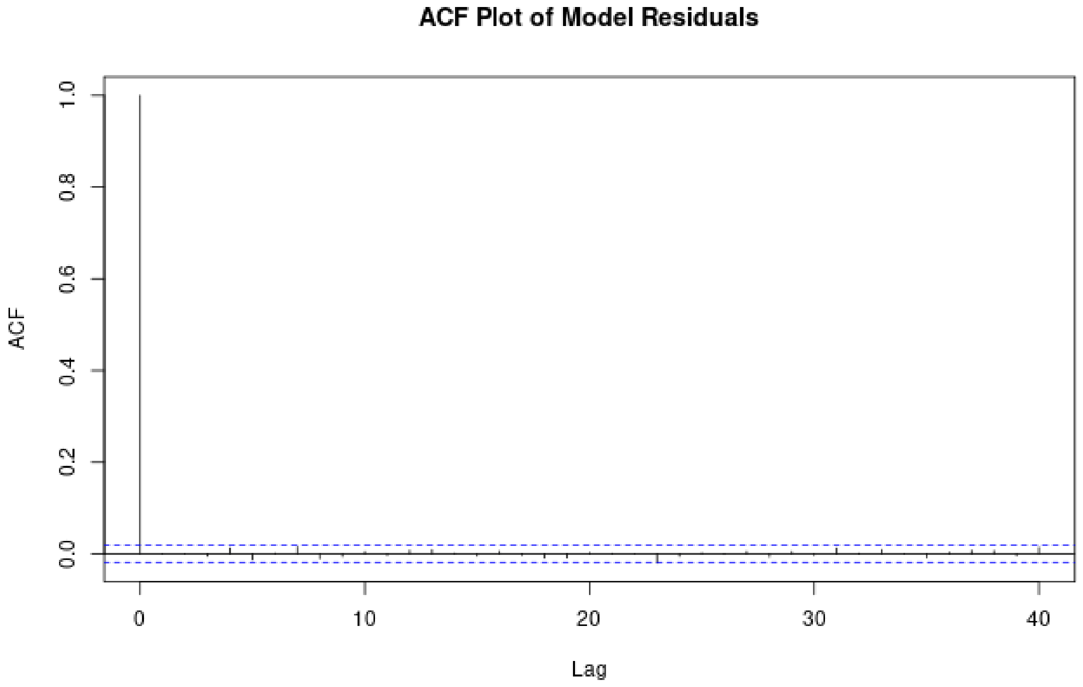

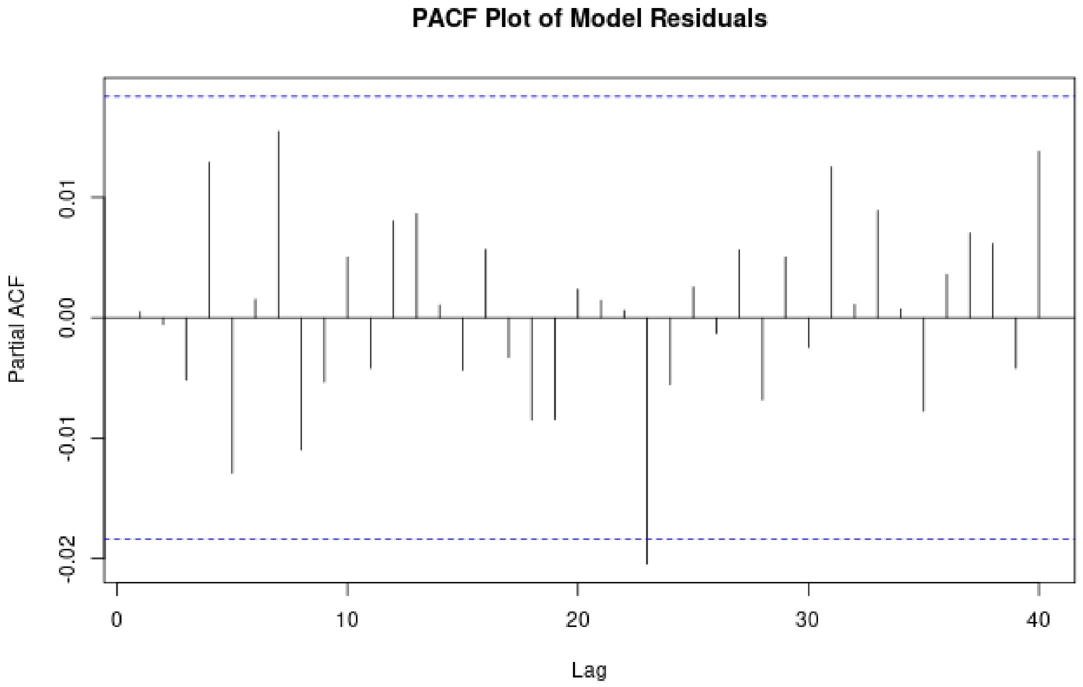

Supplementary File S2 Figure 17: Evaluation residuals of the linear ΔPbtO2 ~ ΔrSO2Lag1 models to ensure autocorrelative structure was accounted for. ACF (top) and PACF (bottom) plots of residuals of the model for Subject 17

Subject 18

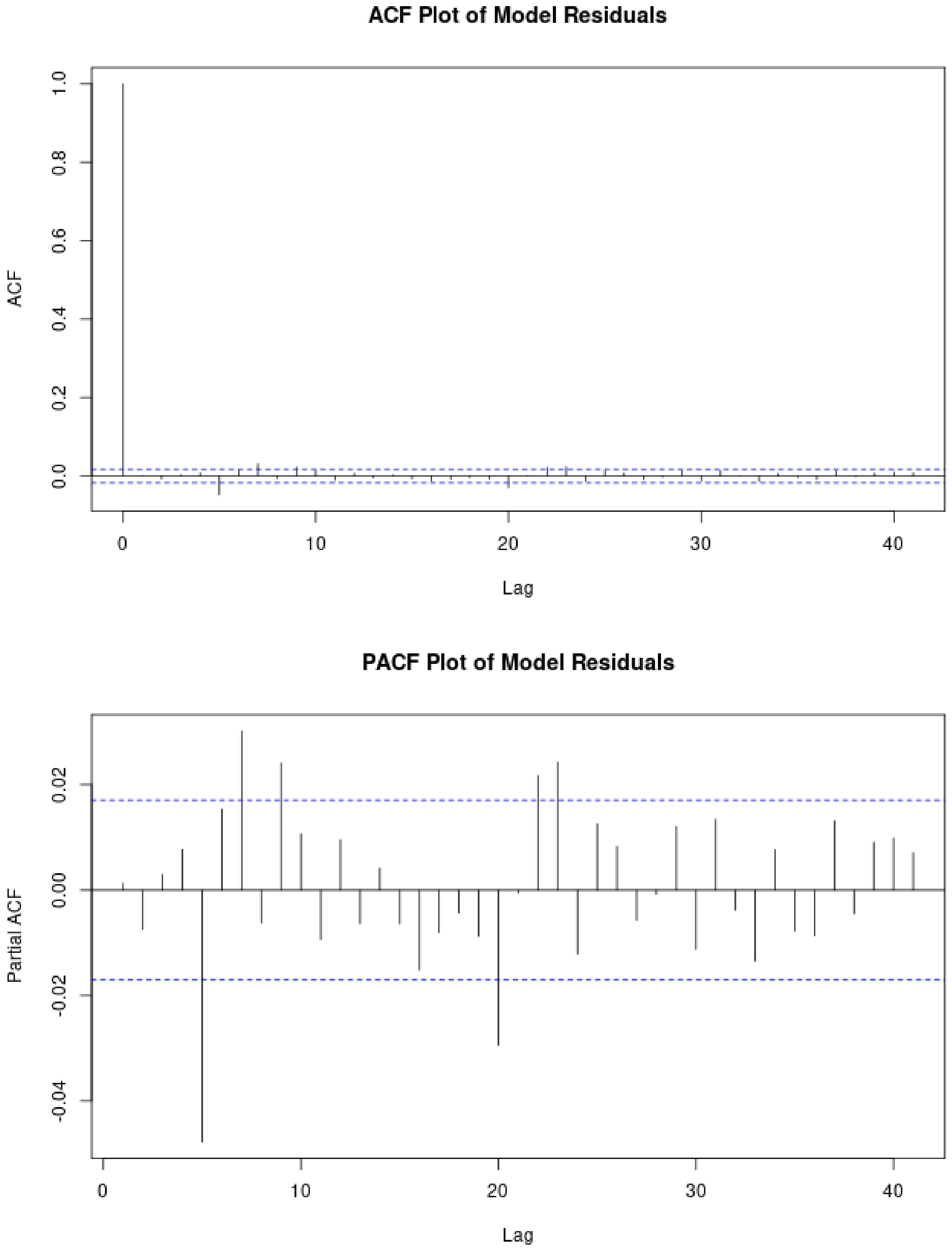

Supplementary File S2 Figure 18: Evaluation residuals of the linear ΔPbtO2 ~ ΔrSO2Lag1 models to ensure autocorrelative structure was accounted for. ACF (top) and PACF (bottom) plots of residuals of the model for Subject 18

Subject 19

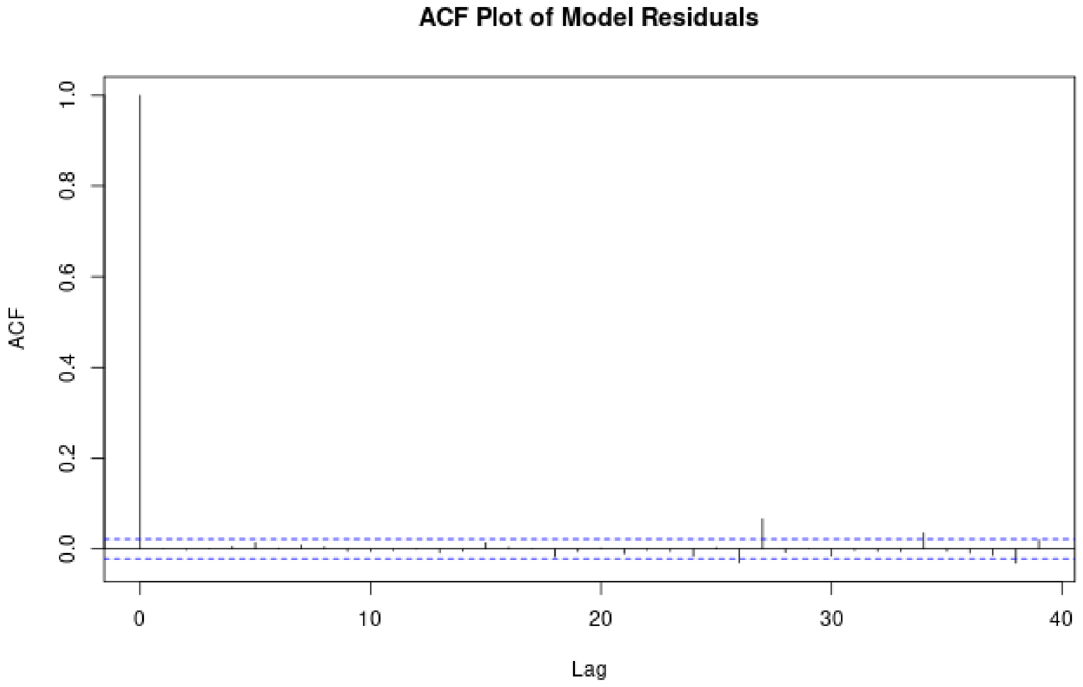

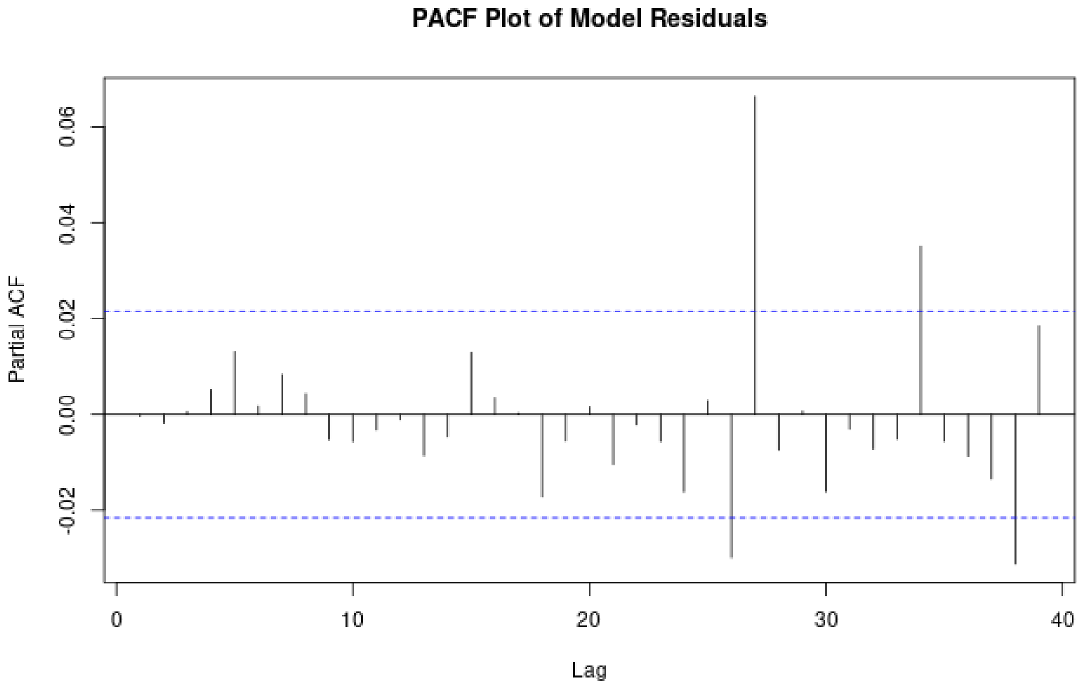

Supplementary File S2 Figure 19: Evaluation residuals of the linear ΔPbtO2 ~ ΔrSO2Lag1 models to ensure autocorrelative structure was accounted for. ACF (top) and PACF (bottom) plots of residuals of the model for Subject 19

Subject 20

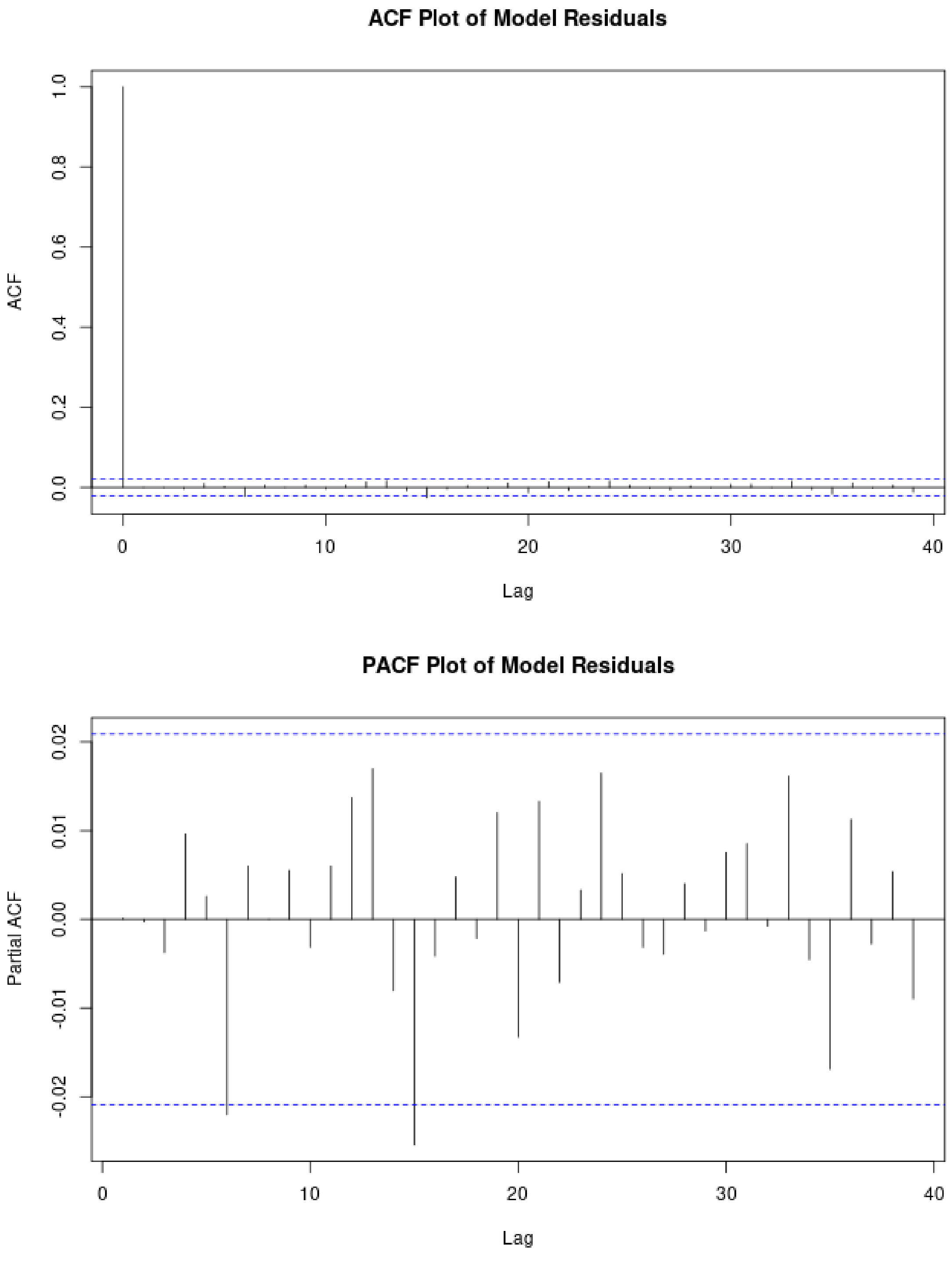

Supplementary File S2 Figure 20: Evaluation residuals of the linear ΔPbtO2 ~ ΔrSO2Lag1 models to ensure autocorrelative structure was accounted for. ACF (top) and PACF (bottom) plots of residuals of the model for Subject 20

3 Supplementary File S3: Scatter plots of actual vs predicted values of Δ$PbtO_2$ for each subject

Plot Legend: *ΔPbtO2 = change in brain tissue oxygen tension.*

Subject 1

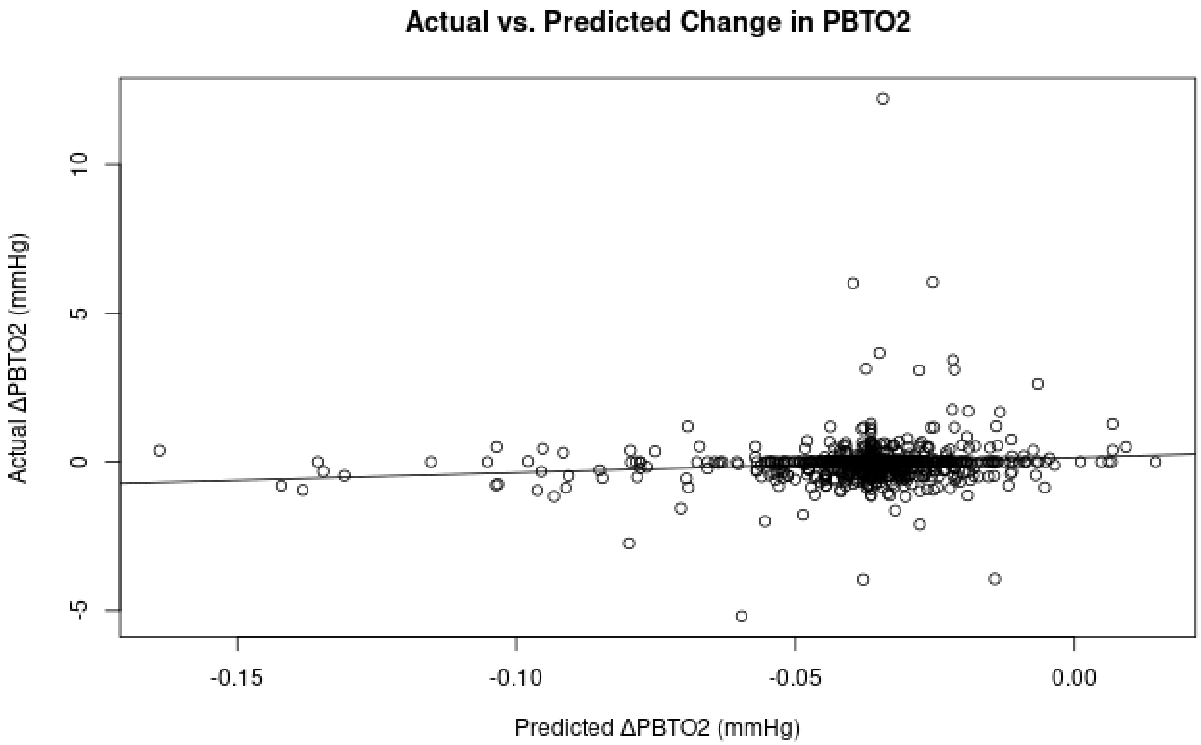

Supplementary File S3 Figure 1: Scatter plot of actual of actual vs predicted values of ΔPbtO2 for Subject 1.

Subject 2

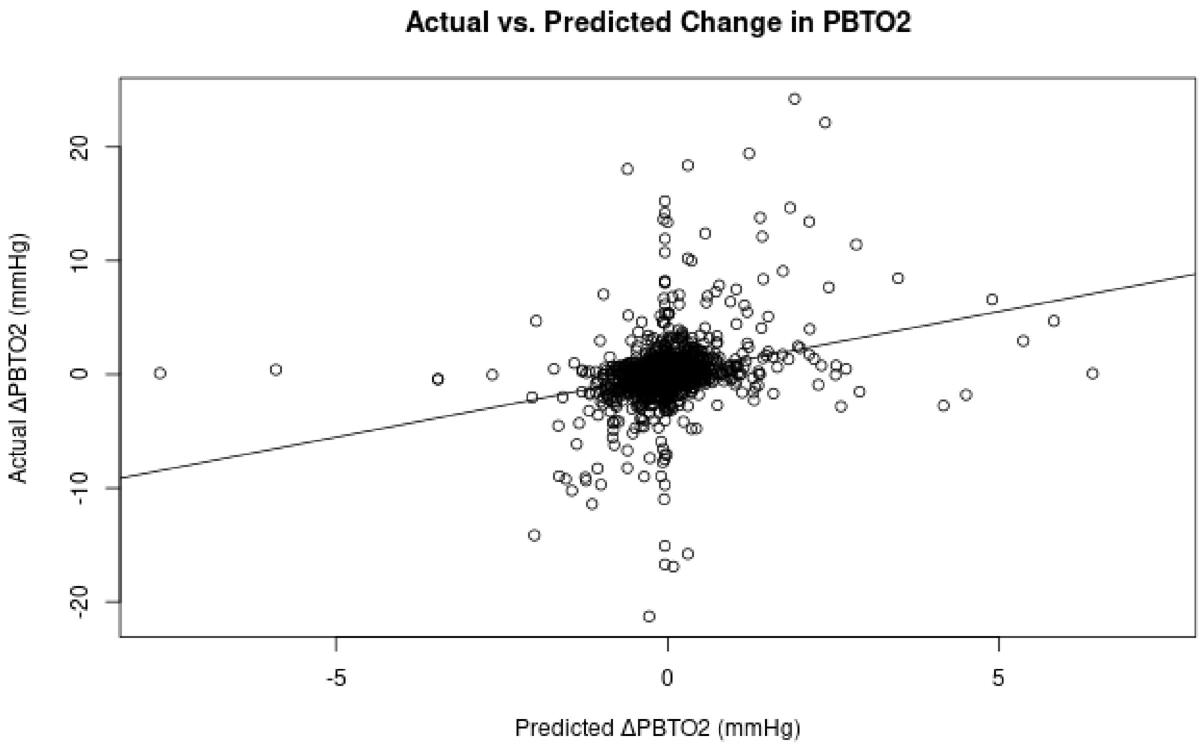

Supplementary File S3 Figure 2: Scatter plot of actual of actual vs predicted values of ΔPbtO2 for Subject 2.

Subject 3

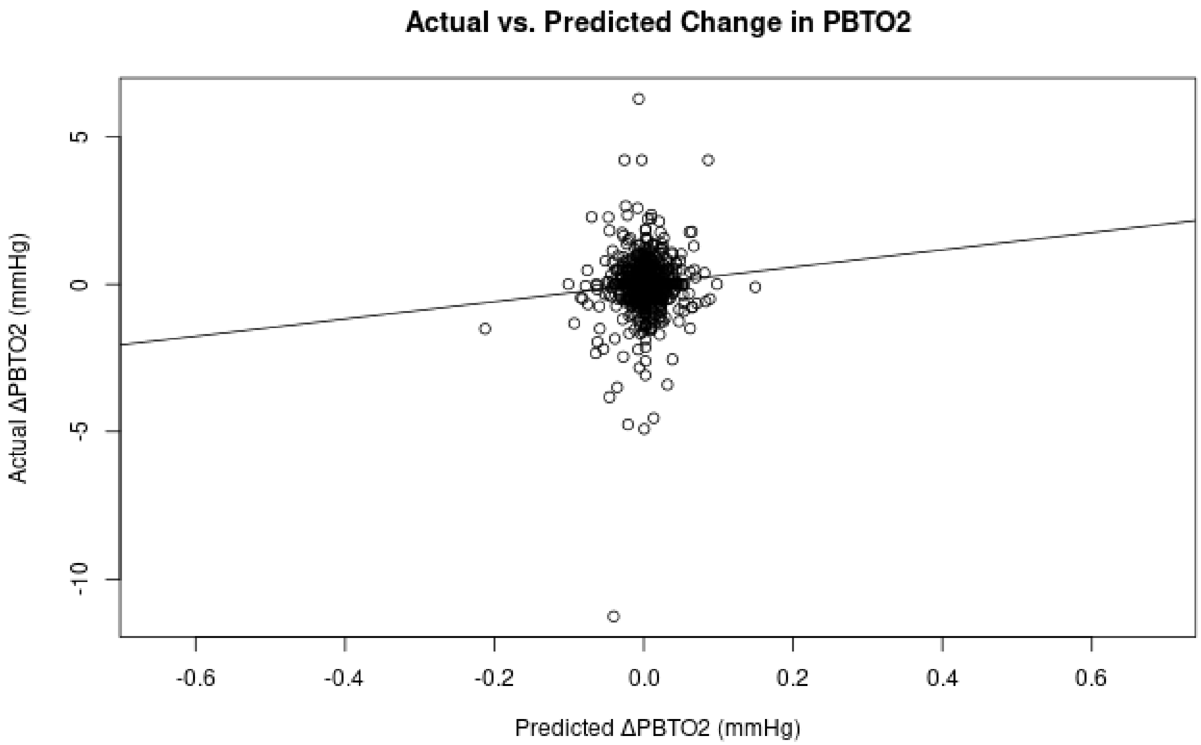

Supplementary File S3 Figure 3: Scatter plot of actual of actual vs predicted values of ΔPbtO2 for Subject 3.

Subject 4

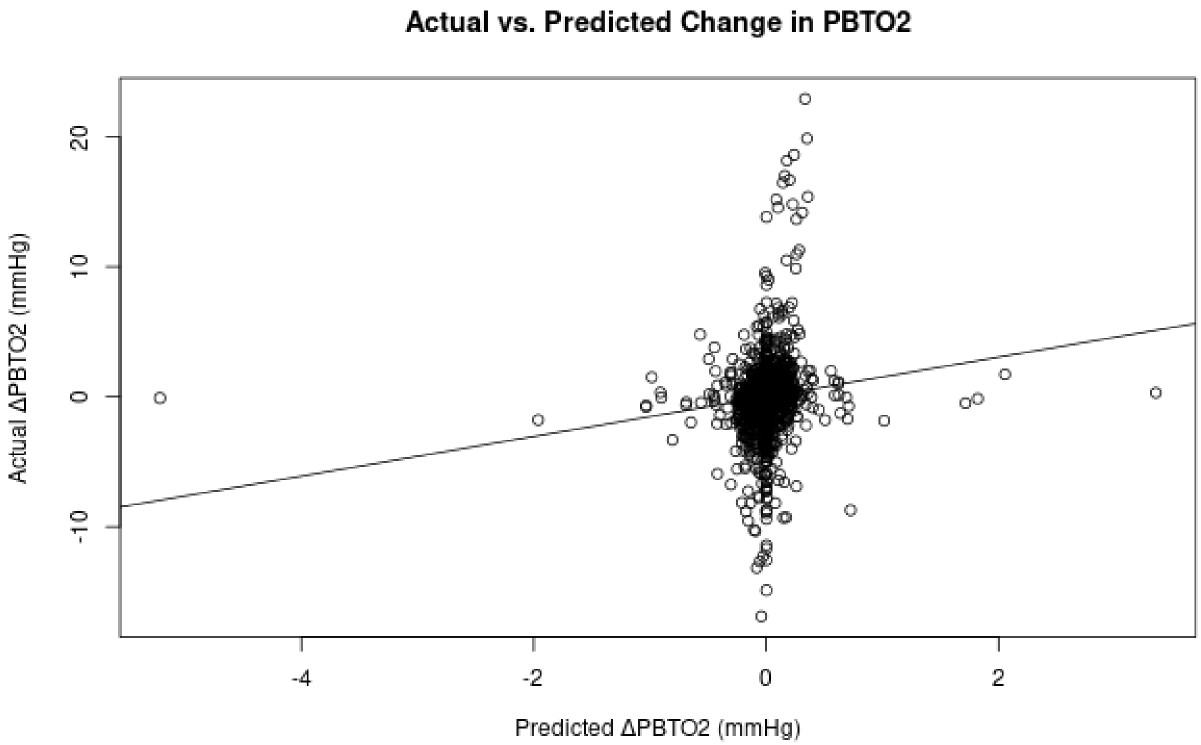

Supplementary File S3 Figure 4: Scatter plot of actual of actual vs predicted values of ΔPbtO2 for Subject 4.

Subject 5

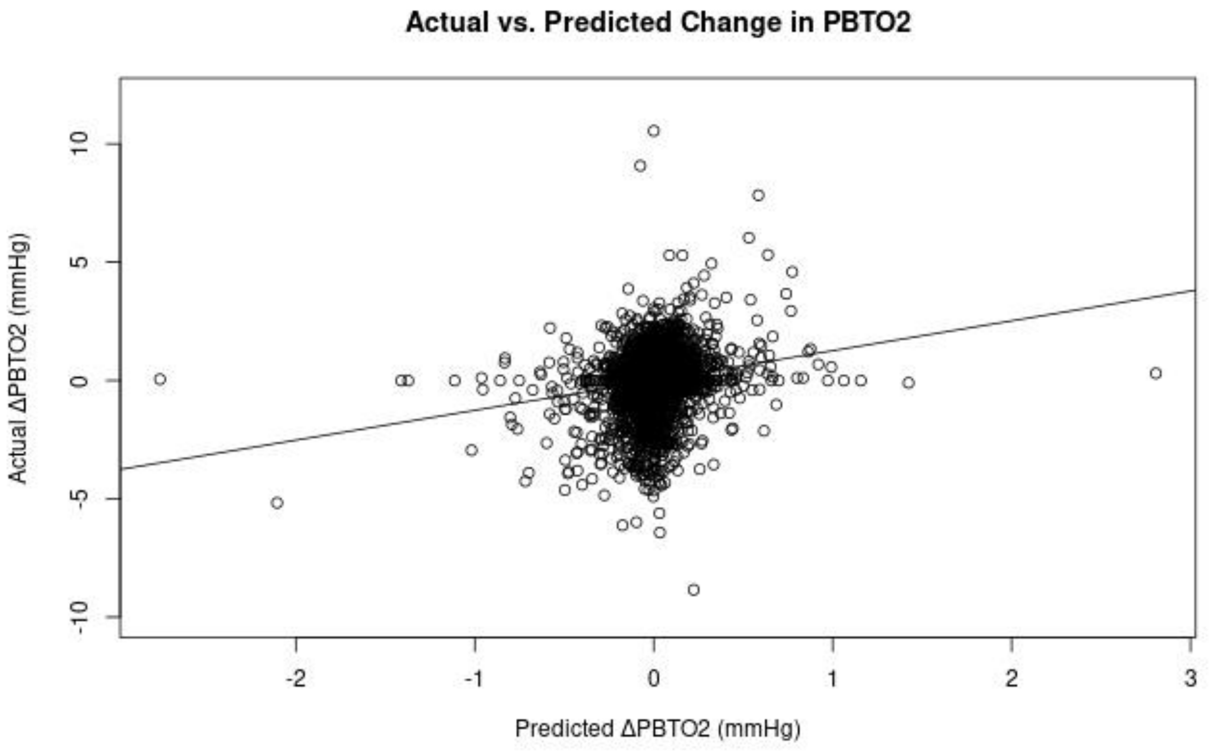

Supplementary File S3 Figure 5: Scatter plot of actual of actual vs predicted values of ΔPbtO2 for Subject 5.

Subject 6

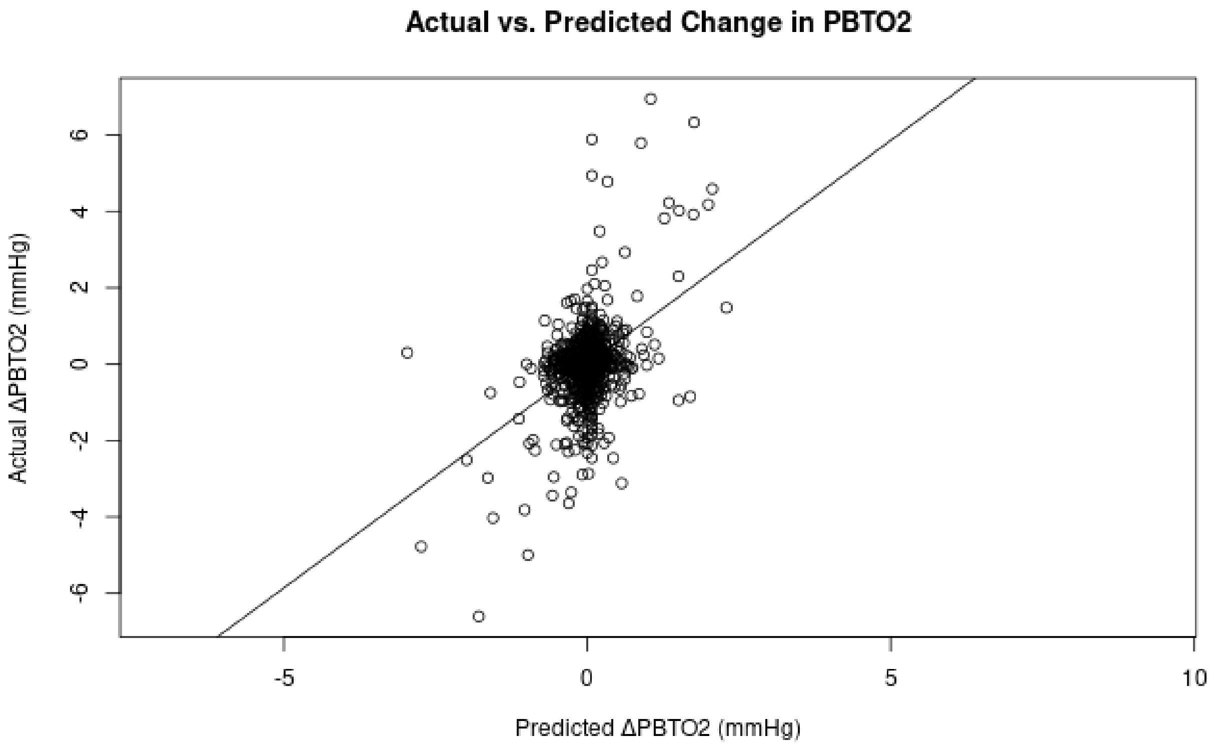

Supplementary File S3 Figure 6: Scatter plot of actual of actual vs predicted values of ΔPbtO2 for Subject 6.

Subject 7

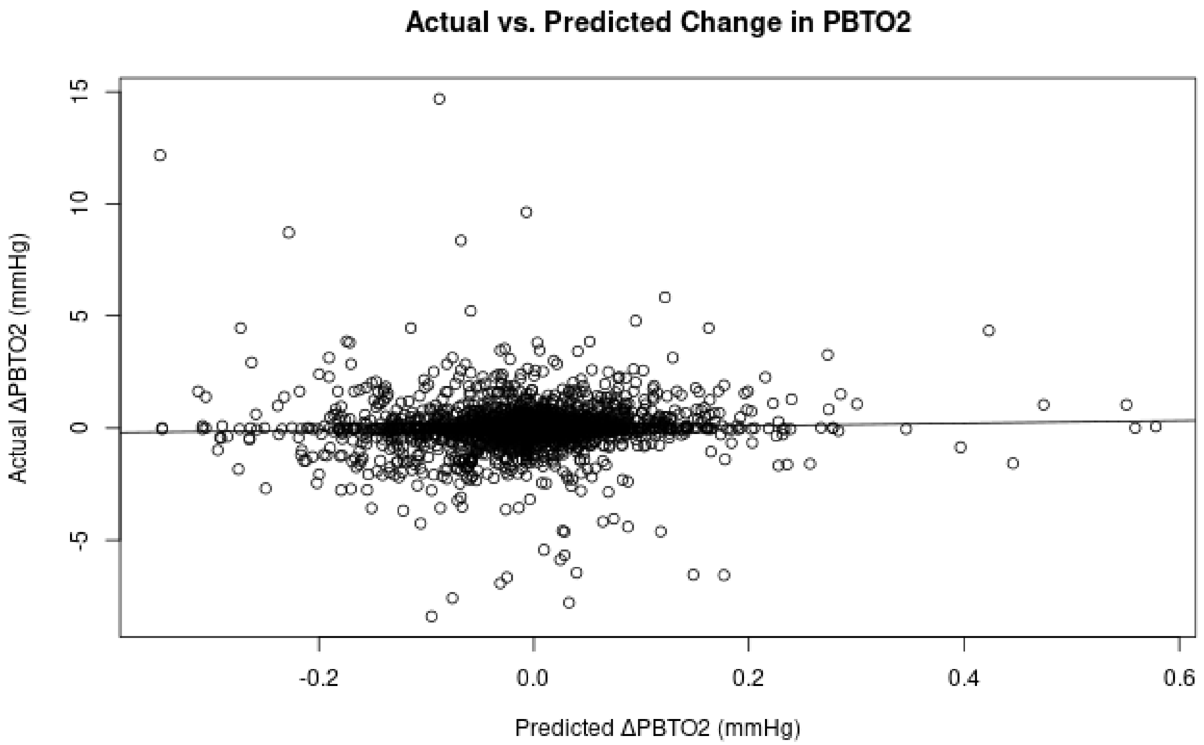

Supplementary File S3 Figure 7: Scatter plot of actual of actual vs predicted values of ΔPbtO2 for Subject 7.

Subject 8

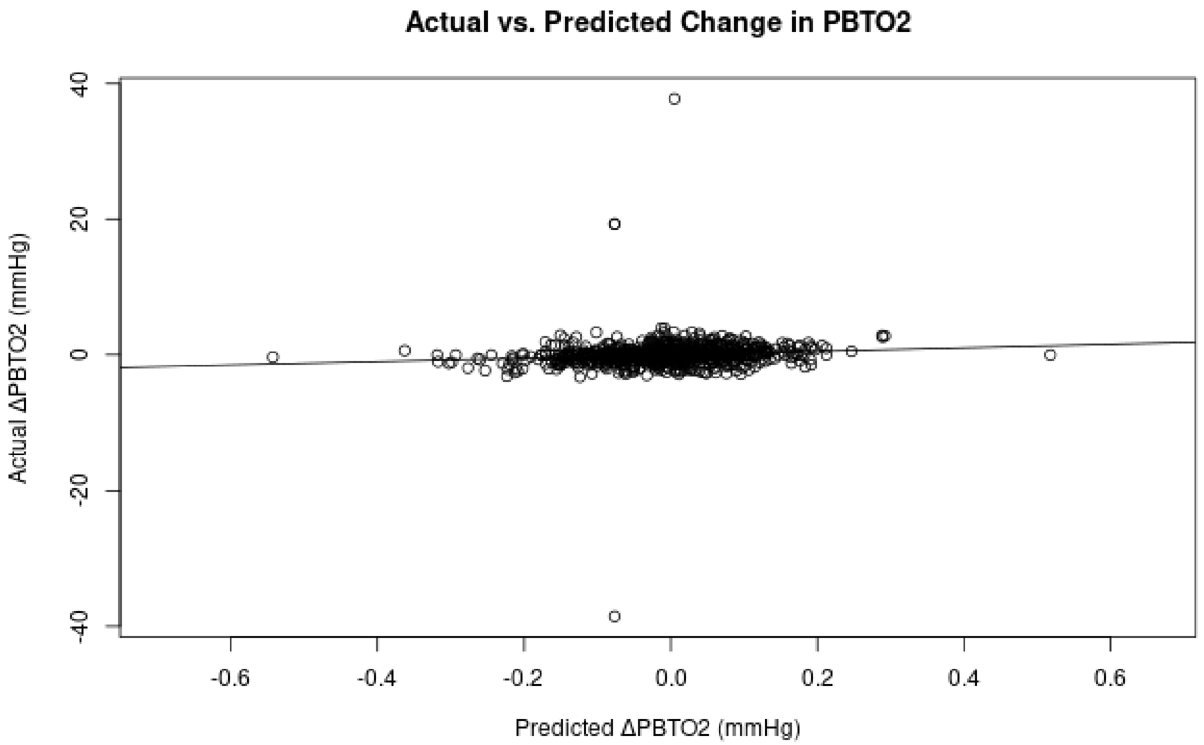

Supplementary File S3 Figure 8: Scatter plot of actual of actual vs predicted values of ΔPbtO2 for Subject 8.

Subject 9

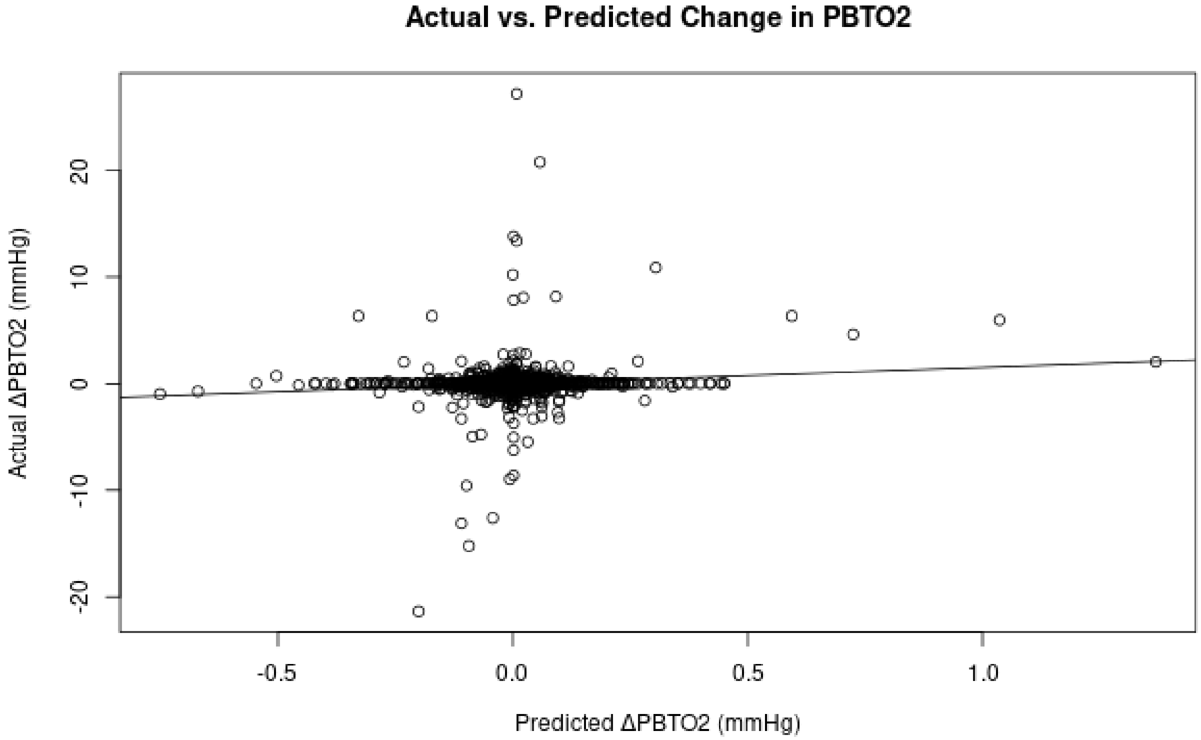

Supplementary File S3 Figure 9: Scatter plot of actual of actual vs predicted values of ΔPbtO2 for Subject 9.

Subject 10

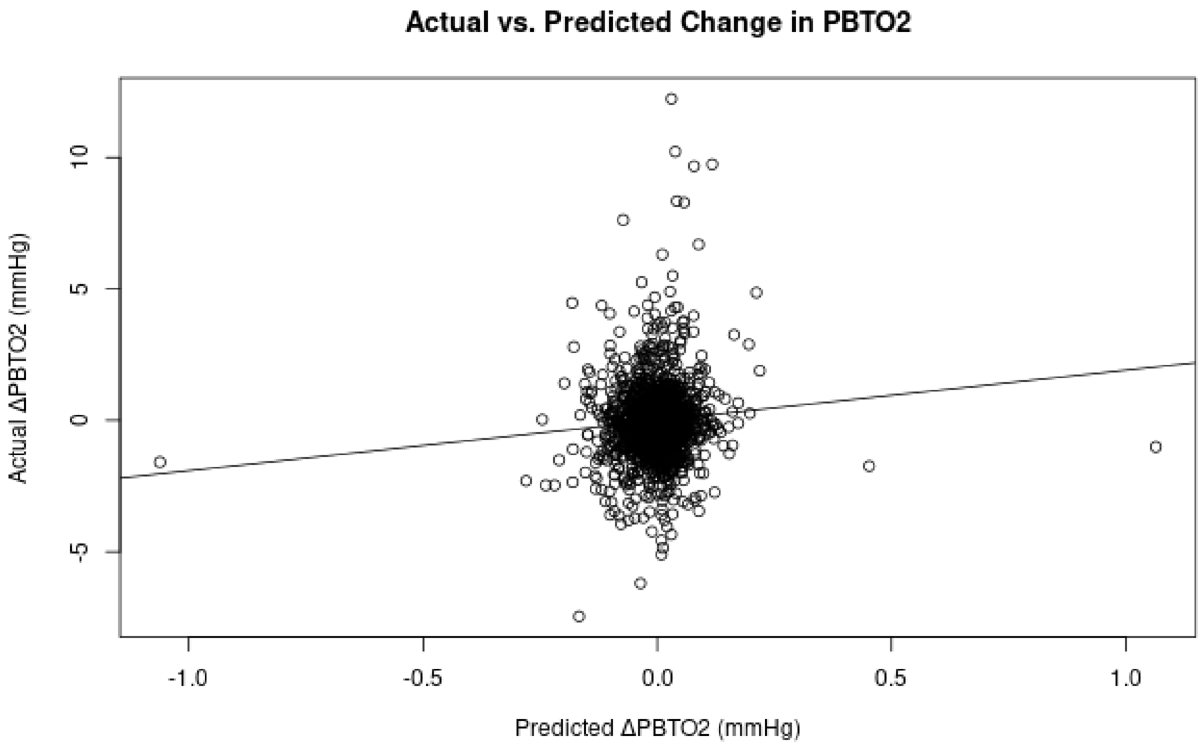

Supplementary File S3 Figure 10: Scatter plot of actual of actual vs predicted values of ΔPbtO2 for Subject 10.

Subject 11

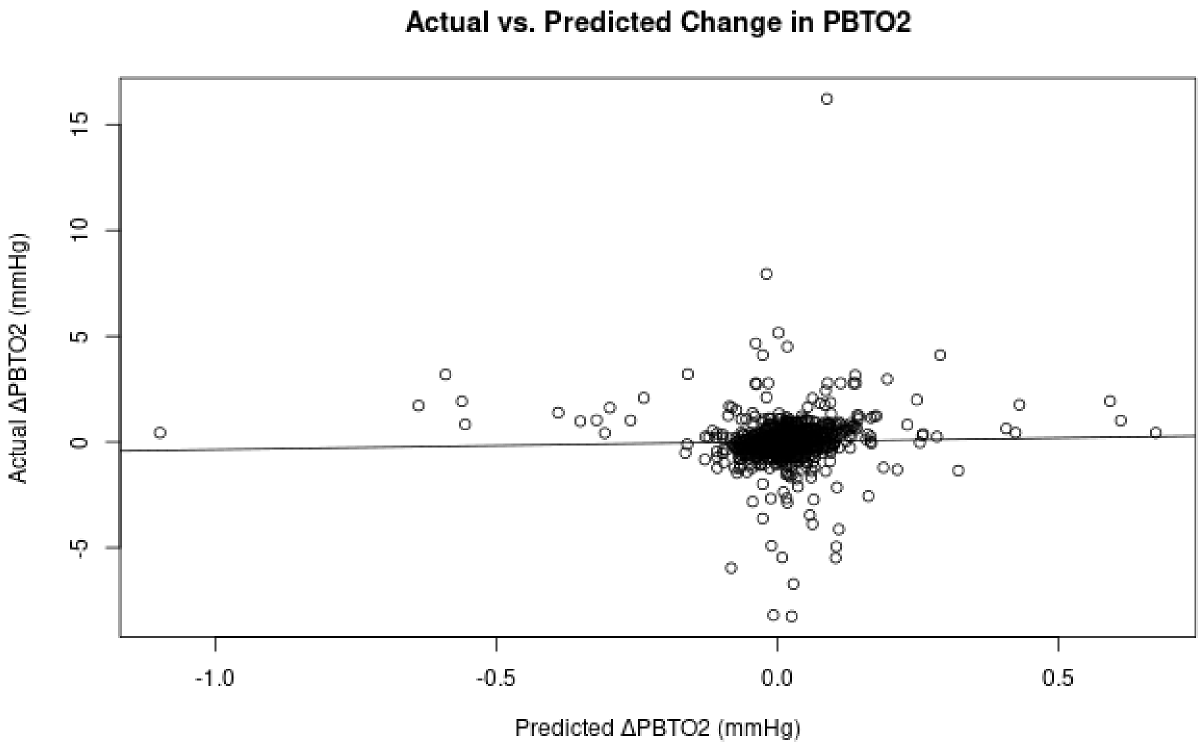

Supplementary File S3 Figure 11: Scatter plot of actual of actual vs predicted values of ΔPbtO2 for Subject 11.

Subject 12

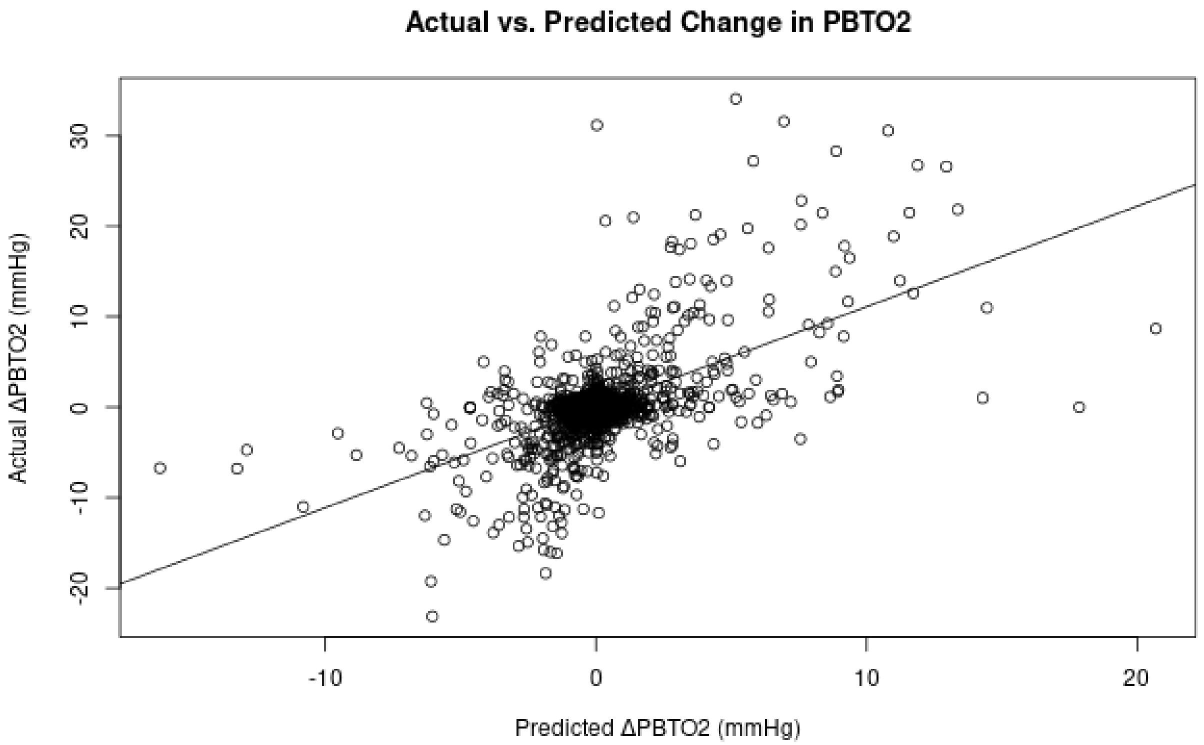

Supplementary File S3 Figure 12: Scatter plot of actual of actual vs predicted values of ΔPbtO2 for Subject 12.

Subject 13

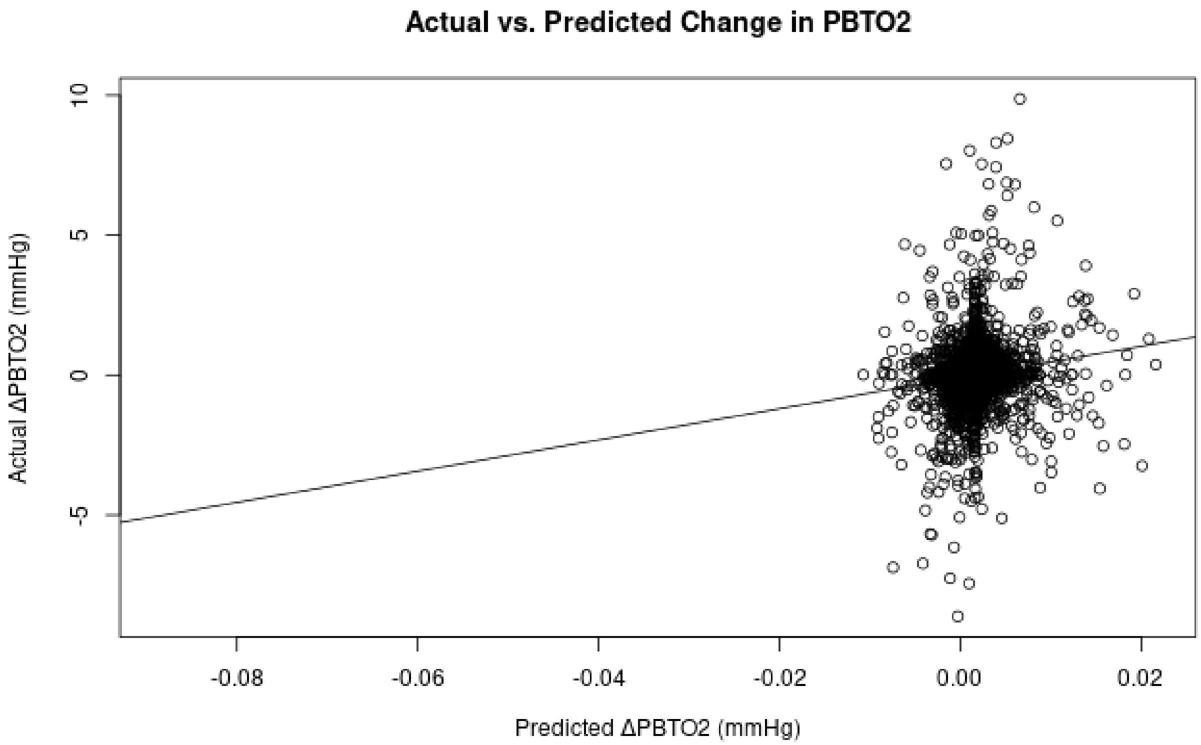

Supplementary File S3 Figure 13: Scatter plot of actual of actual vs predicted values of ΔPbtO2 for Subject 13.

Subject 14

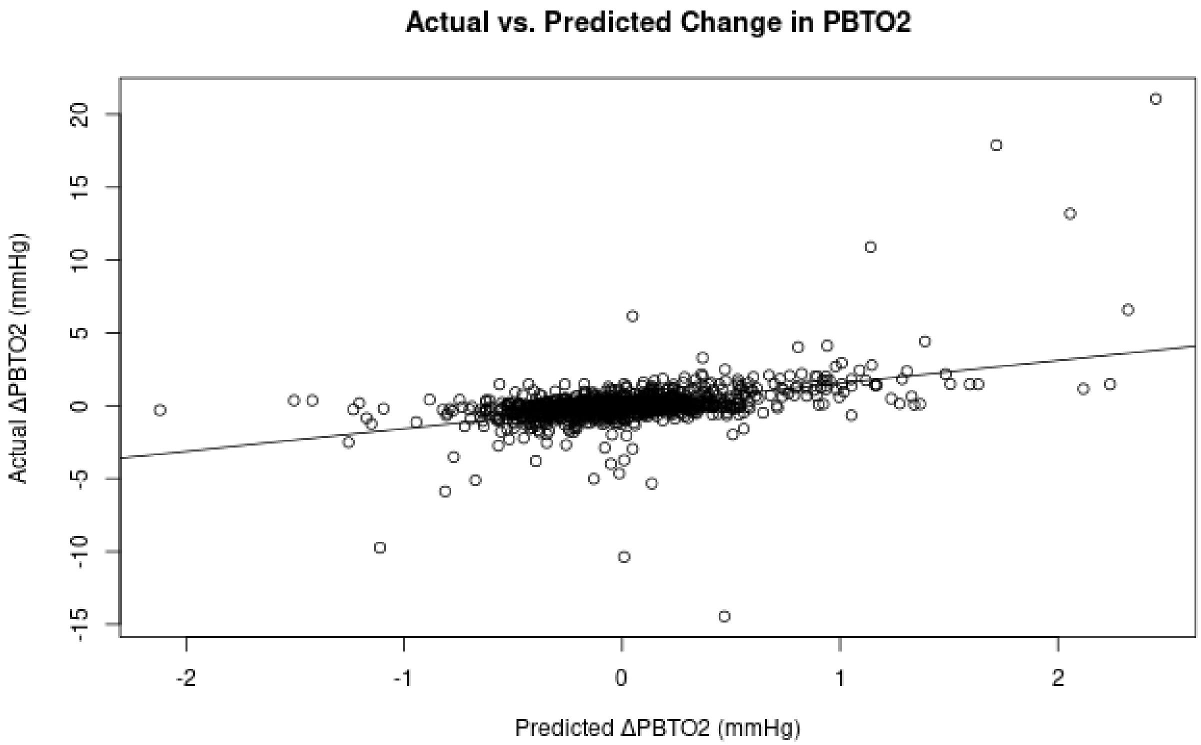

Supplementary File S3 Figure 14: Scatter plot of actual of actual vs predicted values of ΔPbtO2 for Subject 14.

Subject 15

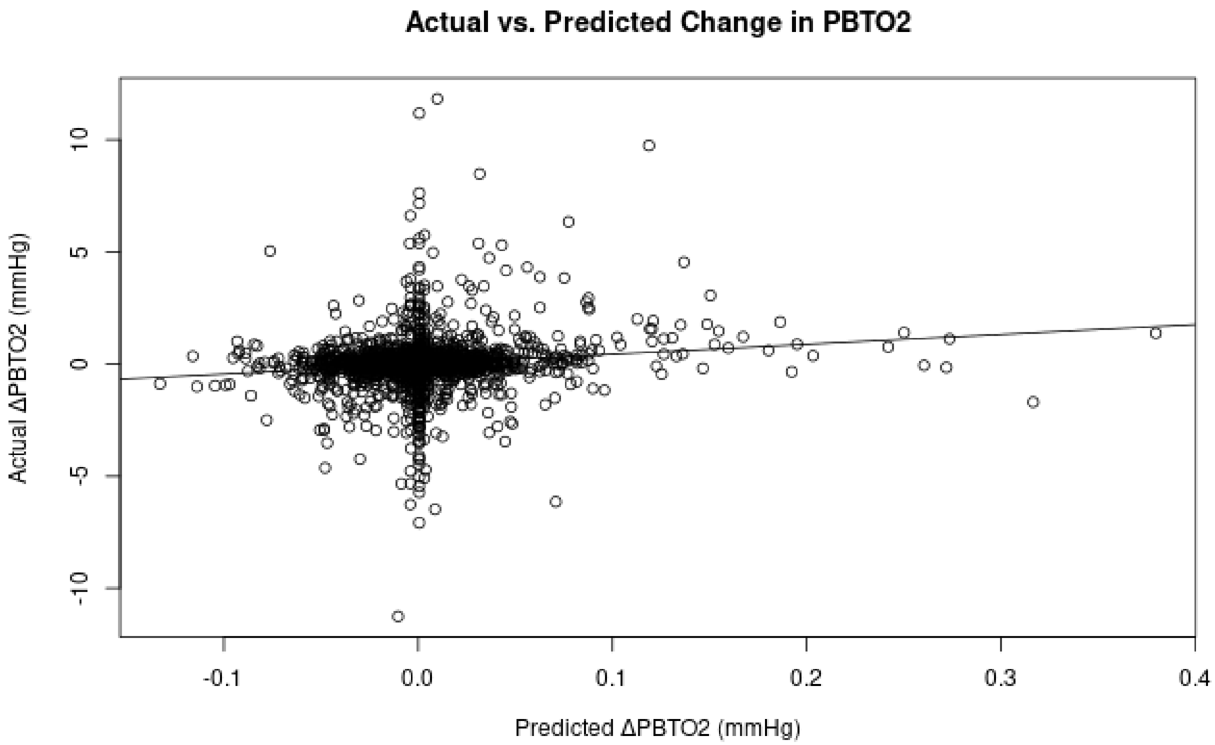

Supplementary File S3 Figure 15: Scatter plot of actual of actual vs predicted values of ΔPbtO2 for Subject 15.

Subject 16

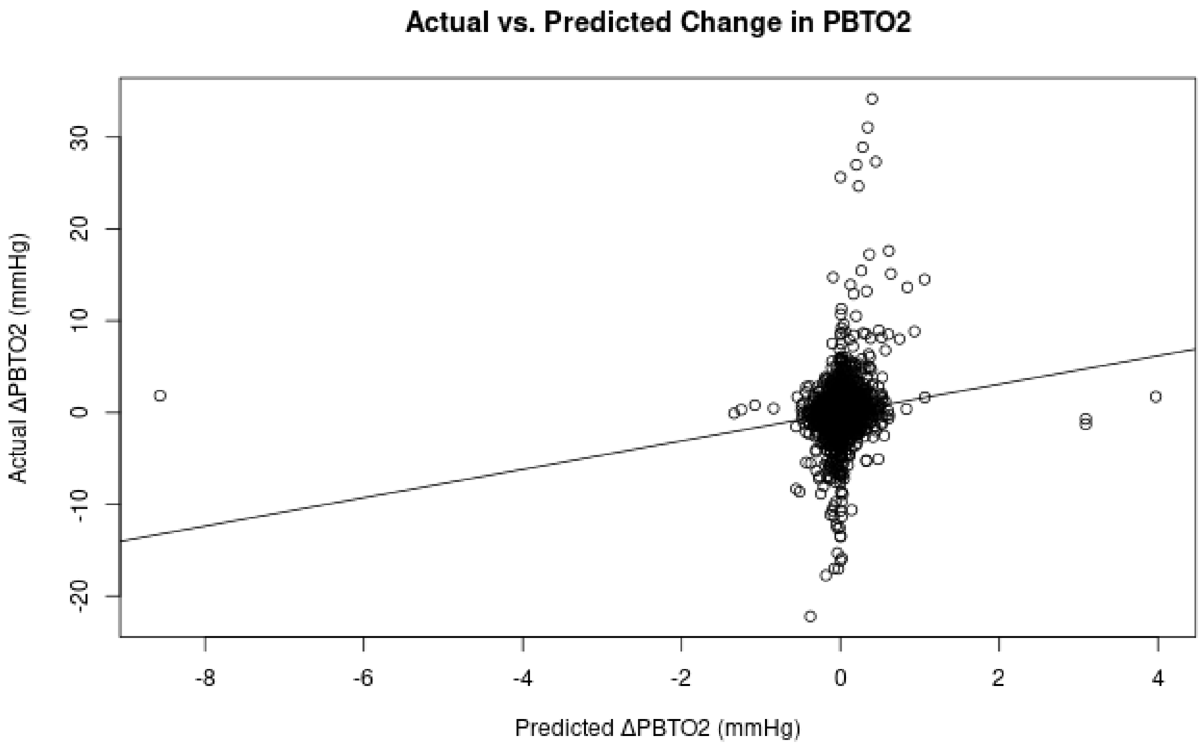

Supplementary File S3 Figure 16: Scatter plot of actual of actual vs predicted values of ΔPbtO2 for Subject 16.

Subject 17

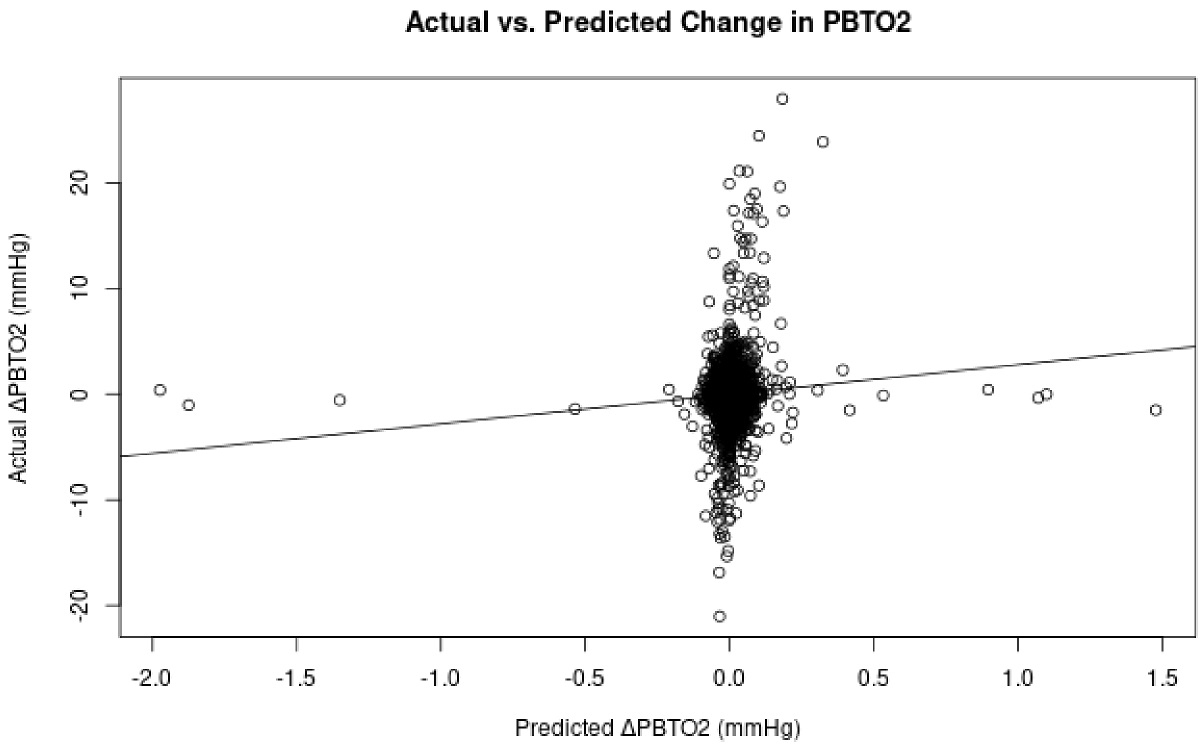

Supplementary File S3 Figure 17: Scatter plot of actual of actual vs predicted values of ΔPbtO2 for Subject 17.

Subject 18

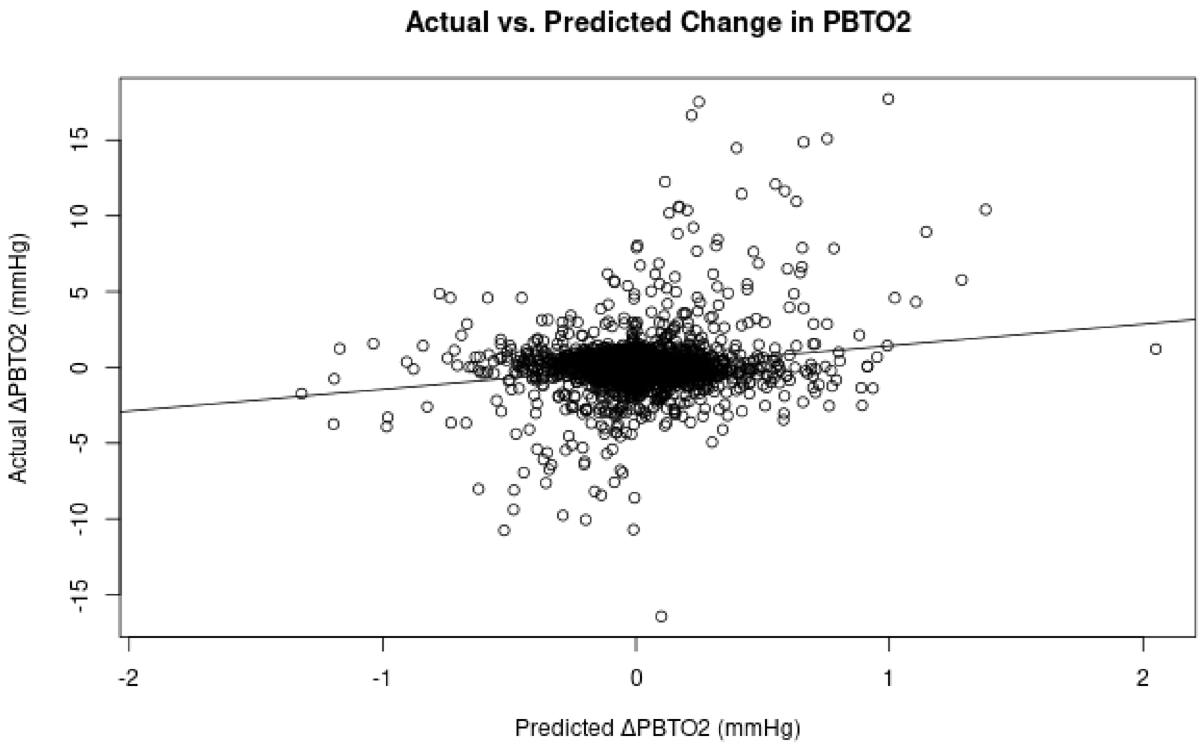

Supplementary File S3 Figure 18: Scatter plot of actual of actual vs predicted values of ΔPbtO2 for Subject 18.

Subject 19

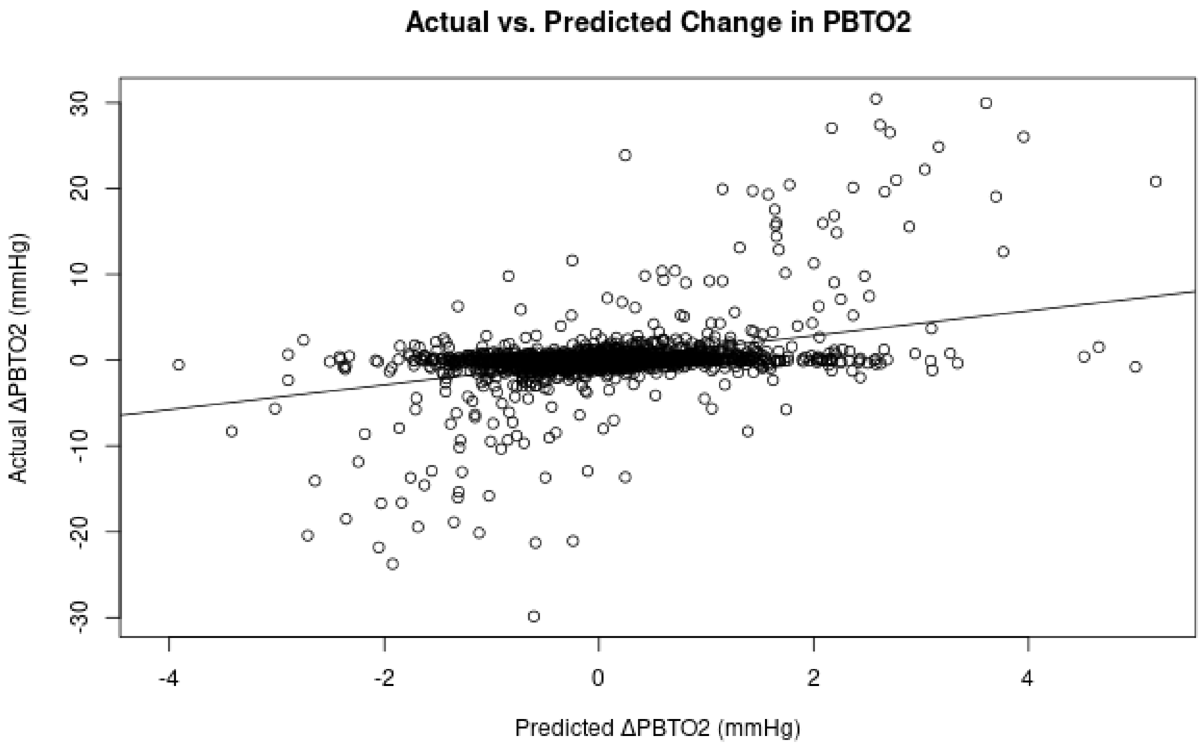

Supplementary File S3 Figure 19: Scatter plot of actual of actual vs predicted values of ΔPbtO2 for Subject 19.

Subject 20

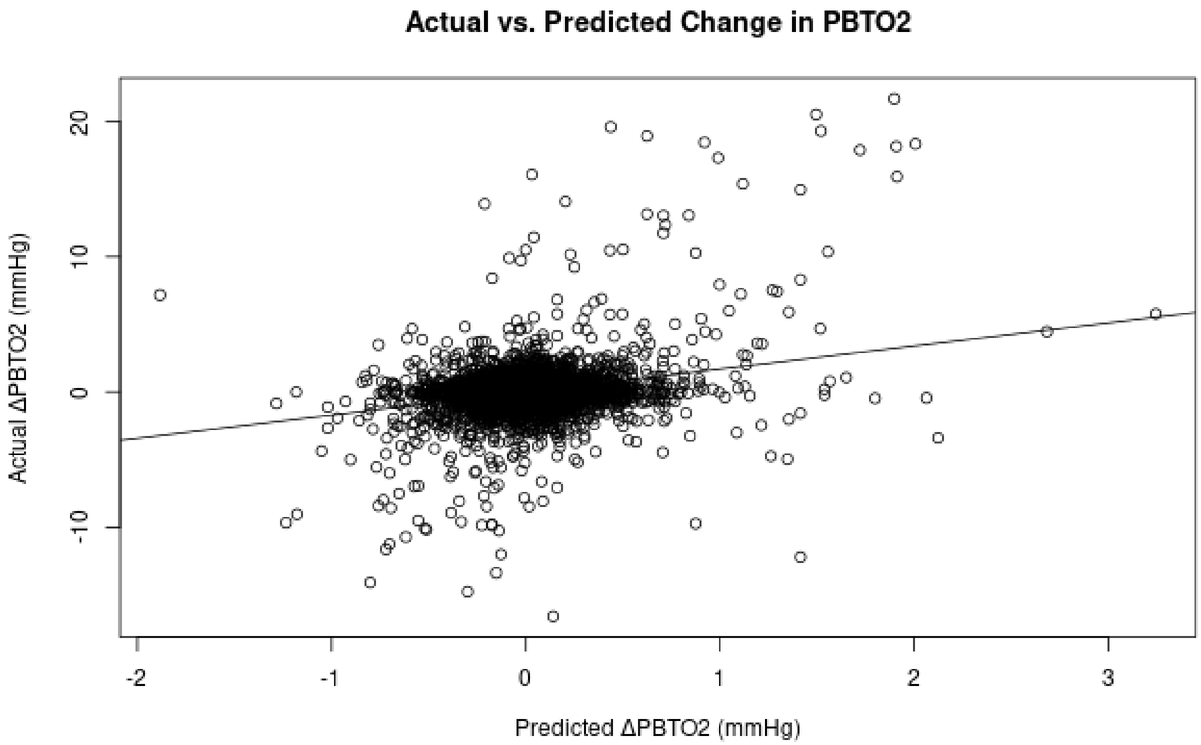

Supplementary File S3 Figure 20: Scatter plot of actual of actual vs predicted values of ΔPbtO2 for Subject 20.

4 Supplementary File S4: Bland-Altman plots to evaluate model agreement

Plot Legend: *ΔPbtO2 = change in brain tissue oxygen tension.*

Subject 1

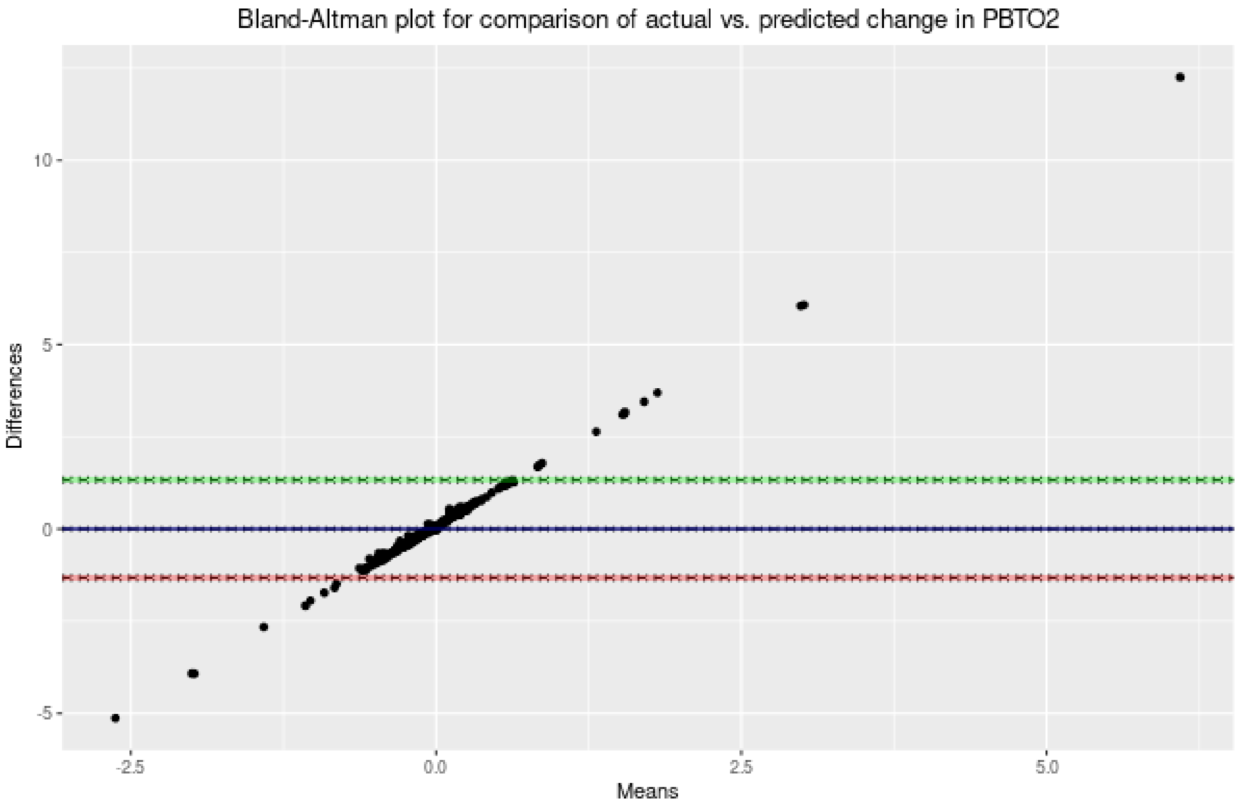

Supplementary File S4 Figure 1: Bland-Altman Plot comparing agreement between predicted values of $\Delta PbtO_2$ and actual values of $\Delta PbtO_2$ for Subject 1

Subject 2

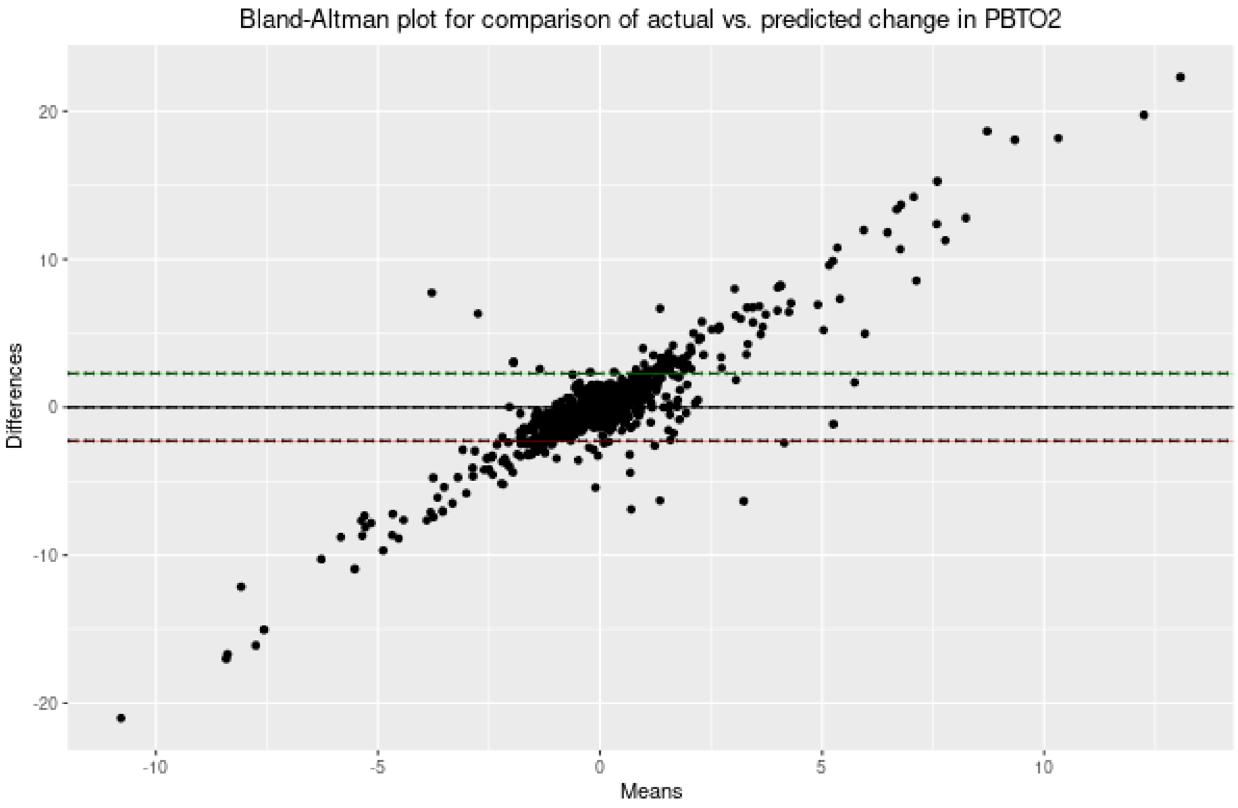

Supplementary File S4 Figure 2: Bland-Altman Plot comparing agreement between predicted values of $\Delta PbtO_2$ and actual values of $\Delta PbtO_2$ for Subject 2

Subject 3

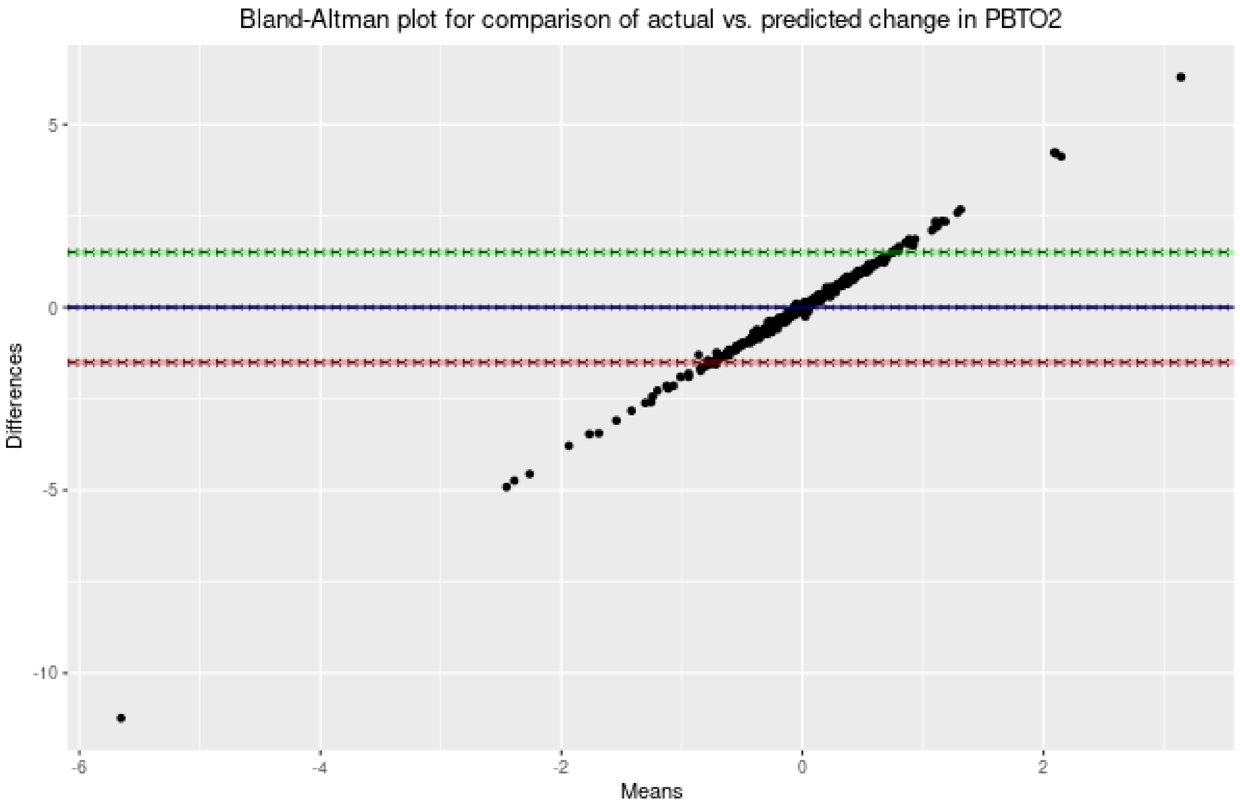

Supplementary File S4 Figure 3: Bland-Altman Plot comparing agreement between predicted values of $\Delta PbtO_2$ and actual values of $\Delta PbtO_2$ for Subject 3

Subject 4

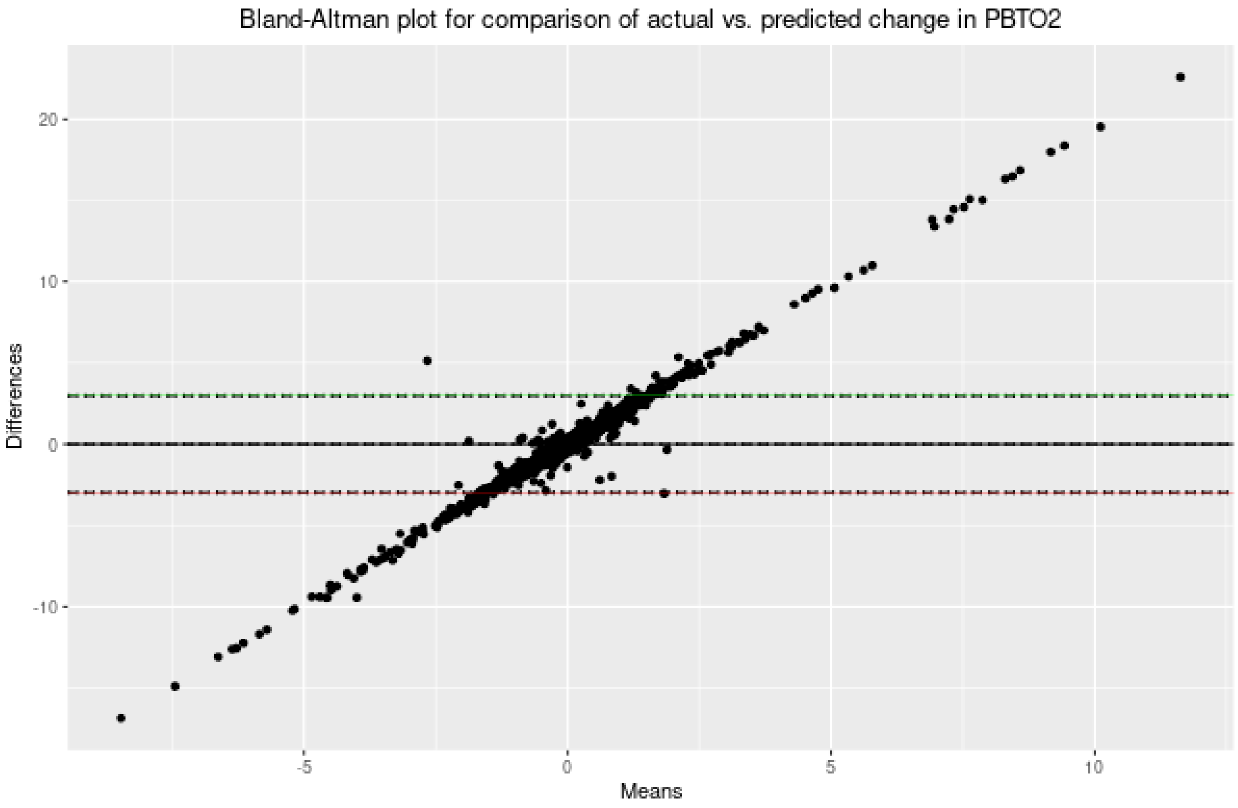

Supplementary File S4 Figure 4: Bland-Altman Plot comparing agreement between predicted values of $\Delta PbtO_2$ and actual values of $\Delta PbtO_2$ for Subject 4

Subject 5

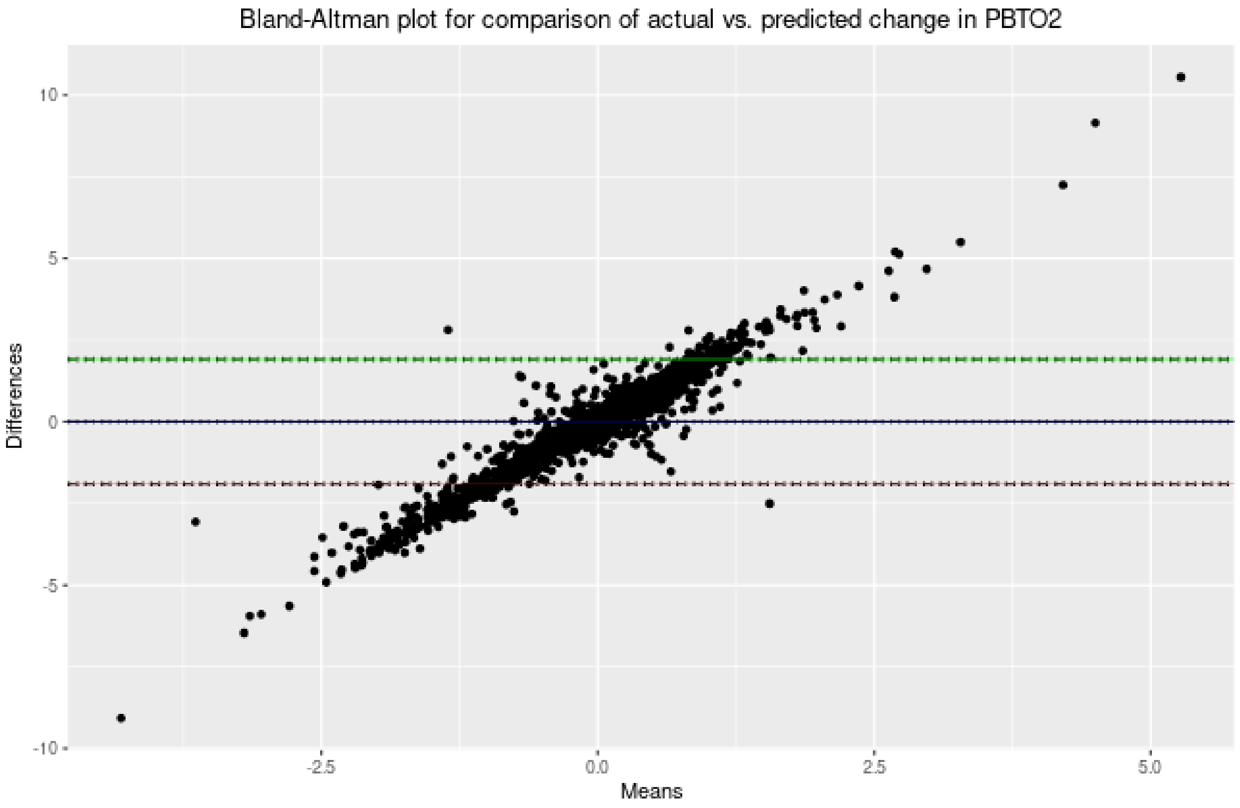

Supplementary File S4 Figure 5: Bland-Altman Plot comparing agreement between predicted values of $\Delta PbtO_2$ and actual values of $\Delta PbtO_2$ for Subject 5

Subject 6

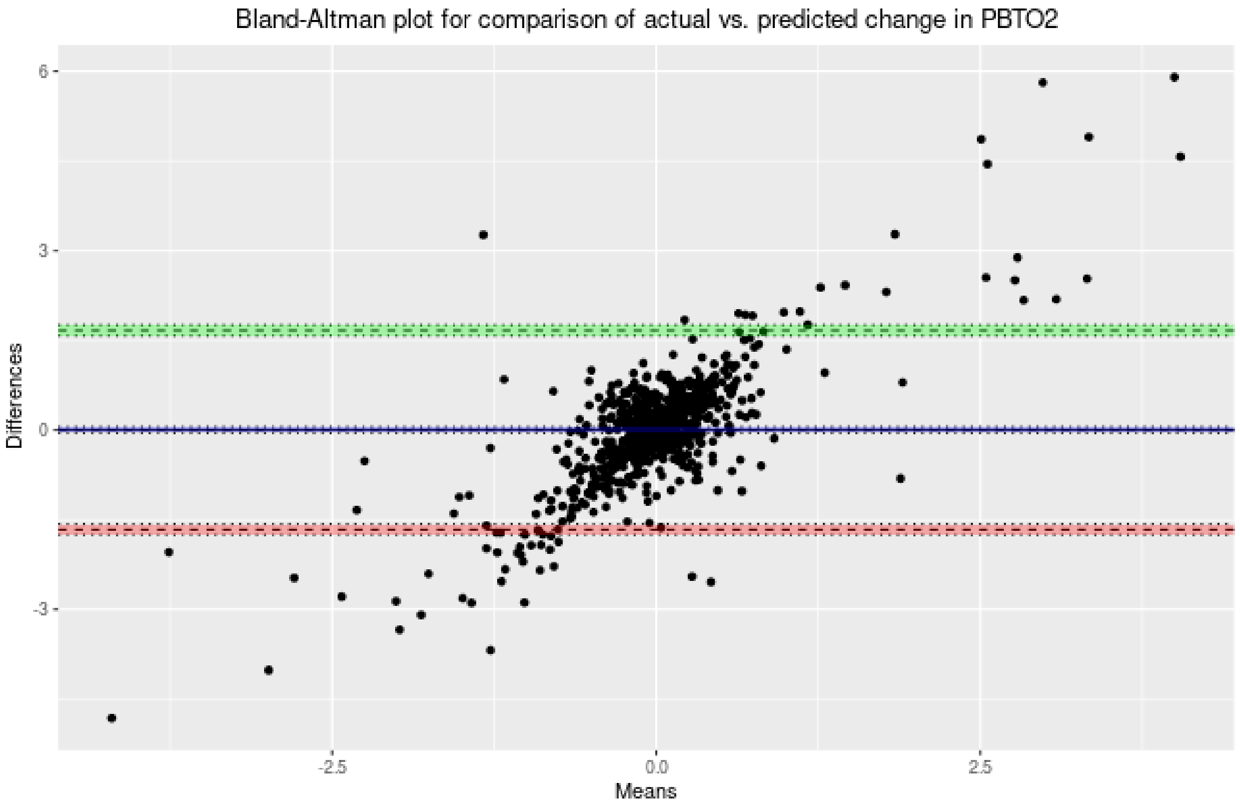

Supplementary File S4 Figure 6: Bland-Altman Plot comparing agreement between predicted values of $\Delta PbtO_2$ and actual values of $\Delta PbtO_2$ for Subject 6

Subject 7

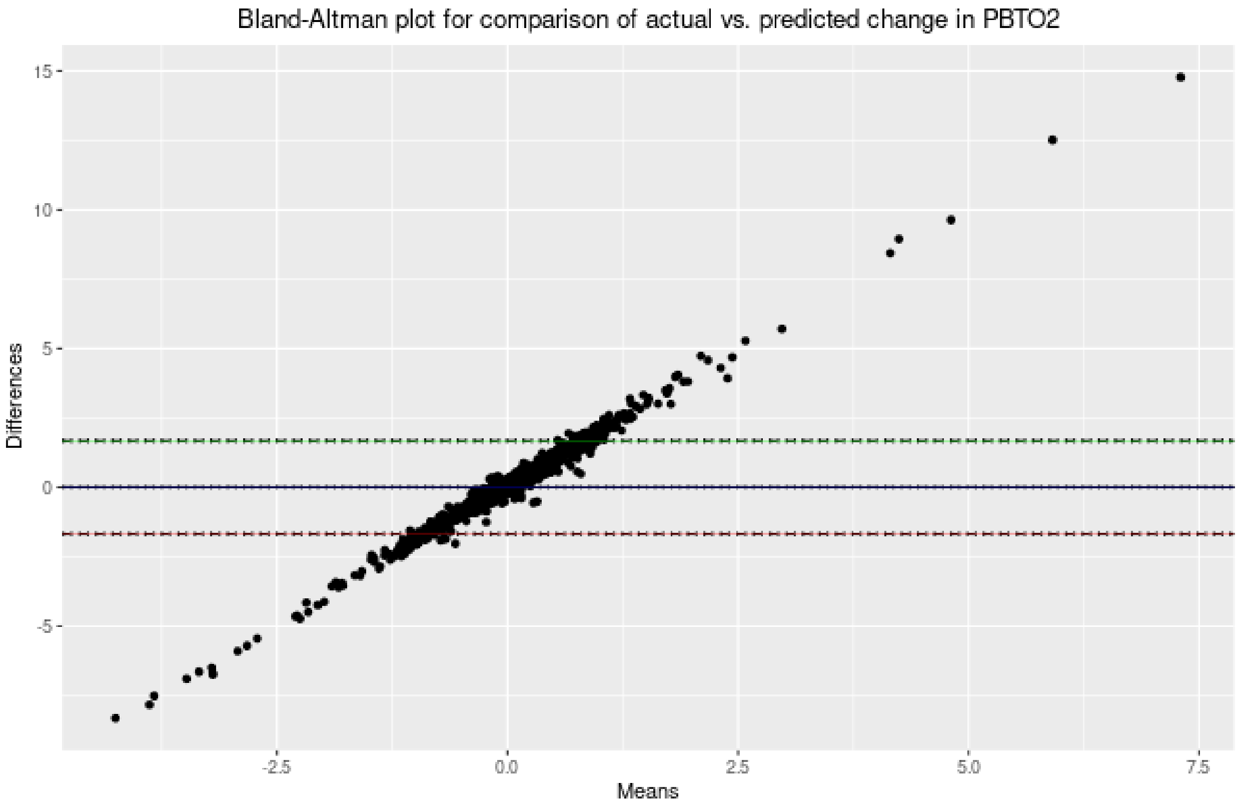

Supplementary File S4 Figure 7: Bland-Altman Plot comparing agreement between predicted values of $\Delta PbtO_2$ and actual values of $\Delta PbtO_2$ for Subject 7

Subject 8

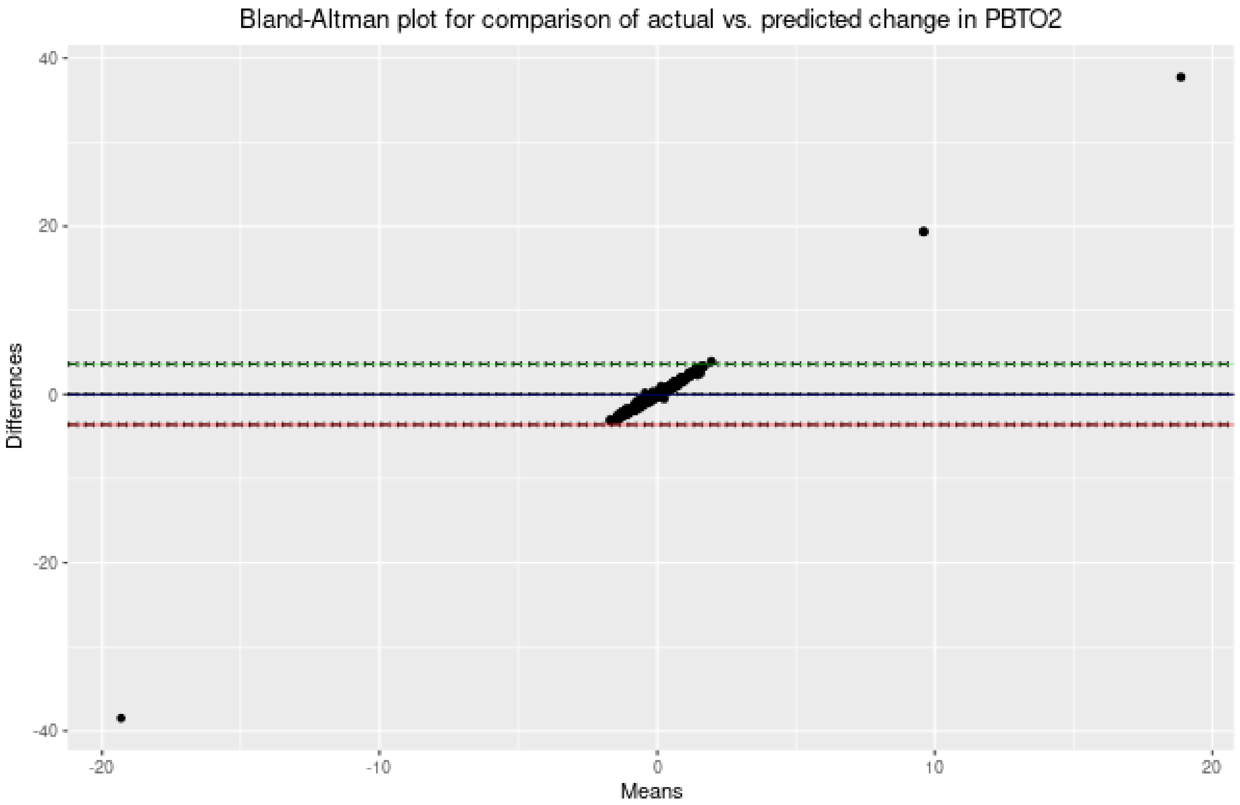

Supplementary File S4 Figure 8: Bland-Altman Plot comparing agreement between predicted values of $\Delta PbtO_2$ and actual values of $\Delta PbtO_2$ for Subject 8

Subject 9

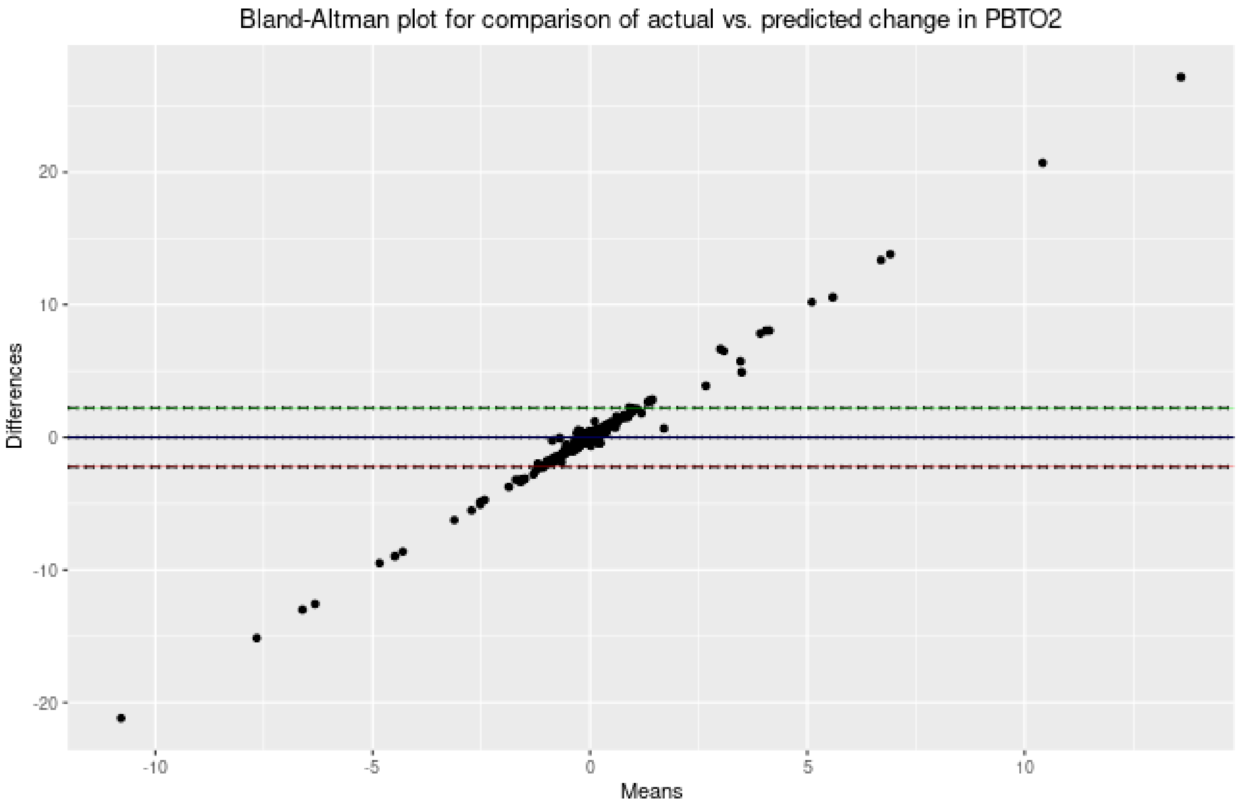

Supplementary File S4 Figure 9: Bland-Altman Plot comparing agreement between predicted values of $\Delta PbtO_2$ and actual values of $\Delta PbtO_2$ for Subject 9

Subject 10

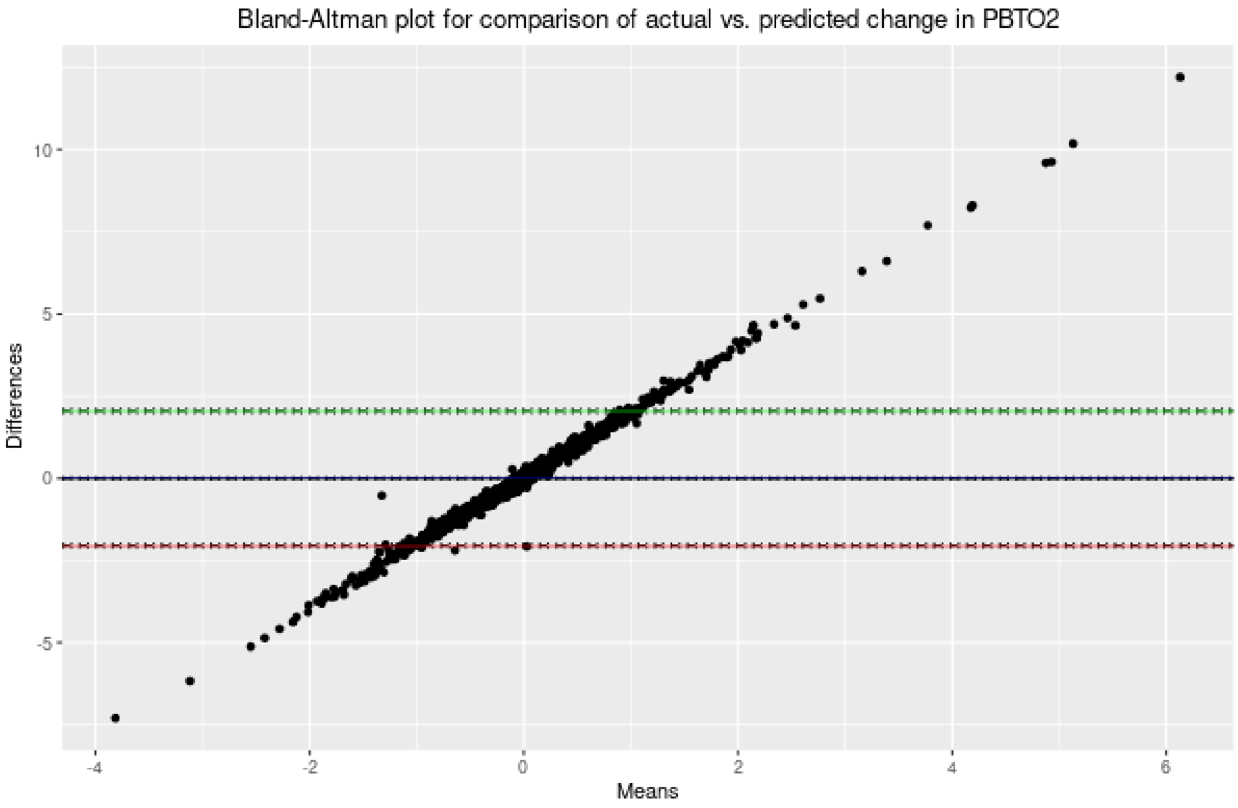

Supplementary File S4 Figure 10: Bland-Altman Plot comparing agreement between predicted values of $\Delta PbtO_2$ and actual values of $\Delta PbtO_2$ for Subject 10

Subject 11

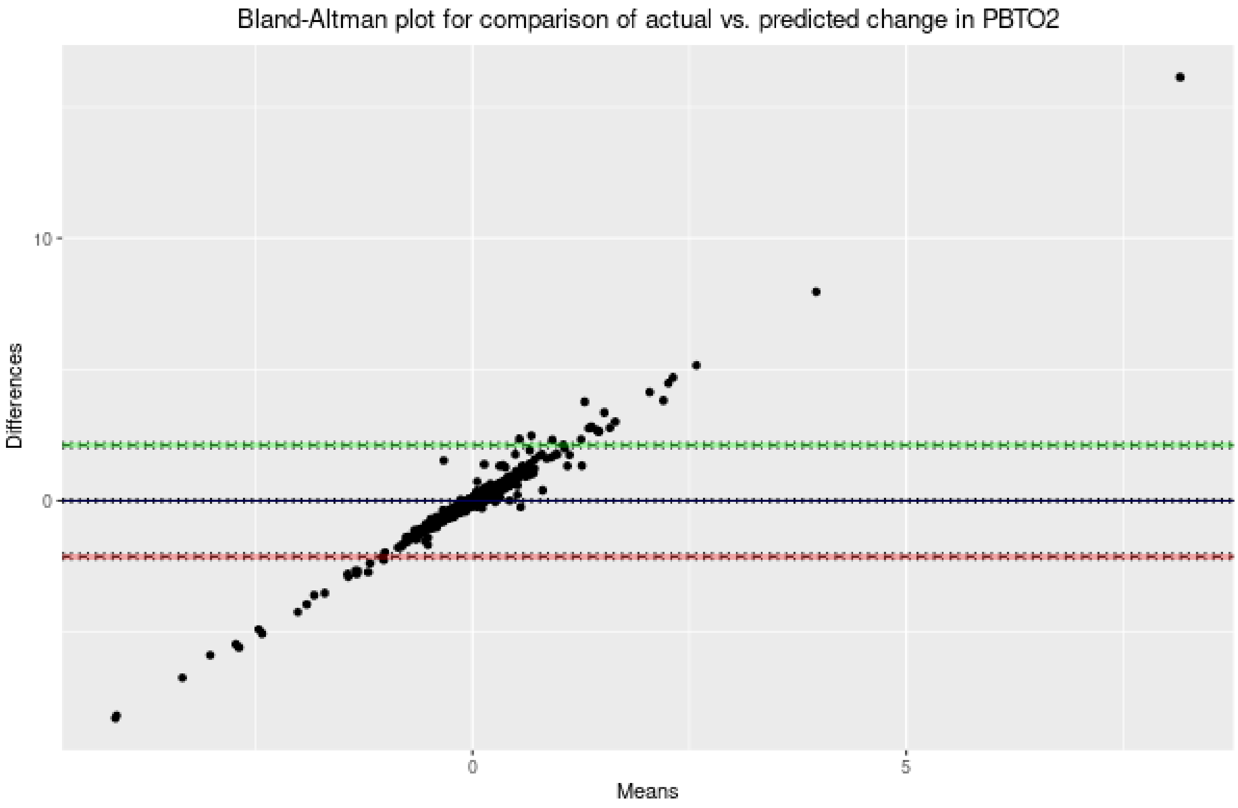

Supplementary File S4 Figure 11: Bland-Altman Plot comparing agreement between predicted values of $\Delta PbtO_2$ and actual values of $\Delta PbtO_2$ for Subject 11

Subject 12

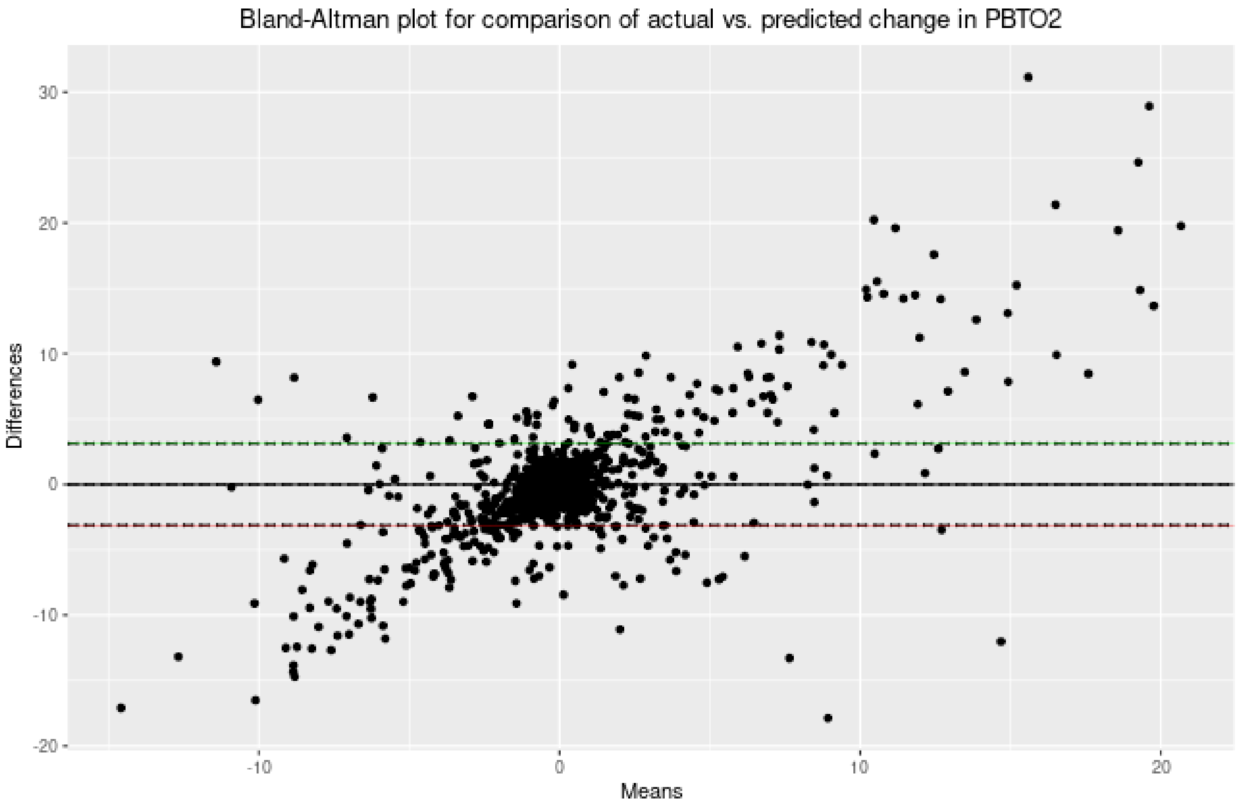

Supplementary File S4 Figure 12: Bland-Altman Plot comparing agreement between predicted values of $\Delta PbtO_2$ and actual values of $\Delta PbtO_2$ for Subject 12

Subject 13

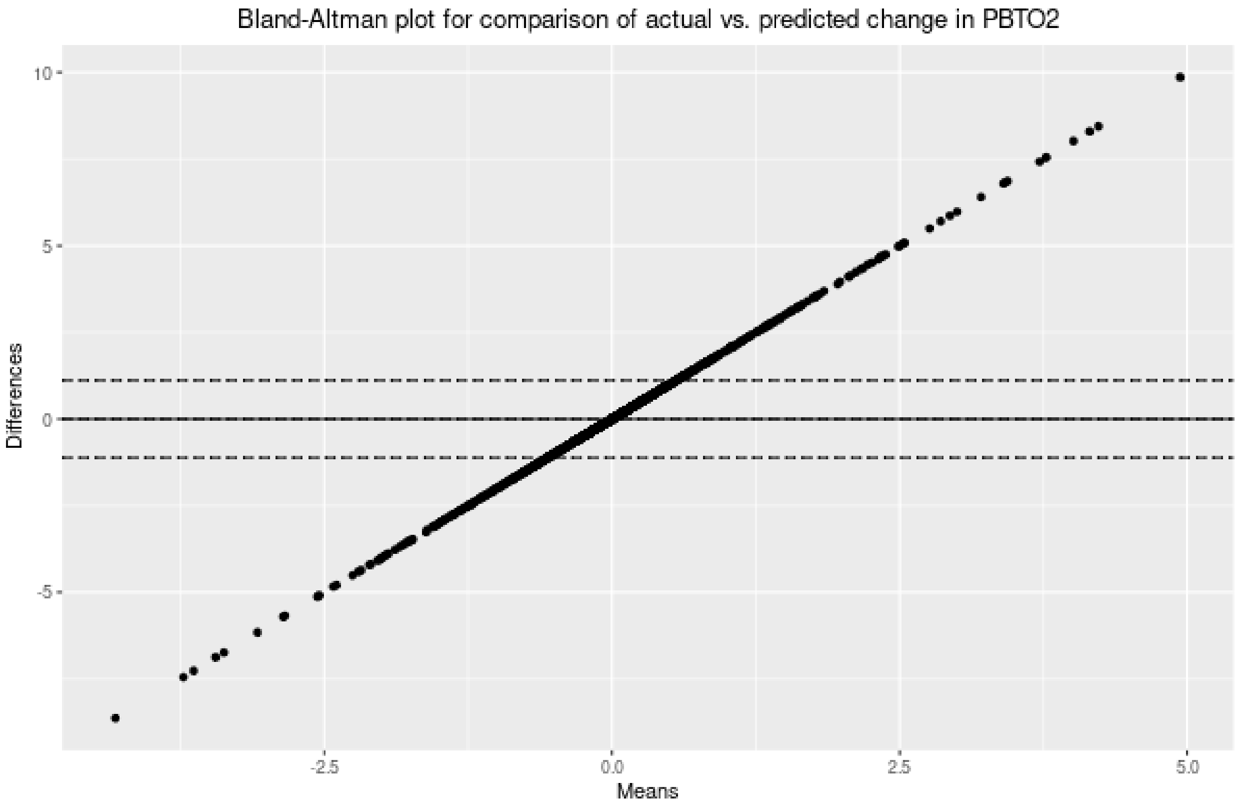

Supplementary File S4 Figure 13: Bland-Altman Plot comparing agreement between predicted values of $\Delta PbtO_2$ and actual values of $\Delta PbtO_2$ for Subject 13

Subject 14

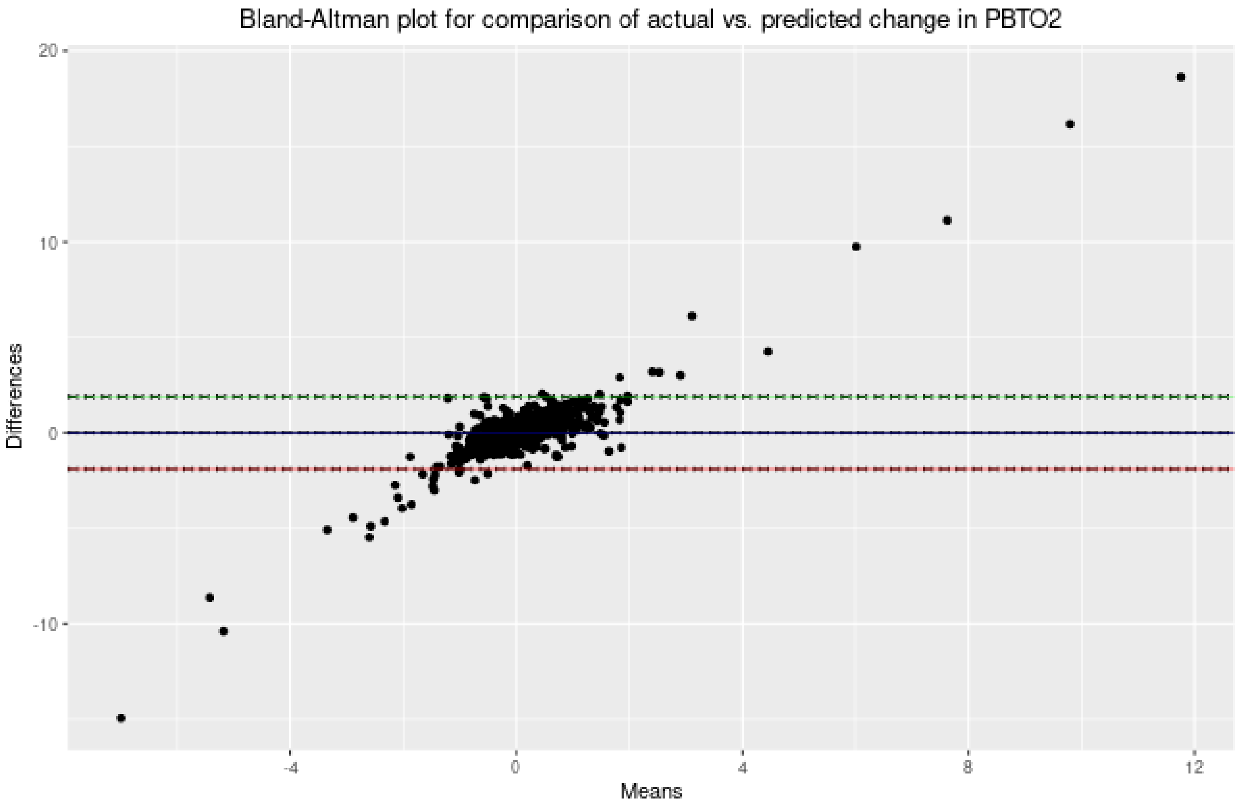

Supplementary File S4 Figure 14: Bland-Altman Plot comparing agreement between predicted values of $\Delta PbtO_2$ and actual values of $\Delta PbtO_2$ for Subject 14

Subject 15

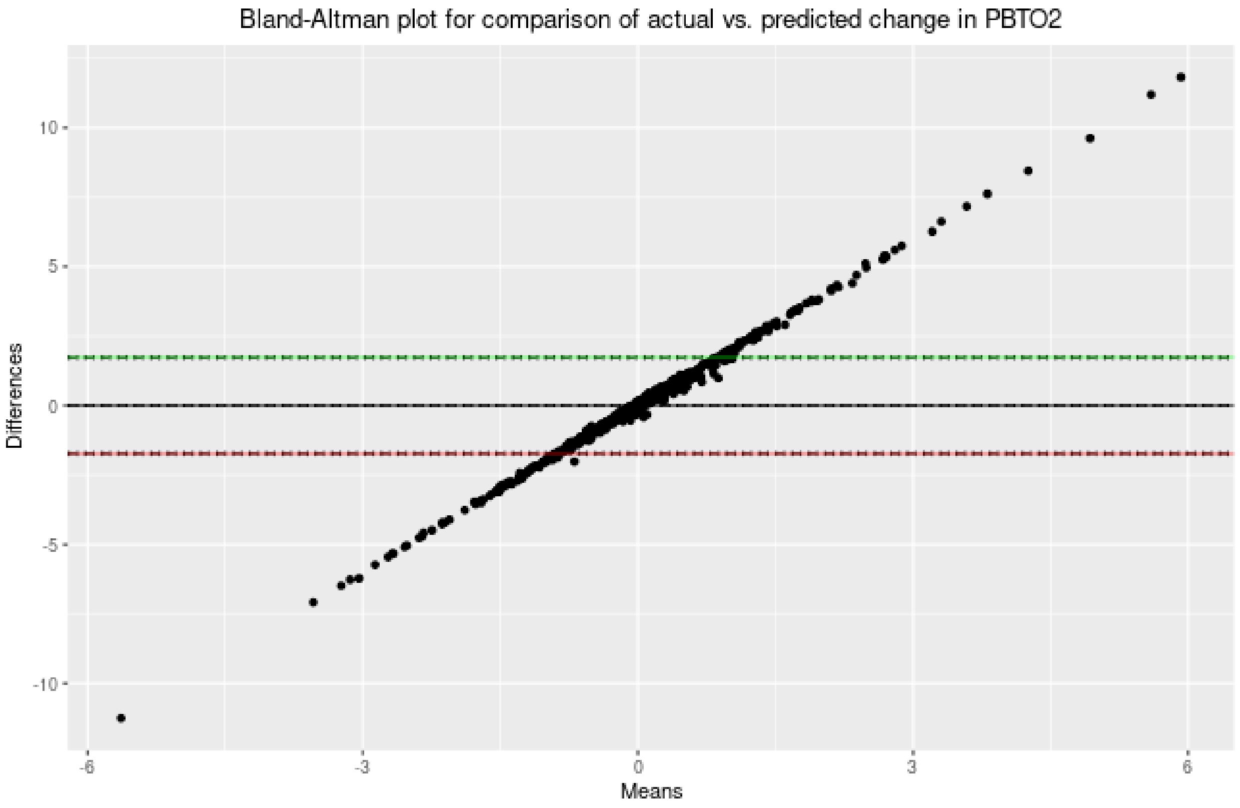

Supplementary File S4 Figure 15: Bland-Altman Plot comparing agreement between predicted values of $\Delta PbtO_2$ and actual values of $\Delta PbtO_2$ for Subject 15

Subject 16

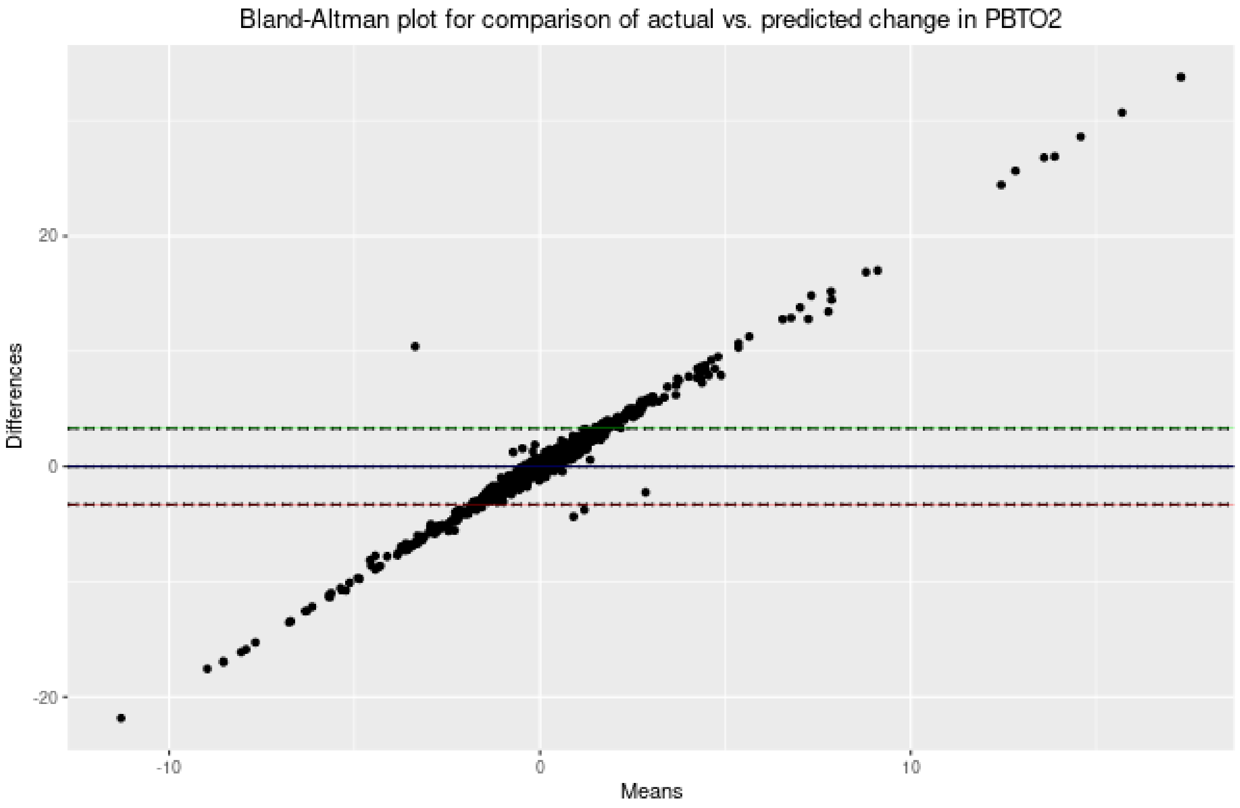

Supplementary File S4 Figure 16: Bland-Altman Plot comparing agreement between predicted values of $\Delta PbtO_2$ and actual values of $\Delta PbtO_2$ for Subject 16

Subject 17

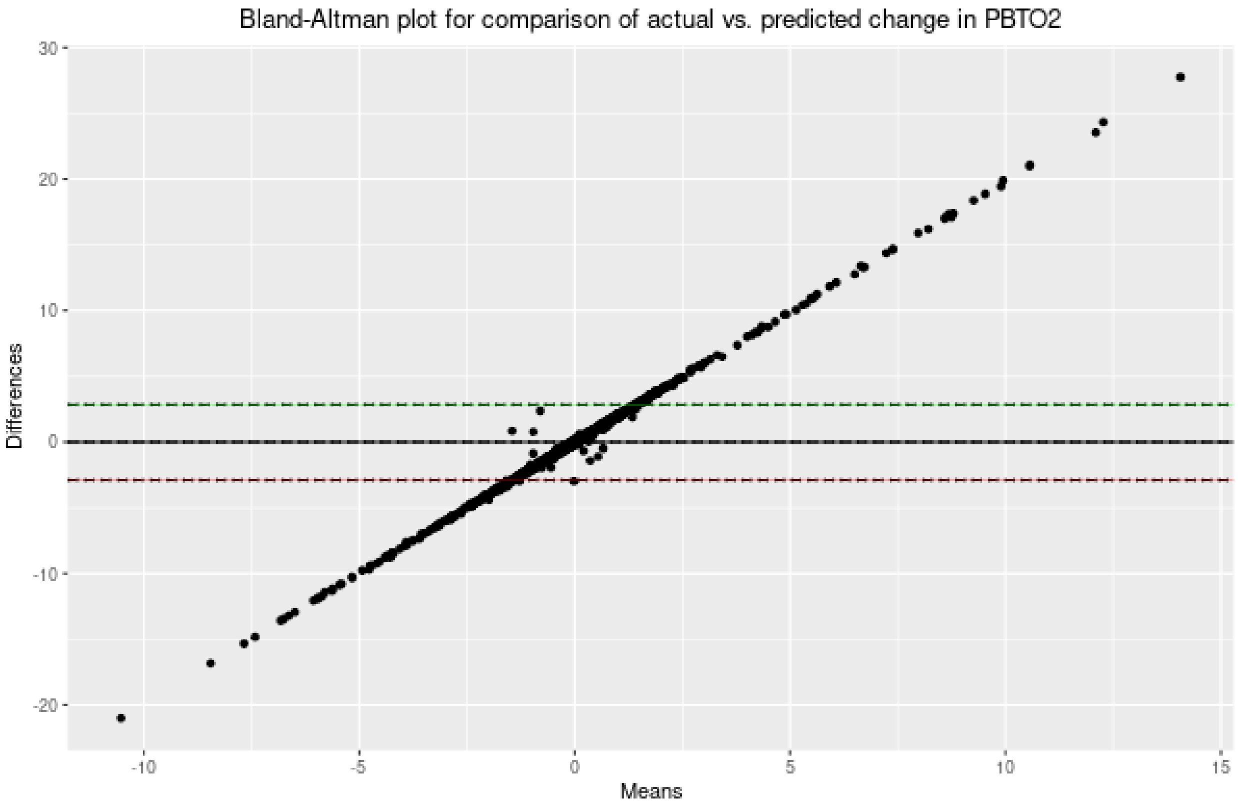

Supplementary File S4 Figure 17: Bland-Altman Plot comparing agreement between predicted values of $\Delta PbtO_2$ and actual values of $\Delta PbtO_2$ for Subject 17

Subject 18

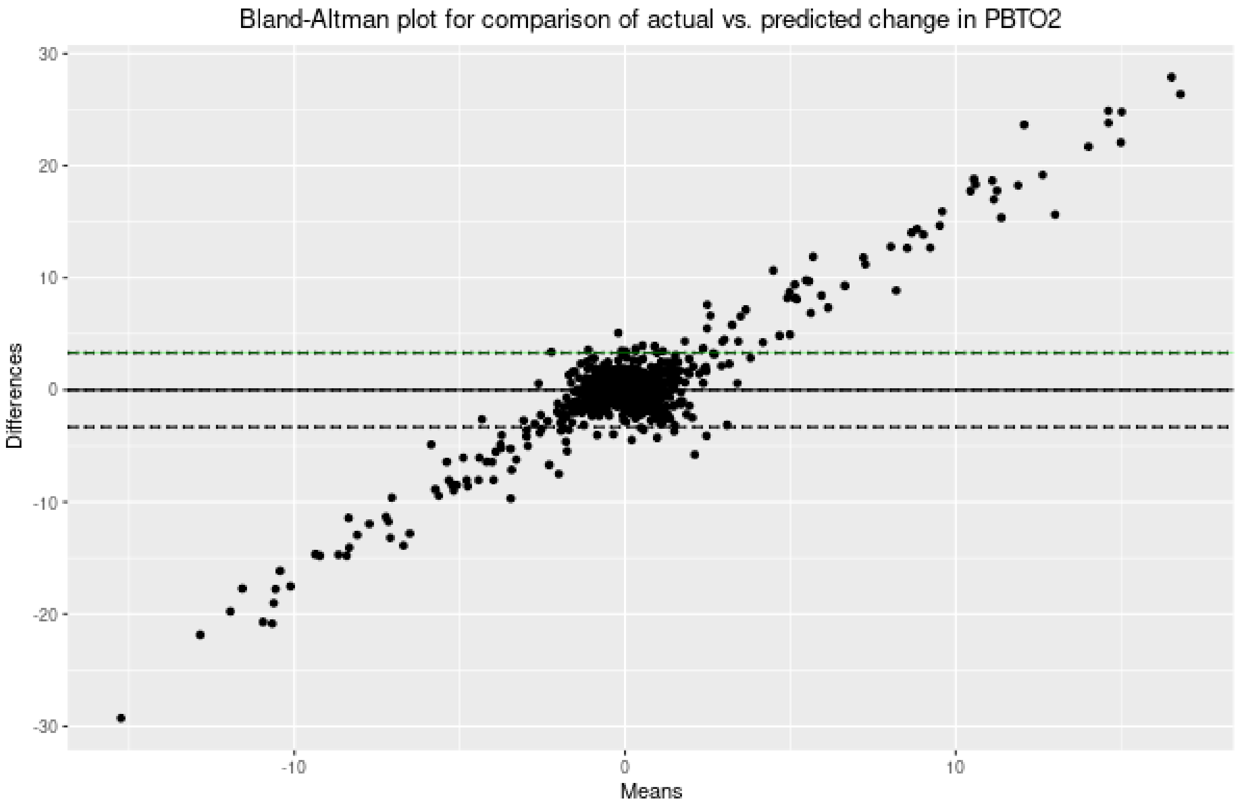

Supplementary File S4 Figure 18: Bland-Altman Plot comparing agreement between predicted values of $\Delta PbtO_2$ and actual values of $\Delta PbtO_2$ for Subject 18

Subject 19

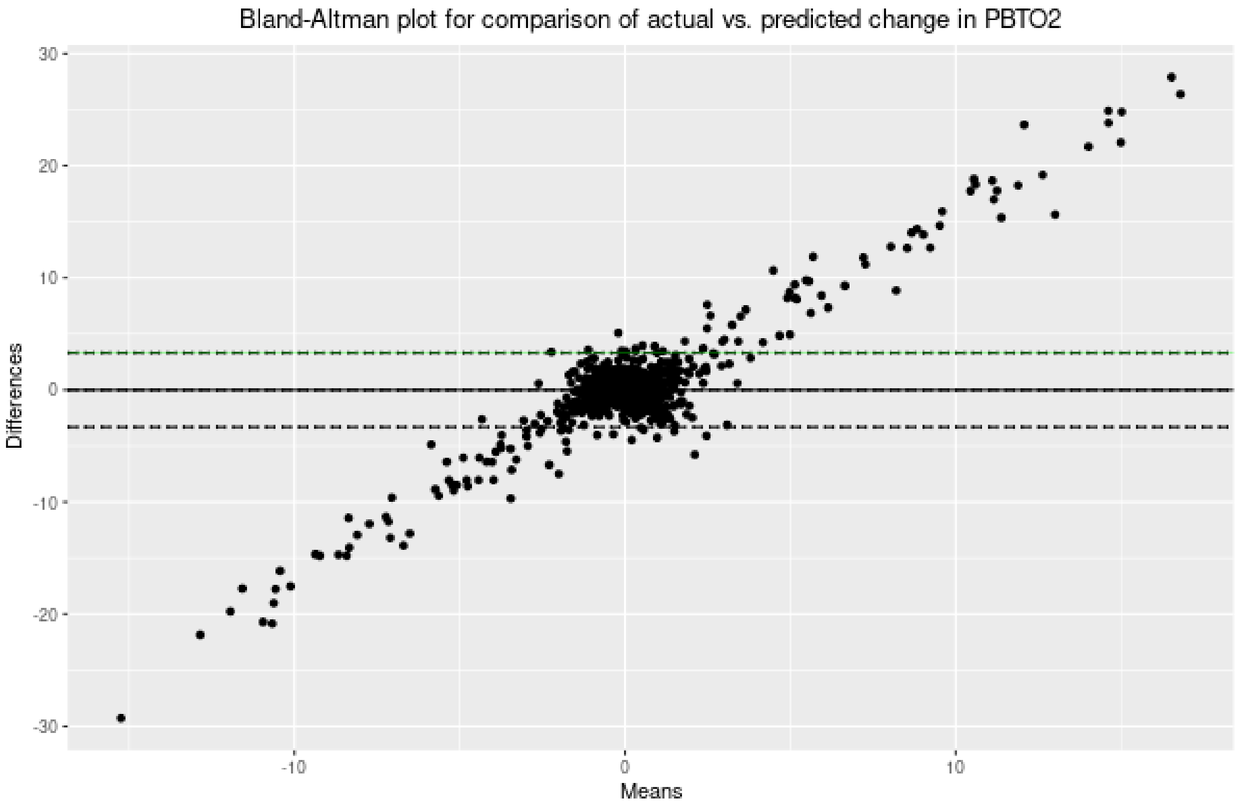

Supplementary File S4 Figure 19: Bland-Altman Plot comparing agreement between predicted values of $\Delta PbtO_2$ and actual values of $\Delta PbtO_2$ for Subject 19

Subject 20

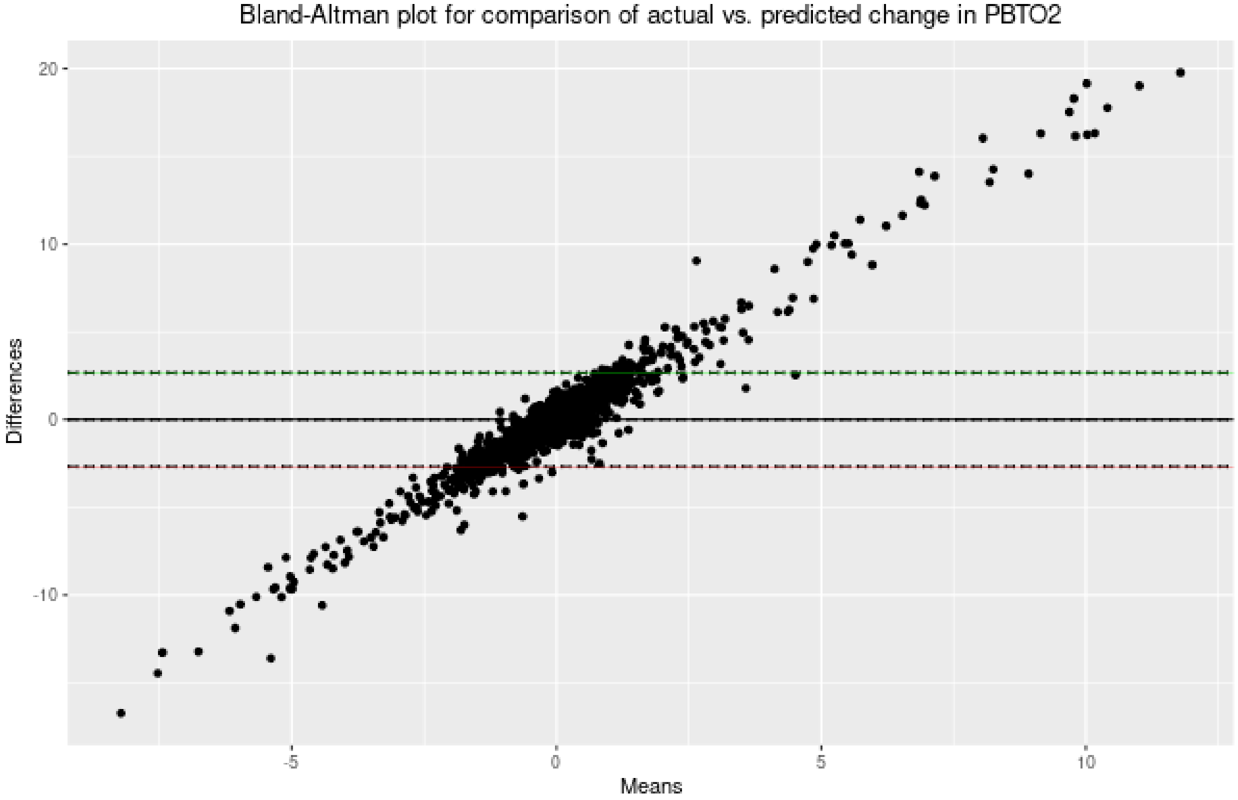

Supplementary File S4 Figure 20: Bland-Altman Plot comparing agreement between predicted values of $\Delta PbtO_2$ and actual values of $\Delta PbtO_2$ for Subject 20
